# Genetic characterization of populations in the Marquesas Archipelago in the context of the Austronesian expansion

**DOI:** 10.1038/s41598-022-08910-w

**Published:** 2022-03-29

**Authors:** Kai Tätte, Ene Metspalu, Helen Post, Leire Palencia-Madrid, Javier Rodríguez Luis, Maere Reidla, Erika Tamm, Anne-Mai Ilumäe, Marian M. de Pancorbo, Ralph Garcia-Bertrand, Mait Metspalu, Rene J. Herrera

**Affiliations:** 1 Estonian Biocentre, Institute of Genomics, University of Tartu, 51010 Tartu, Estonia; 2 BIOMICs Research Group, Lascaray Research Center, University of the Basque Country (UPV/EHU), 01006 Vitoria-Gasteiz, Spain; 3 Area de Antropología, Facultad de Biología, Universidad de Santiago de Compostela, Campus Sur s/n, 15782 Santiago de Compostela, Spain; 4 Department of Biology, University of Turku, 20014 Turku, Finland; 5 Department of Molecular Biology, Colorado College, Colorado Springs, CO 80903 USA

**Keywords:** Anthropology, Biological anthropology, Evolution, Genetics

## Abstract

Our exploration of the genetic constitution of Nuku Hiva (n = 51), Hiva Oa (n = 28) and Tahuata (n = 8) of the Marquesas Archipelago based on the analyses of genome-wide autosomal markers as well as high-resolution genotyping of paternal and maternal lineages provides us with information on the origins and settlement of these islands at the fringe of the Austronesian expansion. One widespread theme that emerges from this study is the genetic uniformity and relative isolation exhibited by the Marquesas and Society populations. This genetic homogeneity within East Polynesia groups is reflected in their limited average heterozygosity, uniformity of constituents in the Structure analyses, reiteration of complete mtDNA sequences, marked separation from Asian and other Oceanic populations in the PC analyses, limited differentiation in the PCAs and large number of IBD segments in common. Both the f3 and the Outgroup f3 results provide indications of intra-East Polynesian gene flow that may have promoted the observed intra-East Polynesia genetic homogeneity while ALDER analyses indicate that East Polynesia experienced two gene flow episodes, one relatively recent from Europe that coincides roughly with the European incursion into the region and an early one that may represent the original settlement of the islands by Austronesians. Median Network analysis based on high-resolution Y-STR loci under C2a-M208 generates a star-like topology with East Polynesian groups (especially from the Society Archipelago) in central stem positions and individuals from the different populations radiating out one mutational step away while several Samoan and outlier individuals occupy peripheral positions. This arrangement of populations is congruent with dispersals of C2a-M208 Y chromosomes from East Polynesia as a migration hub signaling dispersals in various directions. The equivalent ages of the C2a-M208 lineage of the populations in the Network corroborate an east to west flow of the most abundant Polynesian Y chromosome.

## Introduction

### Historical background

**Figure 1:**
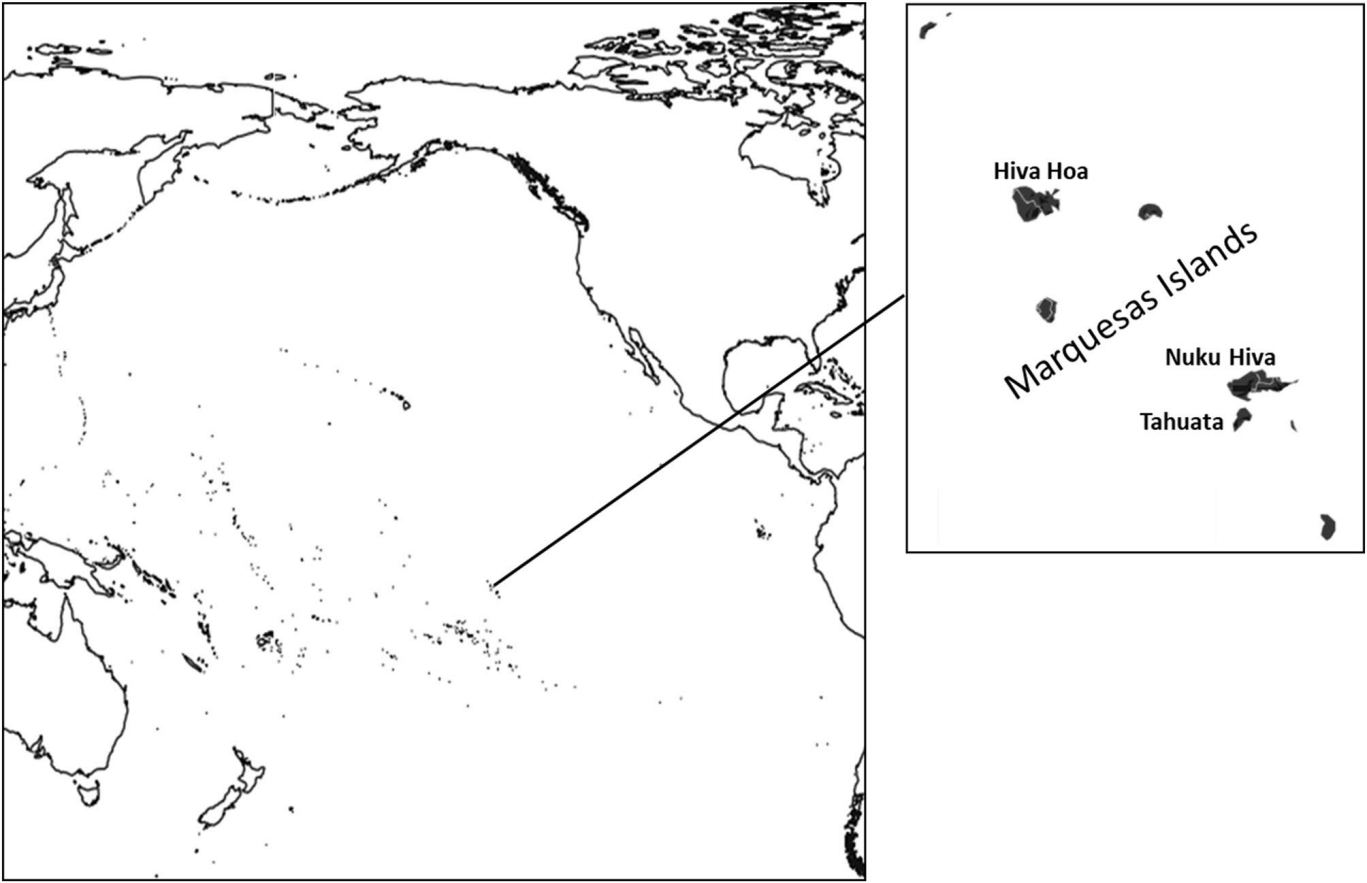
Map of the Pacific Ocean including the location of the Marquesas Archipelago and the location of the collections sites. Map was created using https://www.tableau.com/.

On the 21 of July 1595 some four hundred Polynesians came out in canoes to meet Álvaro de Mendaña de Neira in his flagship San Geronimo as the first Europeans landed on the Marquesas during their 1595–1596 voyage of discovery. It was reported by the navigator of the expedition that two weeks later about 200 natives had been killed^1^. It is approximated that at the time of first contact about 80,000 people inhabited the Marquesas located about 1,500 km northeast of Tahiti^2^ (Fig. 1). The Marquesas Archipelago lost more people to death from infectious diseases transported by Europeans than any other island group in Polynesia reaching an all time low of just over 2,000 by the beginning of the twentieth century^2^. It is likely that this dramatic drop in population size brought about a reduction in genetic heterogeneity.

### Settlement of the Marquesas

Recent information from archeological and paleoclimatic data indicate that human dispersal from islands to the west into East Polynesia including the Cook as well as the Austral, Society, Tuamotu and Marquesas Archipelagos started around AD 900^3^. Although the exact location of departure has not been delineated, a number of sources such as the Solomon, Tongan and Samoan Archipelagos have been proposed^4^. Radiometric determinations indicate that the Marquesas Archipelago was settled about AD 116–1256^5^. It is theorized that an initial landfall and return voyage was followed by colonization a century or two later. This step-wise process leading to settlement likely involved the acquisition of critical maritime knowledge including winds, direction of currents, landing sites and location of resources such as fresh water in the new land. Uniformity in culture, tradition and language indicate the travelers were closely related people from a common source area instead of a migration of individuals from numerous unrelated western sources.

The Marquesas Archipelago is unique in terms of the large number of early sites relative to other archipelagos in East Polynesia. There are about three times as many sites in the Marquesas compared to other islands in East Polynesia^6^. Although a number of factors may be responsible for the abundance of sites in the Marquesas including intensity of investigation and /or greater conservation/visibility, it has been suggested that the Marquesas have been occupied for a longer period of time^7^. Furthermore, the notion of a Marquesas homeland for East Polynesia has been advanced based on the notion that an earlier settlement of the archipelago could be the result of poor quality of shorelines and resources in the other archipelagos of East Polynesia prior to the sea level decline approximately 900 AD^8^. In other words, it is possible that the Marquesas were particularly attractive to early migrants due to their high elevation from sea level. Thus, they might have been settled prior to the more vulnerable flatter islands of East Polynesia to the west such as the Society Archipelago^9^. This scenario would suggest a leapfrog pattern of dispersal from the west instead of an advancing wave mechanism.

### Genetic background

Unlike many areas of anthropological research the general model of the peopling of the Pacific by Austronesians generated from archaeological and linguistic data is generally corroborated by genetic findings. The putative migration arc that traces a dispersal of Asian people from the island of Taiwan into Oceania by way of the Philippine Archipelago, Indonesia and Melanesia that culminated in the settlement of what is known today as Polynesia is for the most part congruent with genetic data^10^. High resolution genomic DNA, blood proteins, globin genes, uniparental DNA markers, commensal animal models and gut bacteria all support the standard model of human dispersal of the Pacific^11^.

Furthermore, genetic research has had a marked contribution to the understanding of human dispersal across the Pacific. One notable finding is the previously unknown sex-specific patterns of gene flow observed during the Austronesian expansion. Although genetic signals of Austronesian-Melanesian bidirectional interactions have been observed in the genome-wide and uniparental genomes of Melanesian and Polynesian populations, the input of Asian DNA into Melanesia was limited. Whole-genome Austronesian genetic signature is never higher than 20% and is observed only in less than 50% of the islands that speak Austronesian languages and never seen in Papuan-speaking populations^12^. In northern Melanesian islands, the uniparental Austronesian markers tend to be more abundant exhibiting 29.4–72.5% mtDNA and 5.3–37.7% Y-chromosome Asian DNA^13^. The observed difference between the abundance of Austronesian mtDNA and Y-chromosome DNA is likely the result of the patrilocal family system of the original Melanesian populations that the migrant encountered during their trek eastward^13^. In addition, the overall limited Austronesian genetic contribution seen in Melanesia suggests that Austronesians were temporary settlers in Melanesia as they dispersed into Oceania.

The contribution of Papuan DNA to the migrating Austronesians paints a different picture. In the West Polynesian Archipelagos of Samoa and Tonga, for example, autosomal STR markers are approximately 24% and 76%, and 35% and 65% of Melanesia and Asian descent, respectively^14^. Uniparental markers, on the other hand, exhibit a different profile depending whether Y-chromosome or mtDNA markers are employed. When mtDNA is examined, Asian DNA is about 93.8% and Melanesian is 6% among Polynesian populations while 65.8% and 28.3% of Y chromosomes are Papuan and Asian, respectively^13^. This dichotomy reflected in the uniparental markers has been attributed to the matrilocal family system of Polynesians^15^ and/or male-driven Papuan dispersal into Polynesian islands subsequent to the initial settlement. In support of this possibility, recent studies have demonstrated that Oceanic islands such as Vanuatu have been the recipient of at least three migrations, the first involving primarily people of East Asian ancestry, the second, shortly afterward, primarily of Papuan ancestry and the third of migrants of Polynesian ancestry^16^.

**Figure 2:**
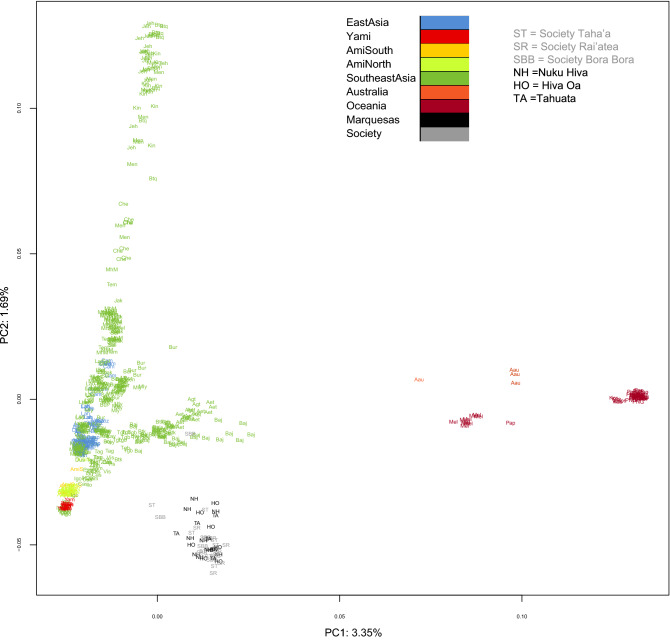
PC1/PC2 plot of East Polunesians and reference populations based on autosomal SNP loci. Color key to the populations examined is provided in Supplementary Table 3. Acronyms indicating populations are indicated adjacent to the individuals in the plot. Please refer to Supplementary Table 1 for specific populations examined within each region. *ST* Society Taha’a, *SR* Society Rai’atea, *SBB* Society Bora Bora, *NH* Nuku Hiva, *HO* Hiva Oa, *TA* Tahuata.

Using genome-wide SNP diversity previous research show that the Ami population of Taiwan shares strong genetic affinities with the Islands of Bora Bora and Rai’atea suggesting recent long distance dispersal from Island South East Asia (ISEA)^10^. Although structure analysis based on genome-wide SNP markers fails to identify the genetic components responsible for the IBD (identical by descend) signals from Taiwan in East Polynesia, clear contribution from Tonga and Samoa in West Polynesia is observed^4^. This robust connection between East Polynesia with West Polynesia is corroborated by high-resolution autosomal STR markers^17^. These genetic affinities seen in the structure analysis are diluted out in Vanuatu and Bougainville (Solomon Archipelago) located further west. In the islands of Sulawesi, Timor and Sumba of Indonesia the main structure component of West and East Polynesia are only minor constituents^4^. These observations are consistent with bottleneck, founder effect and genetic drift processes that likely culminated in the fixation of only two components in East Polynesia (Fig. 2 in reference 4) and extreme genetic homogeneity. These high-resolution genome-wide data are congruent with the well-established west to east Austronesian dispersal model responsible for the original settlement of Far East Oceania. In addition to this west to east Austronesian dispersal, recent studies have identified the signature of an ancient admixture event involving eastern Polynesian groups including the Marquesas and Native Americans from the northern coastal regions of South America^18^. The magnitude and frequency of such contacts are still to be determined.

Similarly, mtDNA markers in Polynesia exhibit strong affinities with ISEA and Taiwan, congruent with archaeological models^19^. Starting in the Solomon Islands, the frequency of haplogroup B (75%) exhibits a clinal increase from west to east^3^ while the B4 subhaplogroup accounts for almost all individuals within B in Fiji and B4a1a, B4a1a1a and their sublineages predominate in West Polynesia^20^. In Central Eastern Polynesia (Cook Islands) there is an increment in the B4a1a1m, B4a1a1m1 and B4a1a1c lineages compared to West Polynesia^3^, the later almost fixed in Marginal Eastern Polynesia (the Hawaiian Islands). In the Society Islands of East Polynesia, all mtDNAs are B4a1a1, most of the subhaplogroups belonging to the B4a1a1m1 and B4a1a1c lineages seen in Central Eastern Polynesia as well as a number of unique lineages such as B4a1a1k, B4a1a1t and B4a1a1a22. In Marginal Eastern Polynesia, all Maori of New Zealand are B4a1a1 and previously seen sublineages B4a1a1a and B4a1a1c as well as novel subhaplogroups such as B4a1a1a3 and B4a1a1a5^20^. The observed pattern of gradual diminution of genetic heterogeneity, fixation of specific subhaplogroups and the appearance of unique ones are indicators of random bottlenecks and founder events that marked the settlement of Eastern Polynesia out of Western Polynesia.

The phylogenetic signals provided by the Y chromosome in Polynesia are very different from the mtDNA indicators, with Melanesia lineages (C, K, M and S) predominating at frequencies ranging from 57 to 81% with no striking demic changes observed in any direction. In West Polynesia C2a-M208 is the most abundant lineage reaching 71% in the Samoan island of Manua and 81% in the Society island of Taha’a in East Polynesia^4^ while the Asian ancestry seen in the Y chromosome is made up mainly by O3-M122 and its sublineage O3a’i-P164 which constitutes 54% in Tonga, West Polynesia and 19% in the island of Taha’a of East Polynesia^4^.

### Aims of the study

The Marquesas Islands are the eastern-most archipelago of East Polynesia representing the extreme of a continuum of Pacific islands settled by Austronesian. As such, they provide us with the opportunity to examine the underlying mechanisms that led to their settlement from a genetic perspective. It also allows us to shed light on the origins of the population and the timing of their settlement. With this in mind, we provide information and analyses based on genome-wide autosomal as well as high-resolution genotyping of paternal and maternal lineages. In this study we aim to test if this group of islands at the eastern fringe of East Polynesia represents a population derived from West Polynesia whose genetic constitution was shaped by repeated bottleneck and founder effect events that have led to random drift and homozygosity.

## Materials and methods

### Sample collection, DNA extraction and quantitation

Buccal swabs were collected from a total of 87 unrelated male individuals from Nuku Hiva (n = 51), Hiva Oa (n = 28) and Tahuata (n = 8) in the Marquesas Archipelago of French Polynesia. As of 2017 the populations of these three islands were 3120, 2190 and 653, respectively. The samples were collected throughout the islands. Genealogical information was gathered for a minimum of two generations to confirm the descent of potential donors. Individuals were questioned to verify lack of familial links to other donors. Buccal swabs were processed utilizing the Gentra Buccal Cell Kit (Puregene, Gentra Systems, Minneapolis, MN) according to the manufacturers' specifications.

NanoDrop 1000 Spectrophotometer (Thermo Scientific) was used for DNA quantitation^21^. All samples were procured from donors voluntarily with informed consent while closely adhering to the ethical guidelines stipulated by Colorado College, Colorado Springs, Colorado USA and following the ethical principles and guidelines of the Declaration of Helsinki for the protection of human subjects. The IRB of Colorado College and the Research Ethics Committee of the University of Tartu, Estonia approved this study.

### Reference populations

Reference populations were employed for comparison across the mtDNA, Y-chromosome STR and autosomal SNP markers examined. The geographical locations, abbreviations used to define populations throughout the article, number of individuals and references are provided in Supplementary Table 1.

### Accession numbers and URLs

Eight novel complete mtDNA sequences are available online in GenBank at https://www.ncbi.nlm.nih.gov/ under accession numbers MT135508 – MT135515. The Y-chromosome data have been successfully submitted and are now included in the YHRD database at https://yhrd.org/ under the following accession numbers: Nuku-Hiva YA004253; Hiva-Oa YA004254. The eight donors from the island of Tahuata were reported as part of the Hiva-Oa population due to the geographical proximity of the two islands. The GenBank accession numbers for the SNP data are GSM5736361 – GSM5736384.

### Y-SNP analyses

All Nuku Hiva (n = 51), Hiva Oa (n = 28) and Tahuata (n = 8) chromosomes were genotyped using PCR and subsequent Sanger sequencing or restriction fragment length polymorphism analysis. Sequencing results were viewed in ChromasPro 2.1.8 (Technelysium, https://technelysium.com.au/wp/chromaspro/). The examined Y chromosomal biallelic markers examined were M45, M175, M122, P201, JST002611, P164, M188, Page 125, Page23, B451, B450, B435, F16950, M119, M110, B398, M110 (xB398), F31, F343 (xB386), F819 and B390. We designed primers for P164, M188, Page 125, Page23, B435, F16950, B398, M110 (xB398), F31, F343 (xB386), F819 and B390 using Primer3 software^22,23^. The designations of the Y chromosomal biallelic markers follow that of Karmin, et al.^24^. The specificity of the primers was checked with Primer-BLAST^25^ and GenomeTester v.1.3 software^26^ in silico and verified by Sanger sequencing. The O1 and O3 markers genotyped and the primer sequences designed for PCR amplification and sequencing are found in Supplementary Table 2.

### Y-STR analyses

Allelic frequencies for a total of 27 loci (DYS19, DYS385 a/b, DYS387S1 a/b, DYS389 I/II, DYS390, DYS391, DYS392, DYS393, DYS437, DYS438, DYS439, DYS448, DYS449, DYS456, DYS458, DYS460, DYS481, DYS518, DYS533, DYS570, DYS576, DYS627, DYS635 (Y GATA C4), and Y GATA H4) were accessed using the Yfiler plus multiplex PCR system (Applied Biosystems, Foster City CA). Amplification reactions were performed in an ABI PRISM GeneAmp 9700 Silver block Thermal Cycler (Life Technologies) using the 9600 emulation mode for 30 cycles. All analyses used the internal lane standard and the allelic ladder mix provided by the Y27-STR Yfiler plus system. PCR products were separated and detected on an ABI PRISM 3130xl Genetic Analyzer (Life Technologies) following manufacturer's specifications using the LIZ-120 internal size standard as a basis for comparison. Fragment sizes were assigned using the software GeneMapper v3.1 (Applied Biosystems, Foster City, CA) and alleles were designated by comparison to an allelic ladder supplied by the manufacturer (Applied Biosystems). Slatkin’s linearized Rst values^27^ were calculated using the ARLEQUIN 3.5 statistical package. Significance was assessed at *p* = 0.05. Based on Rst values, a multidimensional scaling (MDS) analysis was performed using PROXSCAL^28^ included in the SPSS version 20 statistical package (IBM Corp. Released 2011. IBM SPSS Statistics for Windows, Version 20.0. Armonk, NY: IBM Corp.).

### Median network analysis and age estimations

The 15-loci Y-STR haplotypes of Hiva Oa and Nuku Hiva from the Marquesas Archipelago, Rai’atea and Bora Bora from the Society Archipelago, American Samoa, Maoris and Ontong from the Solomon Archipelago belonging to the C2a-M208 sub-haplogroup were used to generate a Median-Joining Network (NETWORK 4.5.1.6 at http://www.fluxusengineering) in which the Y-STR markers were weighted inversely to their repeat variance and the Maximum Parsimony (MP) option was employed to produce the least complex topology.

Y-STR haplotypes were used to estimate the time to the most recent common ancestor (TMRCA) of the C2a-M208 sub-haplogroup in the populations examined in the Median-Joining Network analysis. With this aim, rho statistic (*ρ*)^29^ and weighted rho (*ρ*_W_)^30^ were estimated with an R script available in GitHub (http://github.com/fcalafell/weighted_rho). The number of repeats at DYS389II was calculated after subtracting the number of repeats at DYS389I. Mutation rates were obtained from the Y-Chromosome STR Haplotype Database (YHRD, www.yhrd.org) on March, 2020.

### mtDNA analyses

The DNA extracts of four Nuku Hiva, three Hiva Oa and one Tahuata were genotyped for mitochondrial haplogroups by complete mtDNA sequencing using Sanger sequencing and following the protocol of^31^ using rCRS (https://www.mitomap.org/MITOMAP/HumanMitoSeq) as a reference sequence^32^ and ISFG guidelines^33–35^. For sequence alignments the software package ChromasPro (Technelysium Pty Ltd, South Brisbane QLD 4101, Australia) was used. Nomenclature followed that of Phylotree. org, mtDNA tree Build 17^34^. A phylogenetic tree based on complete mtDNA sequences was generated using HaploGrep2^36^.

The mitochondrial haplogroup data was analyzed in conjunction with other from reference populations from Taiwan and the Society Islands of French Polynesia.

### Autosomal analyses

#### Genotyping

A set of 713,014 single nucleotide polymorphisms (SNPs) was screened in nine Nuku Hiva, nine Hiva Oa and six Tahuata healthy unrelated individuals using the Illumina OmniExpress Bead Chips array. For comparative purposes, a dataset of 701 samples from 49 previously published reference populations (Supplementary Table 1) was merged with the data from the 24 samples from the Nuku Hiva, Hiva Oa and Tahuata populations. The reference populations represent key locations in East Asia, SEA and Oceania. The Nuku Hiva, Hiva Oa,Tahuata and reference populations used are compatible with the Illumina genotyping arrays.

#### Data curation

All the samples were filtered with plink v1.9^37^. Only SNPs on autosomal chromosomes with a minor allele frequency > 1% and genotyping success rate > 97% were used in the analyses. Only individuals with a genotyping success rate > 97% were left in the sample set. A total of 240,757 SNPs and 725 individuals passed the filters. For PCA and ADMIXTURE analyses, the dataset was further processed by excluding SNPs with pairwise genotypic correlation of r^2^ > 0.4 in a window of 200 SNPs sliding the window by 25 SNPs at a time^38^. A total of 142,512 SNPs were left after this step.

#### Population structure analyses

Average heterozygosity for each population was calculated using plink v1.9. An autosomal PCA was performed using EIGENSOFT 6.1.4^39^ on the pruned dataset. To shed light on the Nuku Hiva, Hiva Oa and Tahuata’s genetic structure in the context of other East and SEA populations, structure analyses were run with the ADMIXTURE 1.23 program^40^ utilizing the random seed number generator on the same dataset, one hundred times for each number of ancestral populations (K = 2 to K = 18). Using cross-validation procedures, we found that at K = 12 the cross-validation error was the lowest (Supplementary Figs. 1 and 2).

#### Demographic inferences

A series of three- and four-population tests were performed with ADMIXTOOLS-1^41^. Standard f3 statistics were used as a formal test of admixture^42^ between all possible combinations of populations as sources with Nuku Hiva, Hiva Oa and Tahuata as target populations. Outcomes with significant negative values suggest admixture, while positive values are not informative. Also, f4 statistics (Nuku Hiva, Hiva Oa, Tahuata, Test population, Outgroup format) were used to test for admixture involving the reference populations. Significant positive values in the f4 analysis would mean that population 1 (eg, Nuku Hiva) shares ancestry with a given test population while significant negative values would indicate that population 2 (eg, Tahuata) shares ancestry with the test population. Nonsignificant values would mean that Nuku Hiva and Tahuata, Hiva Oa and Nuku Hiva or Hiva Oa and Tahuata are equally related to the Test population. In addition, the outgroup f3 test was implemented as a measure of the shared branch length between each of the three Marquesas populations and all the other populations. For outgroup in the f3 test, the Yoruba population from West sub-Sahara Africa was employed.

#### Haplotype-based analyses

Beagle 3.3.2^43^ was used first to phase and then to run Refined identity-by-descent (IBD)^44^ analyses, where we studied the sharing of DNA segments of IBD between the Nuku Hiva, Hiva Oa or Tahuata and the reference populations in our dataset. From the results, we extracted the count of segments shared between every two individuals and found population medians for 0–1 cM, 1–2 cM and > 2 cM segments to find patterns.

## Results

In this report we provide a new genomic dataset from the Nuku Hiva, Hiva Oa and Tahuata Islands of the Marquesas Archipelago in French Polynesia. The information includes high-resolution autosomal SNP genotyping data from 24 individuals (nine from Nuku Hiva, nine from Hiva Oa and six fromTahuata). In addition, we report the complete mtDNA of four Nuku Hiva, three Hiva Oa and one Tahuata individuals as well as the haplogroups of 51 Nuku Hiva, 28 Hiva Oa and eight Tahuata Y chromosomes. High-resolution Y-STR haplotypes were generated and utilized to assess male-driven relationships among Nuku Hiva, Hiva Oa and Tahuata Islands and key geographically targeted reference populations. Median Network analysis based on Y-STR loci was used to examine the relationship of C2a-M208 individuals in key Polynesian populations and outliers. The totality of the dataset is analyzed together with publicly available data from populations from MSEA, ISEA, Melanesia and Polynesia (Supplementary Table 1).

### Autosomal SNPs

#### PCAs

**Figure 3:**
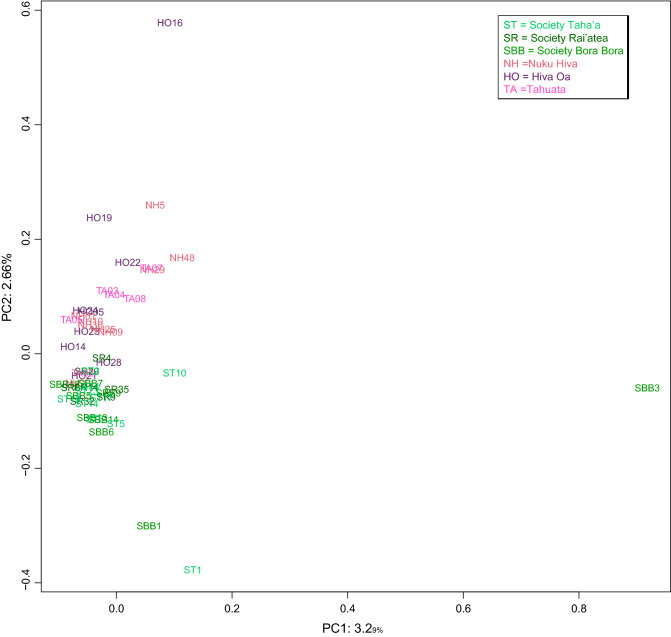
PC1/PC2 plot of East Polynesian populations. Designations for each individual are indicated adjacent to the individuals in the plot. *ST* Society Taha’a, *SR* Society Rai’atea, *SBB* Society Bora Bora, *NH* Nuku Hiva, *HO* Hiva Oa, *TA* Tahuata.

The first two components in the PCA plot based on the autosomal SNP genotyping data were used to analyze the Marquesas and the Society populations in the context of MSEA, ISEA, Melanesian groups (Fig. 2). A color key for the populations in the PCA plots is provided in Supplementary Table 3. The Nuku Hiva, Hiva Oa and Tahuata populations (in black and label with their acronyms) segregate separately from the reference groups into an isolated cluster of their own. The Marquesas groups plot distinctly with the Society Island populations (in grey) in an East Polynesian cluster in the lower right corner of the projection. Only one population from Bora Bora of the Society Archipelago segregates away from this East Polynesian clutch and with the ISEA groups. The Australian aborigines (orange) and Papua (dark red) populations plot distantly to the right of all the populations while the rest of the groups from the Philippines and Indonesia (light green) branch out in a fork-like configuration from a compact cluster of Ami (yellow) and Yami (red) aborigines from Taiwan and the Igorot population from north Luzon, Philippines. The Yami of Taiwan plots closer to the Igorot than to the Ami from Taiwan. Mainland East Asian groups (blue) are embedded within ISEA populations. To evaluate more clearly the relationship among East Polynesian populations, a separate PCA was performed including only the East Polynesian groups (Fig. 3). In this East Polynesian-specific plot a clear separation between the Marquesas (above) and the Society (below) populations is observed while one Hiva Oa (HO 16) and one Bora Bora (BB 3) individual segregate away from this East Polynesian cluster. From this East Polynesian-specific PCA, it is apparent that the Marquesas individuals plot more dispersedly from each other than the ones from the Society Island. A second PCA plot segregating the East Polynesians and the reference groups using instead the PC1 and PC3 (Supplementary Fig. 3) generated a distribution of populations similar to the one seen in the PC1/PC2 projection except that this time three Society individuals partitioned away from the East Polynesian clutch in close proximity to an Indonesian cluster, the ISEA grouping now exhibits some sub-structuring and the MSEA populations plotted at one end of an Indonesian cluster (Supplementary Fig. 3). In the PC1/PC3 projection of just East Polynesians (Supplementary Fig. 4) a number of individuals including BB 3 (Bora Bora), ST 1 (Taha’a), NH 29 and NH 48 (last two from Nuku Hiva) segregate as outliers from their compact cluster. In this East Polynesian-specific PCA, a greater separation of Marquesas individuals from the Society samples is also observed.

### Structure analyses

**Figure 4:**
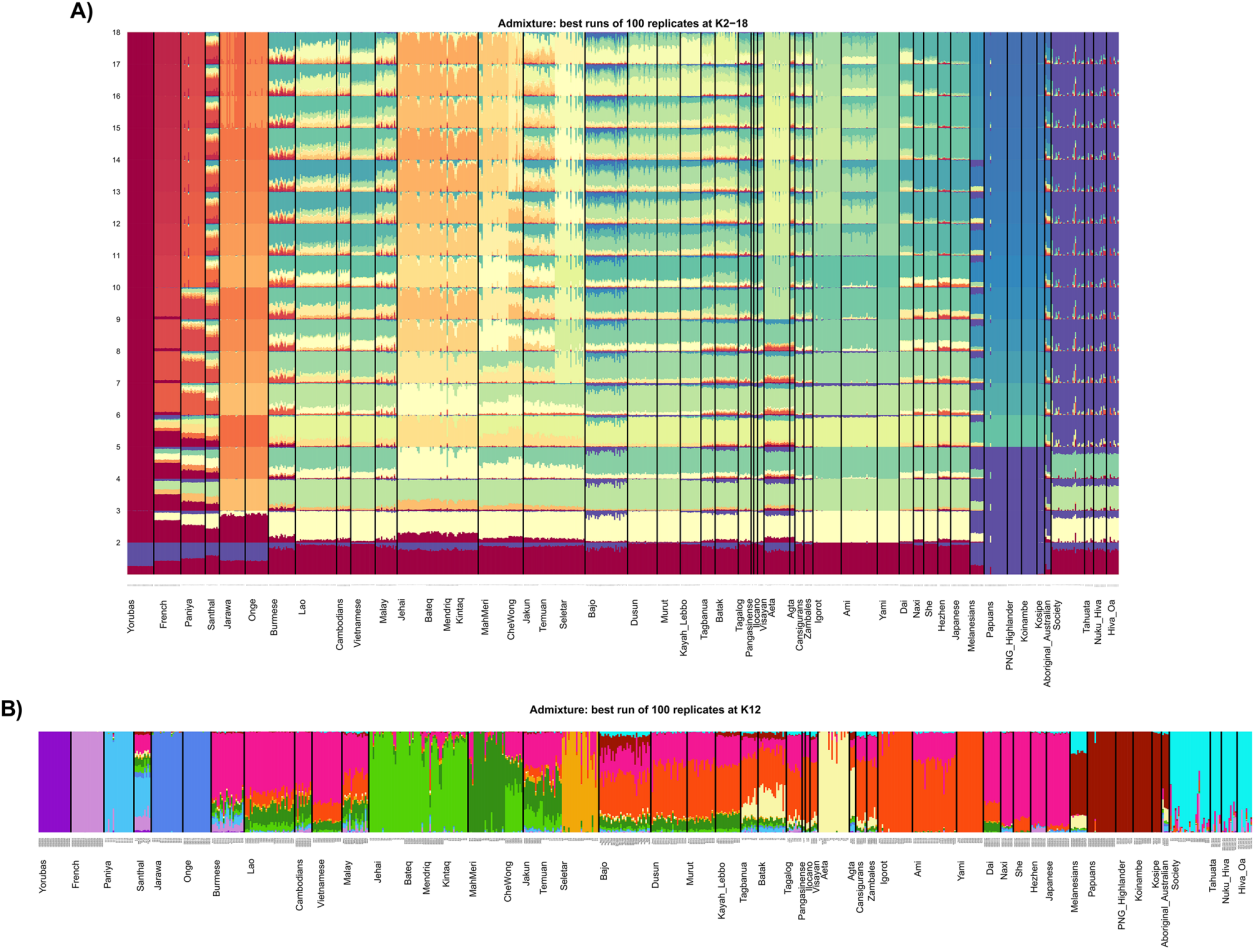
(**A**) Structure analysis based on autosomal SNP loci. Best runs of 100 replicates at K = 2–18. Please refer to Supplementary Table 1 for specific populations examined within each region. K values are indicated on the Y-axis. (**B**) Structure analysis of populations at K = 12. Please refer to Supplementary Table 1 for specific populations examined within each region.

The admixture analysis based on the autosomal SNP data indicates homogeneity in the genetic constitution among the islands of the Marquesas and Society Archipelagos examined at all the *K* values (*K* = 2–18) (Fig. 4A). The lowest cross-validation (CV) score of ADMIXTURE is observed at *K* = 12 (Supplementary Figs. 1 and 2). All the East Polynesians groups share a prominent component with ISEA/East Asia populations at *K* = 3–5 (light cream at *K* = 3, light green at *K* = 4 and aquamarine at *K* = 5). In some of the reference populations such as the Ami and Yami aborigines of Taiwan, the Igorot of the Philippines and the Dusun and Murut of Brunei this component represent the entire genome. This DNA element is about 80% of the Marquesas and Society DNAs. The other constituent (purple) representing approximately 20% of the genome of the Marquesas and Society genomes is likely of Papuan/Melanesian origin representing about 70–100% of their DNA. This purple element is also 80% of the Australian aboriginal genome. In addition to the characteristic predominant purple element of Papuans/Melanesians, the population from Melanesia also exhibits the ISEA/East Asia component (~ 30%). In addition, a number of Marquesas and Society individuals exhibit minor amounts of a red signal likely of European origins likely resulting from recent admixture. At higher *K* values (*K* = 5–18) all East Polynesian populations adopt a single deep blue component with some individuals exhibiting traces of Papuan/Melanesian (light blue), ISEA/East Asian (aquamarine and/or light cream) and /or European (red) ancestry. No additional elements are seen in the Marquesas and Society after *K* = 5. Greater resolution of components within the Structure analysis at K = 12 is provided in Fig. 4B. Figure 4B indicates the identity (designation number) of all the individuals used in the structure analysis.

### Admixture, genetic affinities and average heterozygosity

Based on the f3 analysis the Tahuata population exhibits signs of admixture. The negative f3 values for the Nuku Hiva—Hiva Oa and Society Islands—Nuku Hiva pairs suggest their genetic contribution to the Tahuata population (Supplementary Table 4). No other indication of genetic contribution from reference populations to the Marquesas groups studied was observed (Supplementary Tables 5 and 6). To assess if the Nuku Hiva, Hiva Oa and Tahuata always form a clade with respect to reference populations, f4 analysis was performed. When f4 analysis of the form “Nuku Hiva, Tahuata; French, Yoruba” was performed, Nuku Hiva and Tahuata shared a significant negative value with the French group suggesting their shared ancestry with the French (Supplementary Table 7). No excess ancestry is observed between the Marquesas populations and any of the other reference groups (Supplementary Tables 8 and 9). To further investigate the admixture signals observed in the results of the f3 and f4 tests, ALDER statistical analyses were performed (Supplementary Tables 10, 11 and 12). When ALDER was applied to Tahuata, the population that generated signals of admixture with the f3 and f4 tests, two categories of best fit (based on the highest LD decay curve amplitude values) of source populations were observed (Supplementary Table 10). One type and the best scores consisted of the French and a Southeast Asian population. These combinations involving the French reflected events that occurred 6.7–7.4 generations before the present (GBP) or 200–222 years before the present (YBP). In the second category, the best scores involved one source group from Papuan/New Guinea and the other from Southeast Asian. In most of these pairs, the Southeast Asian contributor is a population from the Philippines, such as Igorot, Zambales (from Luzon Island) or Taghanua (from Palawan, one of the southern islands of the Philippines). These admixture events are much older dating to 76.5–91.2 GBP or 2,295–2,735 YBP. For these more ancient admixture events, ALDER indicated "decay rates inconsistent", suggesting that the reference populations examined, signaling those admixture events, are not very similar to the actual ancient source populations. The results for Nuku Hiva are similar to Tahuata except that the Southeast Asian populations that pair with the French or the Papuan/New Guinea to generate the recent and older admixture events, respectively, often differ (Supplementary Table 11). For Hiva Oa only the older admixture events are detected (Supplementary Table 12).

The results of the Outgroup f3 analyses show that the most genetically similar population to Hiva Oa is the small nearby island of Tahuata (about 4 km away) (Supplementary Table 13). Other related populations in decreasing order of relatedness are the Society Islands, Nuku Hiva, Igorot, the Yami and Ami aborigines from Taiwan, and the Ilocano, Pangasinense and Casigurans from the island of Luzon in the Philippines. Nuku Hiva exhibits its greatest genetic affinity to the Marquesans from Tahuata and Hiva Oa followed by the Society Islands, the Igorots, the Yami and Ami aborigines of Taiwan, and the Ilocano, Pangasinense and the Casigurans from Luzon, Philippines (Supplementary Tasble 14). Tahuata’s genetically most similar populations are Hiva Oa, Nuku Hiva and the Society Islands groups, in that order, followed by the Igorots, Yami, Ami and the northern Filipino groups of Ilocano, Pangasinense and Casigurans (Supplementary Table 15).

The average heterozygosity of the Hiva Oa, Nuku Hiva and Tahuata groups is limited and comparable to the ones from the Society Islands of Bora Bora, Rai’atea, Taha’a and the Ami tribe of Taiwan while higher than the levels observed for Papua/New Guinea, Melanesians and the inbreed Yami aboriginal population of Taiwan (Supplementary Table 16).

### Haplotype-based analyses

**Figure 5:**
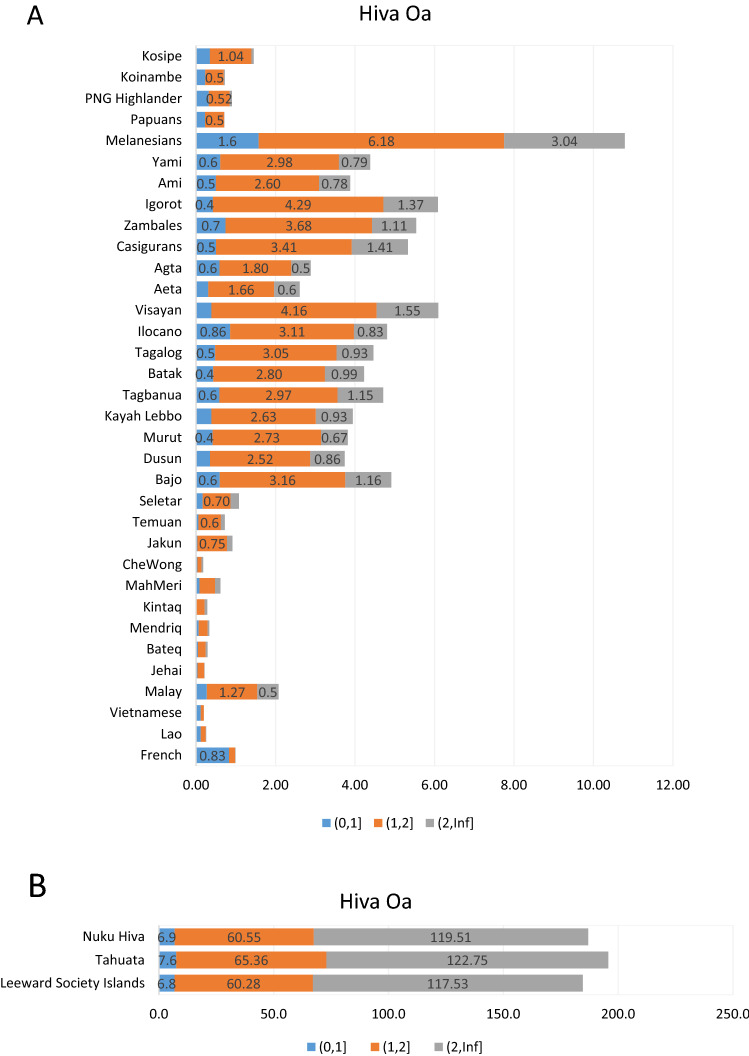
(**A**) Identical by descent tracts (IBDs) of populations relative to the Hiva Oa. (**B**) Identical by descent tracts (IBDs) of Marquesas and Society Islands populations relative to the Hiva Oa. Numbers on the horizontal bar graphs indicate the median IBD counts for the 0–1 cM, 1–2 cM and > 2 cM segments. Supplementary Table 23 provides the higher and lower confidence intervals at 95% for the 0–1 cM, 1–2 cM and > 2 cM ranges. Please refer to Supplementary Table 1 for specific populations examined.

**Figure 6:**
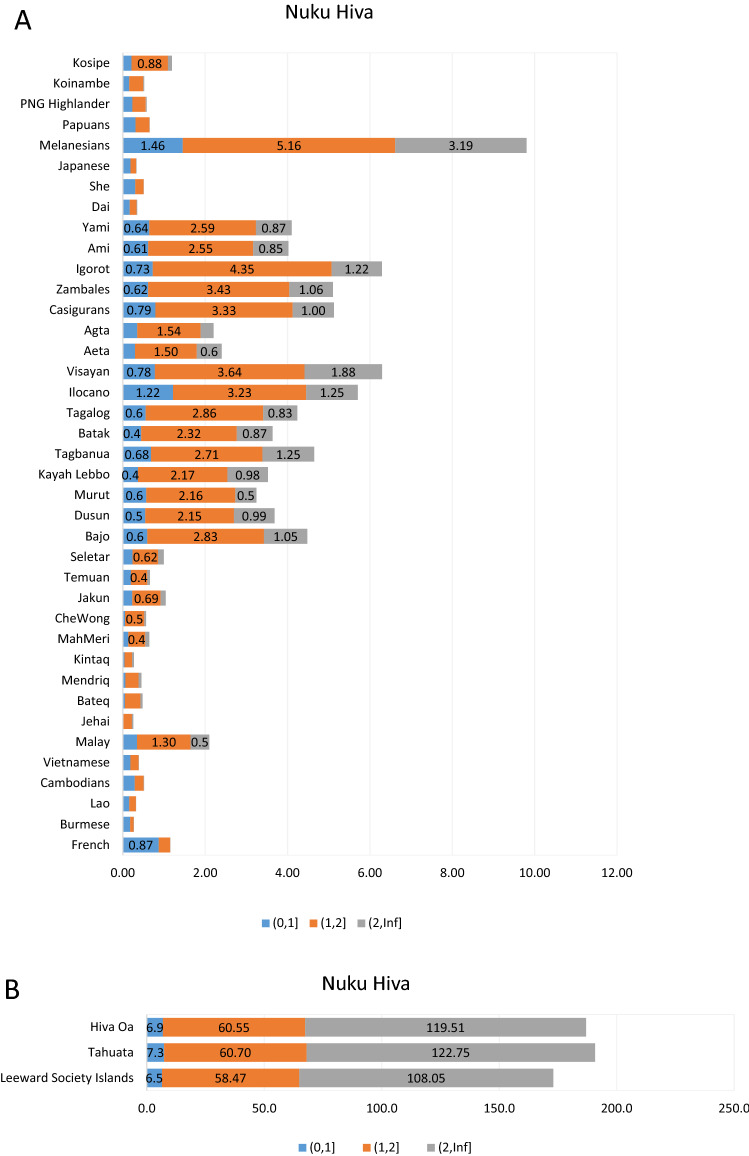
(**A**) Identical by descent tracts (IBDs) of populations relative to the Nuku Hiva. (**B**) Identical by descent tracts (IBDs) of Marquesas and Society Islands populations relative to the Nuku Hiva. Numbers on the horizontal bar graphs indicate the median IBD counts for the 0–1 cM, 1–2 cM and > 2 cM segments. Supplementary Table 24 provides the higher and lower confidence intervals at 95% for the 0–1 cM, 1–2 cM and > 2 cM ranges. Please refer to Supplementary Table 1 for specific populations examined.

**Figure 7:**
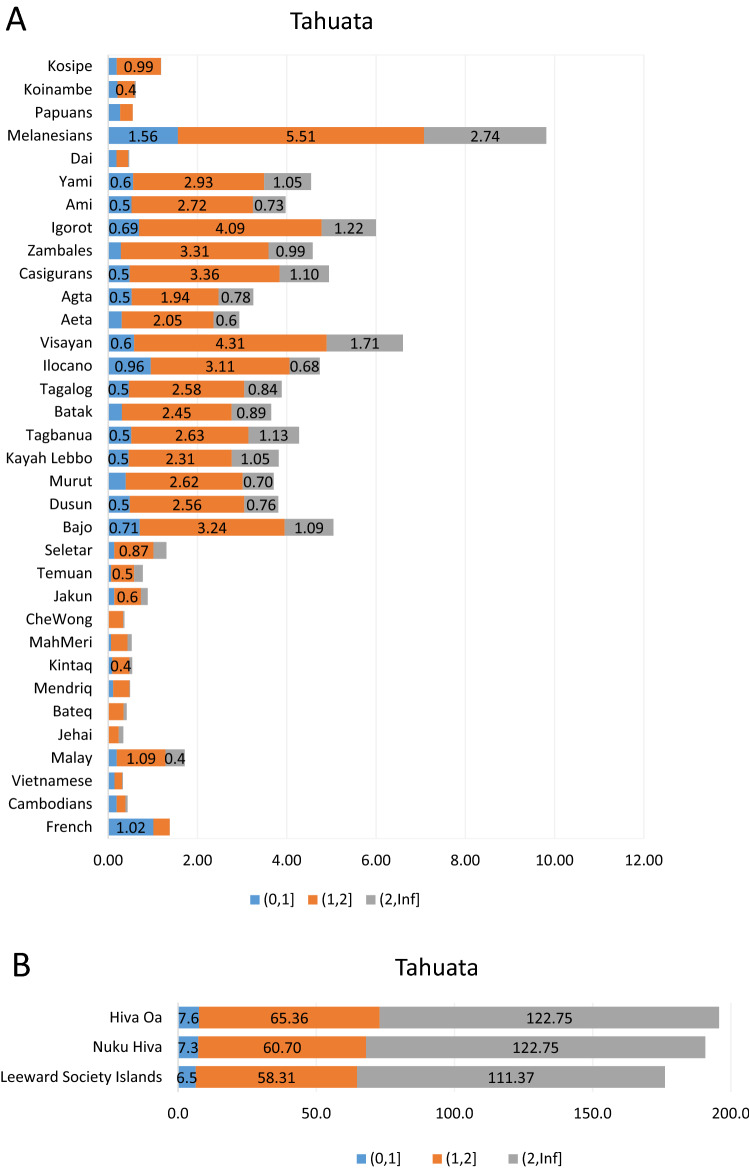
(**A**) Identical by descent tracts (IBDs) of populations relative to the Tahuata. (**B**) Identical by descent tracts (IBDs) of Marquesas and Society Islands populations relative to the Tahuata. Numbers on the horizontal bar graphs indicate the median IBD counts for the 0–1 cM, 1–2 cM and > 2 cM segments. Supplementary Table 25 provides the higher and lower confidence intervals at 95% for the 0–1 cM, 1–2 cM and > 2 cM ranges. Please refer to Supplementary Table 1 for specific populations examined.

The level of DNA sharing between the three Marquesas groups and a selected geographically targeted set of reference populations from MSEA, ISEA, Melanesia, Papua/New Guinea and Polynesia was examined. With this in mind, the median IBD (identical by descent) segment sharing between Hiva Oa (Fig. 5) Nuku Hiva (Fig. 6) and Tahuata (Fig. 7) with the reference populations were counted and graphed; 95% confidence intervals are provided in tabular form to evaluate statistical significance. In reference to Hiva Oa, the highest median IBD counts for DNA segments of three size classes are observed in relation to the Marquesas’ Nuku Hiva, Tahuata Islands and the Society Islands (0–1 cM [6.8–7.6 counts], 1–2 cM [60.3–65.4] and > 2 cM [117.5–122.8] (Fig. 5B). The rest of the reference populations rank into two general groups based on the number of median IBD counts. One category exhibiting moderate number of counts (0–1 cM [0.3–1.6 counts], 1–2 cM [1.7–6.20] and > 2 cM [0.5–3.0] is headed by the Melanesians followed by groups largely made up of Filipino populations (Fig. 5A). The other group of populations mainly from Indonesia and Papua/New Guinea exhibits modest counts (0–1 cM [0–0.1 except for the French with 0.8], 1–2 cM [0 to less than 1 except for the Malay with 1.3] and > 2 cM [0–0.5]). The other two Marquesas populations of Nuku Hiva and Tahuata show the same general pattern of IBD count distribution relative to the reference groups. They exhibit the same three categories of IBD counts except for fluctuations in the specific values (IBD counts) for individual reference populations and the failure to register of some reference populations from the third category with minimal number of counts.

### Uniparental markers

#### mtDNA

Complete mtDNA sequencing of four Nuku Hiva, three Hiva Oa and one Tahuata individuals were performed (Supplementary Table 17). All the mitogenomes belong to sub-haplogroup B4a1a1 typical of Polynesian- speaking populations. B4a1a1 derives from sub-haplogroup B4a1a, which all the Marquesas and Society samples shared with two Yami individuals from Orchid Island, Taiwan (Tätte et al., 2021) (Supplementary Fig. 5). Two individuals (from Nuku Hiva and Tahuata) belong to sub-haplogroup B4a1a1c and possess identical sequences. A third person (from Hiva Oa) shares the same B4a1a1c subhaplogroup but differ by one point mutation from the previous two. One individual from Hiva Oa and one from Nuku Hiva share sub-haplogroup B4a1a1m1 but differ by one point mutation from each other. The other singleton sub-haplogroups found in the Marquesas are B4a1a1, B4a1a1a and B4a1a1 + 16,126 (Supplementary Table 17 Supplementary Fig. 5). Our research group previously detected all the sub-haplogroups observed in the Marquesas in an early study of the Society Archipelago^4^. Furthermore, B4a1a1c and B4a1a1m1, the most frequently detected sub-haplogroups in the Marquesas are also the most abundant in the Society populations of Bora Bora, Rai’atea, Taha’a^4^.

#### Y- SNP

Supplementary Table 18 provides the Y-SNP genotypes and assigned haplogroups for every studied individual of the Marquesas Islands of Nuku Hiva, Hiva Oa and Tahuata. Supplementary Fig. 6 illustrates the phylogeny of the relevant Y chromosomal haplogroups and the position of the markers that were genotyped on the branches of the phylogeny. A phylogenetic tree and haplogroup diversity values are provided in Supplementary Fig. 6.

The most abundant Y chromosomal haplogroup in the Marquesas Islands of Nuku Hiva, Hiva Oa and Tahuata is C2a-M208 (37.9%, n = 33) (Supplementary Fig. 6) of Melanesian origin^17^. Although C2a-M208 predominates among the populations of the three islands examined, the frequency exhibited in each island varies: 43.1% in Nuku Hiva, 37.5% in Tahuata and 28.6% in Hiva Oa. The frequency of C-M130 individuals differed among the islands as well: 37.5% in Tahuata, 13.7% in Nuku Hiva and 10.7% in Hiva Oa. Next most abundant male lineage is haplogroup O of Asian origin^17^. The frequency of the O sub-haplogroups differs among the Marquesas Islands examined. Marquesans belonged to three O sub-lineages and some were found only in specific islands: O3-M188 (Hiva Oa, 3.6%; Tahuata, 12.5%), O3a’1-P164 (Nuku Hiva, 11.8%) and O3′6-M324 (Nuku Hiva, 2.0%; Hiva Oa, 3.6%). In addition, other haplogroups such as R1b1′12-M269 (European origin), G-M201 (European origin), I-M170/M258 (European origin), K-M9 (Melanesian origin) and Q-M242 (Asian origin) were detected mostly at minor frequencies and only in specific islands: R1b1′12-M269, 3.9% in Nuku Hiva and 3.6% in Hiva Oa; G-M201 7.1% in Hiva Oa; I-M170/M258, 13.7% in Nuku Hiva; K-M9, 28.6% in Hiva Oa and Q-M242, 9.8% in Nuku Hiva. This uneven distribution of markers from different sources suggests that complex differential patterns of migrations likely involving drift and/or founder effects impacted the islands of Nuku Hiva, Hiva Oa and Tahiti.

#### Y- STR

Supplementary Table 19 provides the 27-loci Y-STR haploypes for every individual of the Marquesas Islands of Nuku Hiva, Hiva Oa and Tahuata. Rst distances were estimated (Supplementary Table 20) using the Y-STR genotypes of the three Marquesas Islands and geographically targeted reference populations from MSEA, ISEA, Melanesia and Polynesia (Supplementary Table 1) and MDS plots were constructed based on the Rst distances (Supplementary Figs. 7, 8 and 9). The MDS projection in Supplementary Fig. 7 partitions the aboriginal groups of Taiwan in quadrants I and IV. The center of the plot exhibits populations from East Asia, South East Asia, Near Oceania and the Philippines while quadrant II partitions the populations from Bora Bora and Rai’atea of the Society Archipelago and Nuku-Hiva and Hiva-Oa of the Marquesas Archipelago. In proximity to the East Polynesian groups there are a number of populations from the Solomon Islands (Onttong and Rennell), East Asian and Madagascar. For greater resolution of the conglomerate of groups in the center of the plot, Supplementary Fig. 8 provides an expansion of the populations. When the Bora Bora, Rai’atea, Nuku-Hiva and Hiva-Oa groups were examined in relation to Taiwanese aboriginal tribes, the expected segregation of East Polynesian populations according to archipelagos (i.e., Marquesas versus Society) is observed (Supplementary Fig. 9).

**Figure 8:**
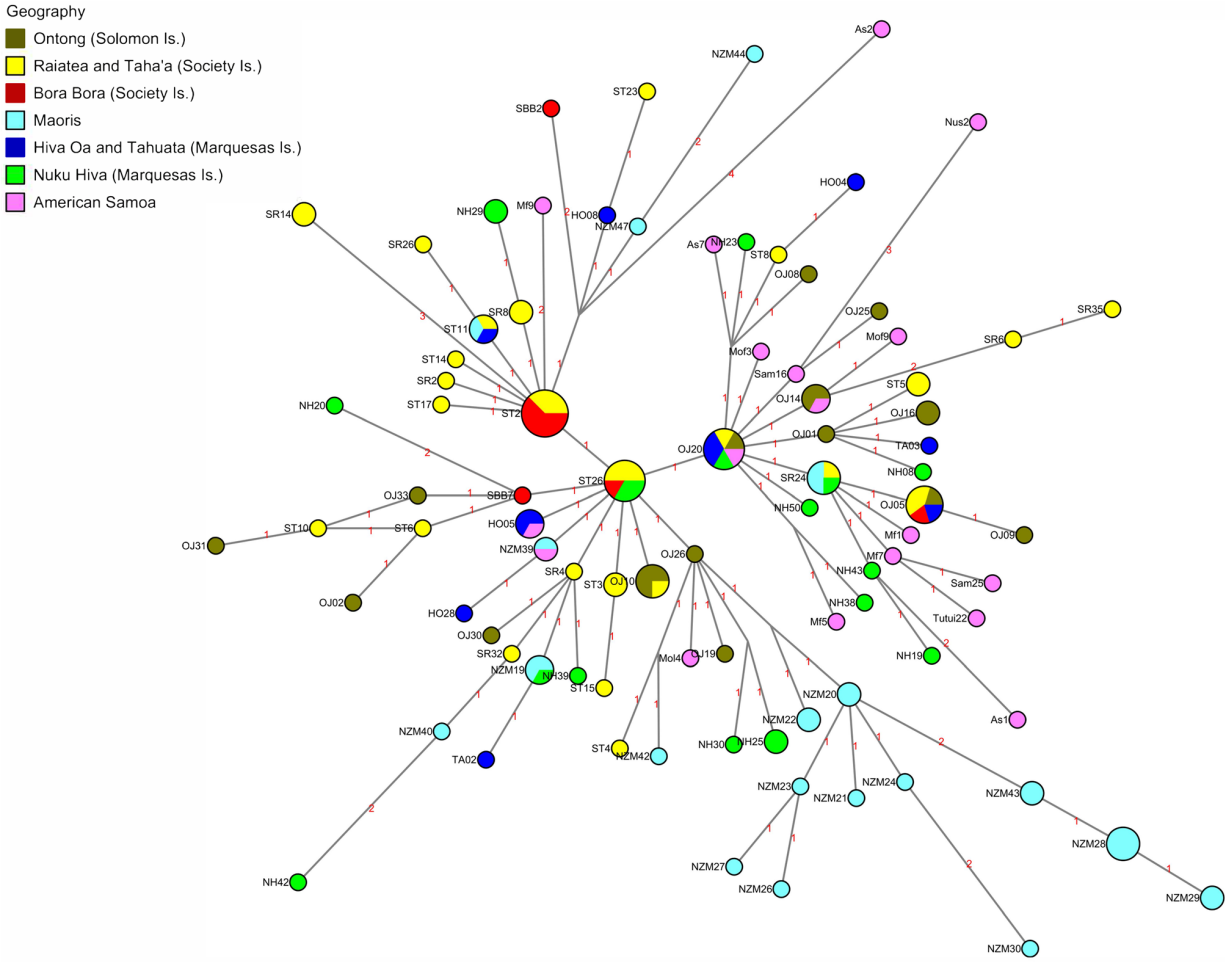
Median Network analysis based on Y-STRs under lineage C2a-M208. Color key indicates the populations examined. Size of nodes are proportional to the number of individuals sharing haplotypes. Mutations events are indicated next to the lines connecting nodes.

To further investigate the relationships seen between the East Polynesian and the Oceanic populations seen in the Y-STR MDS plot and considering that C2a-M208 is the most abundant Y-chromosome haplogroup in West and East Polynesian, we performed a Median Network analysis (Fig. 8) based on high resolution Y-STR data under the haplogroup (Supplementary Table 21). Figure 8 shows the mutational steps as well as the designation of the samples represented by each node. The populations employed in this Median Network analysis were Hiva Oa and Nuku Hiva from the Marquesas Archipelago, Rai’atea and Bora Bora from the Society Archipelago, American Samoa, Maoris, and Ontong from the Solomon Archipelago. In addition age estimations were generated (Supplementary Table 22). The Median Network exhibits a star-like topology made up of one central and two secondary major nodes from which individuals from different populations radiate-out in multiple lineages to generate the network. Individuals from East Polynesia, especially Rai’atea and Bora Bora (Society), predominate in these three central nodes. Throughout the Network, haplotype-sharing is seen in a number of multi-population star-like nodes from which individuals from different island groups branch-out. Away from the central major nodes towards the periphery of the Network extensive interconnections among individuals from all the islands examined is observed. No intra- or inter-population substructure is seen with the exception of the Maoris in which most of its individuals segregate distinctly into one specific sequential lineage. Also, individuals from the Polynesian outlier population of Ontong in the Solomon Archipelago shares a number of haplotypes or derive from East Polynesian ancestors (especially from Rai’atea) and several occupy peripheral positions.

## Discussion

Based on archaeological and genetic evidence, the time line of the settlement of Polynesia by Austronesians indicates that the Tonga Archipelago was populated around 3300 ya and the Samoan Islands 300 years later. Radio-carbon dating suggests that the settlement of East Polynesia occurred about 1020 ya. From the Society Islands, the Marquesas were populated approximately 830–730 ya, Rapa Nui 820 ya, Hawaii 800–850 ya and New Zealand 740 ya^17^. The Marquesas Islands represent one of the eastern-most locations reached by Austronesians and as such provide a case study for the analysis of the effects of a protracted process of step-wise migration by small number of people. Although it is clear that the origins of Polynesians is in Asia, likely with strong roots in Taiwan and contributions from Melanesians, recent findings suggest that the settlement of Oceania was not a simple sequential and unidirectional dispersal^4^. In recent years it has become apparent that subsequent to the original settlement, Polynesians traveled among near and far islands trading goods and supplies. Since individual islands only have limited resources and each island provided only certain raw materials, the inhabitants of the outposts did not see the ocean as a barrier but more like a liquid highway that allowed them to slide in their outrigger or double-hulled canoes communicating and trading for necessary goods. Therefore, although it is not clear the reasons and motivations for setting out to sea without a specific destination, not knowing whether they would perish in the open ocean prior to finding land, Polynesians traveled thousands of kilometers in their voyages of discovery and then continued crisscrossing the open ocean resettling and trading.

Although we may never know the reasons why Austronesians set out to sea in the first place, archeological and genetic markers are continuously providing detail information on the movements of Polynesians within Oceania including the time line of their treks. With this in mind, this article reports on a series of experiments based on high-resolution biparental and uniparental DNA markers that attempt to shed light on the settlement and population dynamics of the inhabitants of the Marquesas Archipelago at the fringes of the Austronesian expansion.

The populations of the Marquesas examined in this study are overall genetically homogeneous. In the Marquesan Archipelago all of the mtDNA samples sequenced are of Asian origin belonging to the B4a1a1 subhaplogroup as the region marks the end of a west to east decreasing cline of Melanesian mtDNA starting with West Polynesian populations. This genetic homogeneity is also reflected in the limited average heterozygosity relative to most of the reference populations. The uniformity of constituents in the Structure analyses and complete mtDNA sequences indicate that the three Marquesas Islands examined are genetically extremely homogeneous. Of the eight complete mitogenomes sequenced two subhapogroups (B4a1a1m1 and B4a1a1c) are represented by two individuals each. The limited genetic diversity that is seen in the populations of Hiva Oa, Nuku Hiva and Tahuata likely derives from repeated bottleneck episodes, genetic drift, founder effects and inbreeding since the settlement of the islands.

One widespread theme that emerges from most of the results generated in this study is the genetic uniformity exhibited by the Marquesas and Society Islands examined. Genetic data from these two archipelagos indicate a certain degree of homogeneity within East Polynesia. Examination of the autosomal SNP data in the form of the PC1/PC2 principal component analysis indicates a compact cluster made up of the Ami and Yami Taiwanese aboriginal populations and the Igorot indigenous group from the northern Filipino island of Luzon in the lower left-hand corner of the plot (Fig. 2). Immediately following this threesome cluster there are the populations from the Philippines, MSEA and Indonesia, in that order. This partitioning of populations mirrors the putative relationship and migration route of Austronesians during their expansion into Oceania starting in the island of Taiwan^10^. Except for one individual from Bora Bora that segregates with the Bajo of Indonesia, most of the samples from the Marquesas and Society Islands plot into a distinctly separate and tight cluster with a number of them radiating out towards a Indonesian-Filipino conglomerate. Nevertheless a gap is evident between the East Polynesians and the Filipino-Indonesian cluster. This topological relationship suggests a level of phylogenic discontinuity between the East Polynesians and the ISEA populations as well as a degree of isolation of the former. The limited intermingling of individuals from the Marquesas and Society Islands within this tight clutch also indicates genetic homogeneity and lack of sub-population structure among them. The age estimations of the most abundant C2a-M208 lineage of the Y-chromosome in the Marquesas and Society populations examined are recent and comparable (Supplementary Table 22) and it is likely that equivalent number of individuals from similar sources colonized them. Considering that these two groups of islands are about 1,500 km apart, it seems that communication existed between them to maintain the observe homogeneity among them. A similar partitioning of the same populations and tight cluster of East Polynesians is observed when a principal component analysis was performed along the PC1/PC3 axis except that this time three individuals from the Society (two from Bora Bora and one from Taha’a) segregated in the proximity of a different grouping of Indonesians (Supplementary Fig. 3). Principal component analyses conducted at higher resolution (Fig. 3 and Supplementary Fig. 4) using only Marquesas and Society individuals were able to segregate, although still in close proximity, most of the samples from the two archipelagos, especially along the PC1/PC2 components. In these higher-resolution PC analyses a number of individuals (SBB 3, ST 1, SBB 1, HO 16, NH 48 and NH 29) partition away from the main cluster of East Polynesians. When these individuals are examined in the Structure analysis (Fig. 4B), it is seen that they represent admixed persons of French (light purple component) and/or Asian (orange/pink components) ancestry.

Based on autosomal SNP data, the Structure analyses also demonstrate uniformity within East Polynesia (Fig. 4A,B). The Structure analyses paint a picture of limited genetic variability and homogeneity among the East Polynesian Islands of the Marquesas and Society. Except for the admixed individuals with European ancestry, genetic uniformity is the hallmark of both archipelagos. These characteristics are likely the result of repeated bottleneck and inbreeding couple with frequent communication and gene flow between the two archipelagos.

Similarly, the mean IBD counts estimated from SNP markers illustrate comparable values among the Marquesas and Society Islands examined indicating overall genomic homogeneity among them (Figs. 5, 6, 7). Next in IBD values to the East Polynesian populations (Figs. 5B, 6B, 7B) is the Melanesian population, which exceeds in all three-size categories (0–1 cM, 1–2 cM and > 2 cM) the groups from the Philippines and Indonesia, in that order (see confidence intervals in Supplementary Tables 23–25). These significantly higher IBD values exhibited by the Melanesians relative to Hiva Oa, Nuku Hiva and Tahuata suggest greater contribution of this group to the migrating Austronesians compared to the original inhabitants of the Philippine and Indonesian Archipelagos. Also, the differences observed in the magnitude of IBD values among the Marquesas and Society Islands compared to the IBD levels between East Polynesians to the rest of the reference populations from Oceania parallels the gap in the partition and separation of these groups of populations observed in the PC1/PC2 (Fig. 2) and PC1/PC3 (Supplementary Fig. 3) pointing to the relative isolation of East Polynesia from other Pacific groups.

Based on the f3 analysis the Tahuata population exhibits signs of admixture. The negative f3 values for the Nuku Hiva—Hiva Oa and Society Islands—Nuku Hiva pairs suggest their genetic contribution to the Tahuata population (Supplementary Table 4). Furthermore, the Outgroup f3 results show that the groups that exhibit the most genetic affinities to each other are the Marquesas and Society populations (Supplementary Tables 13–15). Both the f3 and the Outgroup f3 results provide indications of intra-East Polynesian gene flow and corroborate the results obtain in the principal component, sub-population structure and IBD results previously discussed, which suggest homogeneity within East Polynesia.

To further assess if the Hiva Oa, Nuku Hiva and/or Tahuata always form a clade with respect to reference populations, f4 analysis was performed (Supplementary Tables 7–9). Only Tahuata shared a significant negative value with the French group suggesting their shared ancestry with the French or other European group, likely recently. No other excess ancestry is observed between the Marquesas populations and any of the other reference groups. Similarly, this data also support the notion that since the original settlement of East Polynesia, gene flow from Papuan/Asian origins has been limited. To further substantiate the gene flow results provided by the f3 and f4 analyses, ALDER statistical analyses were performed (Supplementary Tables 10–12). ALDER uncovered two categories of source populations for Tahuata, one involved the French in combination with a Southeast Asian population reflecting admixture event(s) dating to about 6.7–7.4 generations before the present (GBP) or 200–222 years before the present (YBP). This admixture event corresponds roughly with the date East Polynesia became a protectorate of France. The other category included a Papuan/New Guinea population with a Southeast Asian group as ancestors dating to around 76.5–91.2 GBP or 2,295–2,735 YBP. The dates provided by ALDER and the source populations involved are congruent with the known approximate dates for the European incursion into East Polynesia and the original settlement of the islands by Austronesians^17^. ALDER does not detect gene flow during the time period in between these two admixture events suggesting a period of relative isolation. For these more ancient admixture events, ALDER indicates "decay rates inconsistent" (Supplementary Table 10), suggesting that the reference populations for those admixture events are not very similar to the actual ancestral populations, an expected result considering that in these analyses we are using contemporary populations that have changed since the admixture event(s). The results for Hiva Oa and Nuku Hiva are similar to Tahuata except that for Hiva Oa only the older admixture event(s) are detected (Supplementary Tables 11 and 12) suggesting more limited admixture from Europeans in Hiva Oa.

The segregation of MSEA, ISEA and Oceanic populations based on Y-STR loci provided in Supplementary Fig. 7 generally illustrates a west to east geographical progression of populations with Taiwanese aborigines at one end of a continuum in quadrant I followed, in order, by Filipino, SE Asian, Near Oceania and East Polynesian populations at the other end in quadrant II. Yet, the Ontong population from the Solomon Archipelago partitions close to the East Polynesians, especially to the Hiva Oa and Nuku Hiva groups of the Marquesas. Previous phylogenetic work has suggested that the origin of the East Polynesians is in islands of central northern outlier Polynesia such as Ontong, rather than Samoa in West Polynesia^4^. Since the C2a-M208 is the most abundant Y-chromosome lineage in Ontong and Polynesian^4^ populations, including the Marquesas (Supplementary Fig. 6), we decided to explore further the relationships of haplotypes by Median Network analysis and age estimations of C2a-M208 in key Oceanic populations in relation to the East Polynesian groups. Of the populations examined in the Median Network (Fig. 8), the Marquesas Archipelago are approximately 5,870 km northeast from New Zealand (Maoris), 3,630 km east from Samoa, 1,570 km northeast from the Society Archipelago and 6,830 km east from Ontong (Ontong is about 3, 200 km west of Samoa). The star-like Network based on high-resolution Y-STR loci exhibits three central nodes dominated by populations from East Polynesia, especially from the Society Archipelago. From these three, for the most part East Polynesia-specific nodes, the rest of the populations analyzed radiate out one mutational step away. Singleton and doubleton haplotypes emanate towards the periphery of the Network, for the most part one-mutation step from each other. The Maoris and Samoans often times occupy peripheral positions stemming from these central East Polynesia-specific nodes. The random distribution of the various populations starting from an East Polynesia stem and the peripheral location of many haplotypes from Samoa in West Polynesia and Ontong Java are more compatible with migrations of the C2a-M208 Y chromosome lineage from East Polynesia rather than from Samoa West Polynesia or Ontong Java to East Polynesia. This observation and the limited intra- and inter-population substructure seen in the topology of the Network is also congruent with substantial dispersals of C2a-M208 Y chromosomes stemming from East Polynesia signaling migrations in various directions westward instead of eastward as traditionally envision.

The age estimations of the C2a-M208 lineage for the populations in the Network analysis provide equivalent values except for the Maoris of New Zealand, which exhibit values approximately twice or more than the other groups (Supplementary Table 22). The age of C2a-M208 based on Y-STR variability data for the Maori is incompatible with the radiocarbon dating of archaeological sites, which indicate that Polynesians settled New Zealand by about 740 ya^45^. Data indicating rapid spread of populations over 12,000 km of coastline and high diversity in the mtDNA of first generation settlers suggest that New Zealand was the target of a planned mass migration out of East Polynesia during the first decades of the fourteenth century^46^. It is likely that such colonization by large number of individuals may have carry high levels of genetic variability within the Y-chromosomes reflected in the high diversity levels of C2a-M208 chromosomes in the Maori population relative to the other Polynesian and Polynesian outlier groups examined.

Congruent with the topology of the Network, the age estimations indicate that the C2a-M208 lineage is not older in Ontong compared to East Polynesia, not providing support for a west to east gene flow from the former. Furthermore, as with the Network analysis, the equivalent ages of C2a-M208 in East and West Polynesia as well as in the Polynesia outlier Ontong suggest gene flow among the populations, possibly East Polynesia as a migrational hub. Due to the limitations of Y-STR markers for assessing age, which include uncertain mutation rates and methodological approaches, the dates provided in this study should only be used for comparisons of relative ages among the populations examined in this study.

## Conclusions

Genetic examination of biparental and uniparental markers of the populations of the islands of Nuku Hiva, Hiva Oa and Tahuata of the Marquesas Archipelago reveal widespread homogeneity and affinity with the Society Archipelago as well as isolation. Genetic analyses reveal limited average heterozygosity, uniformity of constituents in the Structure analyses, limited mtDNA diversity and affinity with Asian and other Oceanic populations as well as intra-East Polynesian differentiation in the PCAs and large number of IBD segments in common among Marquesas and Society populations. F3 and the Outgroup f3 admixture tests indicate intra-East Polynesian gene flow while ALDER analyses point to two gene flow episodes, one that coincides roughly with the European incursions into the islands and an early one that may represent the original Austronesian settlement. Median Network analysis and age estimations based on high-resolution Y-STR loci under the C2a-M208 lineage suggests east to west dispersals of East Polynesian (especially from the Society Archipelago) individuals with this sub-haplogroup.

## Supplementary Information

Supplementary Legends.

Supplementary Figure 1.

Supplementary Figure 2.

Supplementary Figure 3.

Supplementary Figure 4.

Supplementary Figure 5.

Supplementary Figure 6.

Supplementary Figure 7.

Supplementary Figure 8.

Supplementary Figure 9.

Supplementary Table 1.

Supplementary Table 2.

Supplementary Table 3.

Supplementary Table 4.

Supplementary Table 5.

Supplementary Table 6.

Supplementary Table 7.

Supplementary Table 8.

Supplementary Table 9.

Supplementary Table 10.

Supplementary Table 11.

Supplementary Table 12.

Supplementary Table 13.

Supplementary Table 14.

Supplementary Table 15.

Supplementary Table 16.

Supplementary Table 17.

Supplementary Table 18.

Supplementary Table 19.

Supplementary Table 20.

Supplementary Table 21.

Supplementary Table 22.

Supplementary Table 23.

Supplementary Table 24.

Supplementary Table 25.
