# Supplementary material for: Genetic characterization of populations in the Marquesas Archipelago in the context of the Austronesian expansion
Source: Sci Rep. 2022 Mar 29;12:5312. doi: 10.1038/s41598-022-08910-w (PMC8964752; doi:10.1038/s41598-022-08910-w)
Supplement: Supplementary file 1 — Supplementary Legends. [file 41598_2022_8910_MOESM1_ESM.docx]

**Supplementary Figure and Table Legends**

**Supplementary Figures:**

Supplementary Figure 1. Cross-validation indexes for K=2-18. X-axis indicates K values 2 to 18.

Supplementary Figurev 2. Cross-validation indexes for K=10-14. X-axis indicates K values 10 to 14.

Supplementary Figure 3. PC1/PC3 plot of East Polunesians and reference populations based on autosomal SNP loci. Color key to the populations examined is provided in Supplementary Table 3. Acronyms indicating populations are indicated adjacent to the individuals in the plot. Please refer to Supplementary Table 1 for specific populations examined within each region. ST = Society Taha’a,  SR = Society Rai’atea, SBB = Society Bora Bora, NH =Nuku Hiva, HO = Hiva Oa, TA = Tahuata.

Supplementary Figure 4. PC1/PC3 plot of East Polynesian populations. Designations for each individual are indicated adjacent to the individuals in the plot. ST = Society Taha’a,  SR = Society Rai’atea, SBB = Society Bora Bora, NH =Nuku Hiva, HO = Hiva Oa, TA = Tahuata.

Supplementary Figure 5. Phylogenetic tree based on complete mtDNA sequences of suprahaplogroup R in the Marquesas, Society, Ami and Yami of Taiwan.

Supplementary Figure 6. Y choromomal haplogroup frequencies and diversity values in the Marquesa Islands of Hiva Oa, Nuku Hiva and Tahuata Islands.

Supplementary Figure 7. MDS plot of Marquesas and Society Island populations in the context of reference populations based on Rst values. Color key to geographical regions is provided and Supplementary Table 1 indicates specific populations examined. Acronyms indicating populations are indicated adjacent to the populations in the plot.

Supplementary Figure 8. Expansion of MDS central region in Supplementary Figure 7.

Supplementary Figure 9. MDS plot of Marquesas and Society Island populations in the context of the Ami and Yami reference populations of Taiwan and South East Asian populations based on Rst values. Supplementary Table 1 indicates specific populations examined. Acronyms indicating populations are indicated adjacent to the populations in the plot.

**Supplementary Tables:**

Supplementary Table 1. Populations Examined.

Supplementary Table 2. New Y-SNP Marquesas primer specifications.

Supplementary Table 3. Color key of populations.

Supplementary Table 4 Tahuata F3 admixture analysis

Supplementary Table 5. Nuku Hiva F3 admixture analysis

Supplementary Table 6. Hiva Oa F3 admixture analysis

Supplementary Table 7. Nuku Hiva-Tahuata f4 grouping analysis

Supplementary Table 8 Hiva Oa-Nuku Hiva f4 grouping analysis

Supplementary Table 9. Hiva Oa-Tahuata f4 grouping analysis

Supplementary Table 10. Tahuata_ALDER analysis

Supplementary Table 11. Nuku Hiva ALDER analysis

Supplementary Table 12. Hiva Oa ALDER analysis

Supplementary Table 13. Hiva Oa outgroup analysis

Supplementary Table 14. Nuku Hiva outgroup analysis

Supplementary Table 15. Tahuata outgroup analysis

Supplementary Table 16. Average heterozygosity values

Supplementary Table 17. mtDNA complete sequences

Supplementary Table 18. Marquesas Y genotypes

Supplementary Table 19. Marquesas Y STR genotypes

Supplementary Table 20. Rst values of populations in MDS plots

Supplementary Table 21. Haplotypes based on 15-STR loci under C2a-208 lineage of the Marquesas and reference populations

Supplementary Table 22. C2a-M208 Age estimates in the Marquesas and reference populations
