# Supplementary figures and images for: Genetic characterization of populations in the Marquesas Archipelago in the context of the Austronesian expansion
Source: Sci Rep. 2022 Mar 29;12:5312. doi: 10.1038/s41598-022-08910-w (PMC8964752; doi:10.1038/s41598-022-08910-w)

# CV index @ all Ks

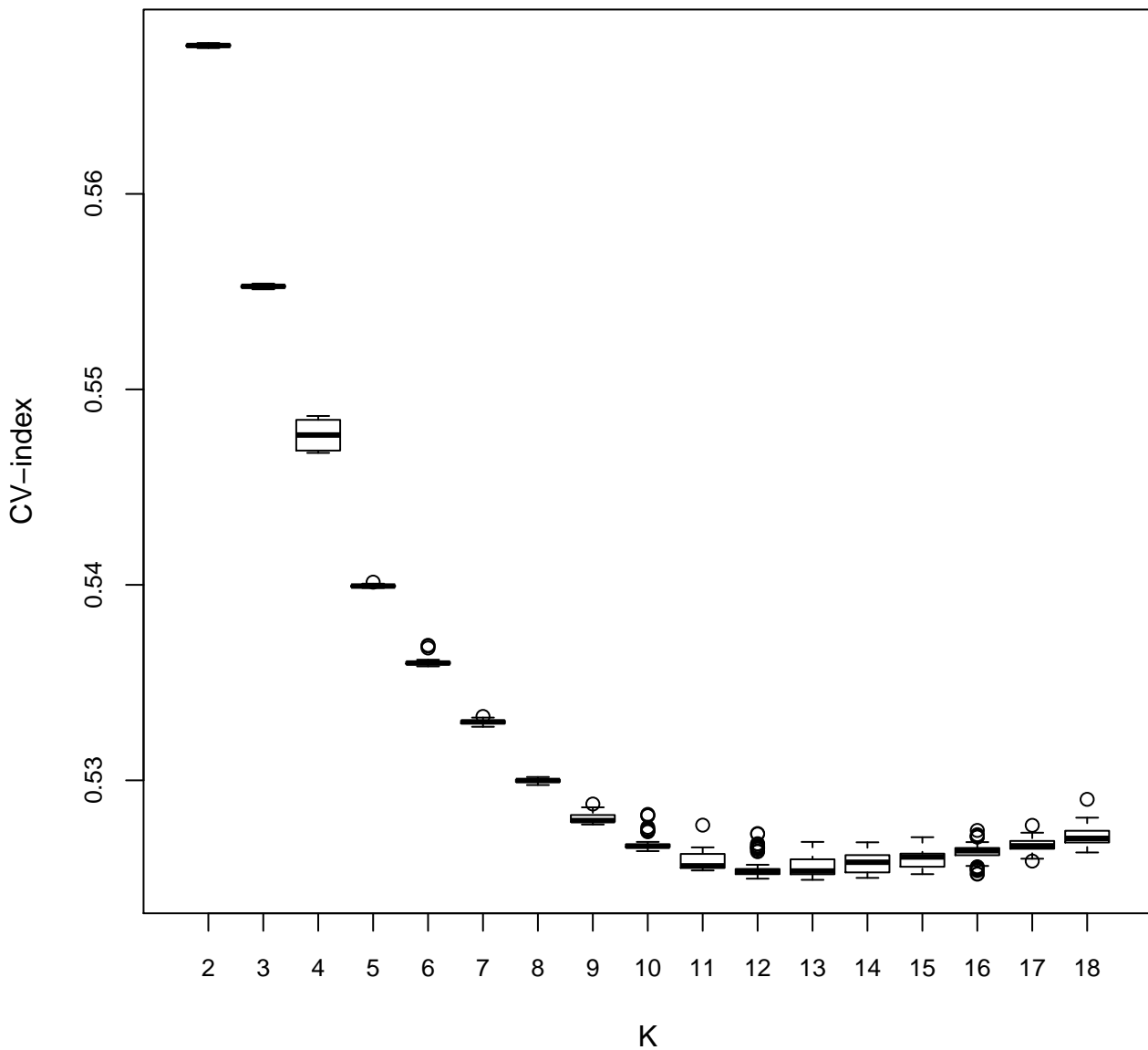

Supplement: Supplementary file 2 — Supplementary Figure 1. [file 41598_2022_8910_MOESM2_ESM.pdf]

CV index, from K=10 to K=14

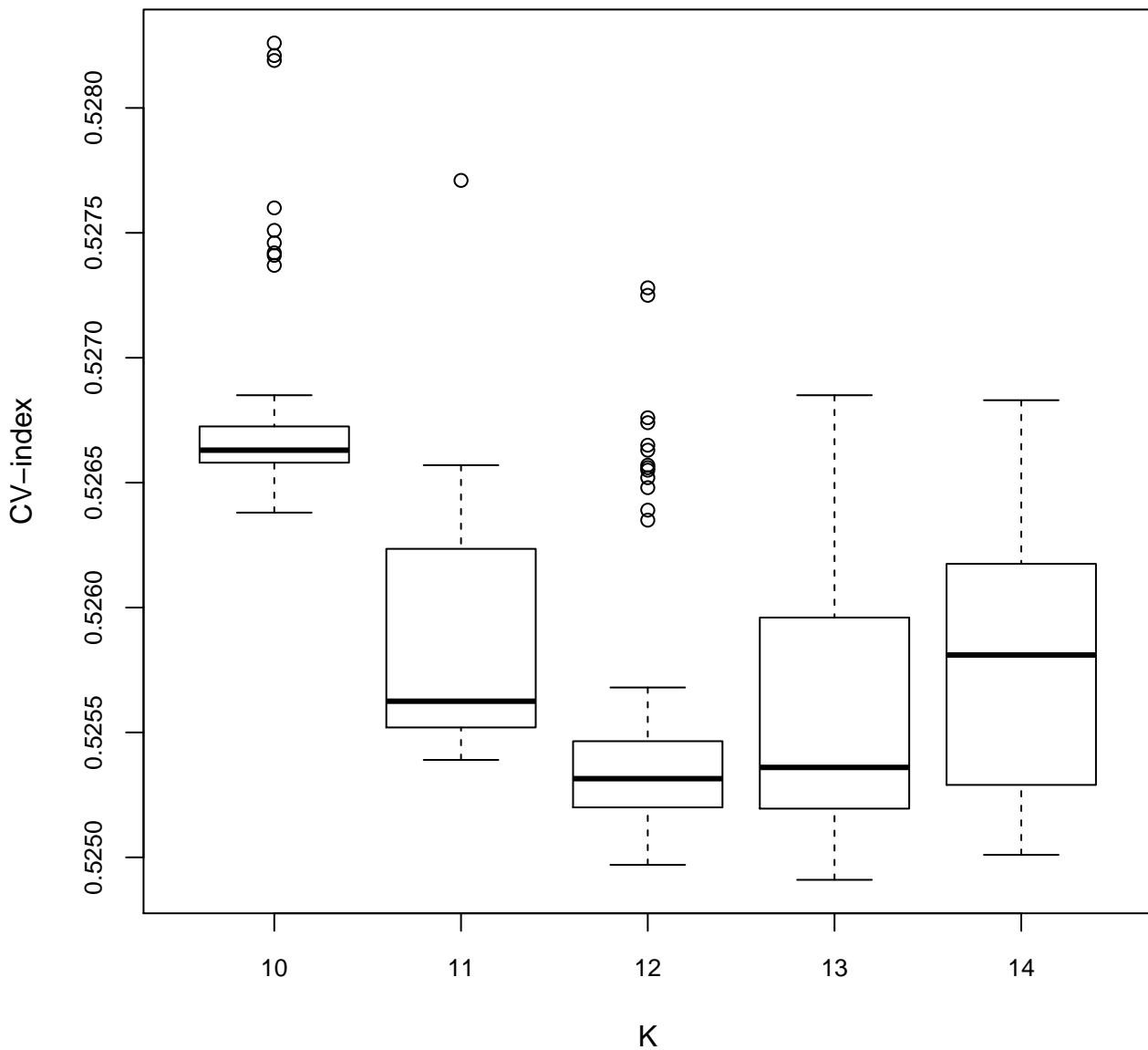

Supplement: Supplementary file 3 — Supplementary Figure 2. [file 41598_2022_8910_MOESM3_ESM.pdf]

Supplementary Figure 3. PC1/PC3 of East Polynesians and reference populations.

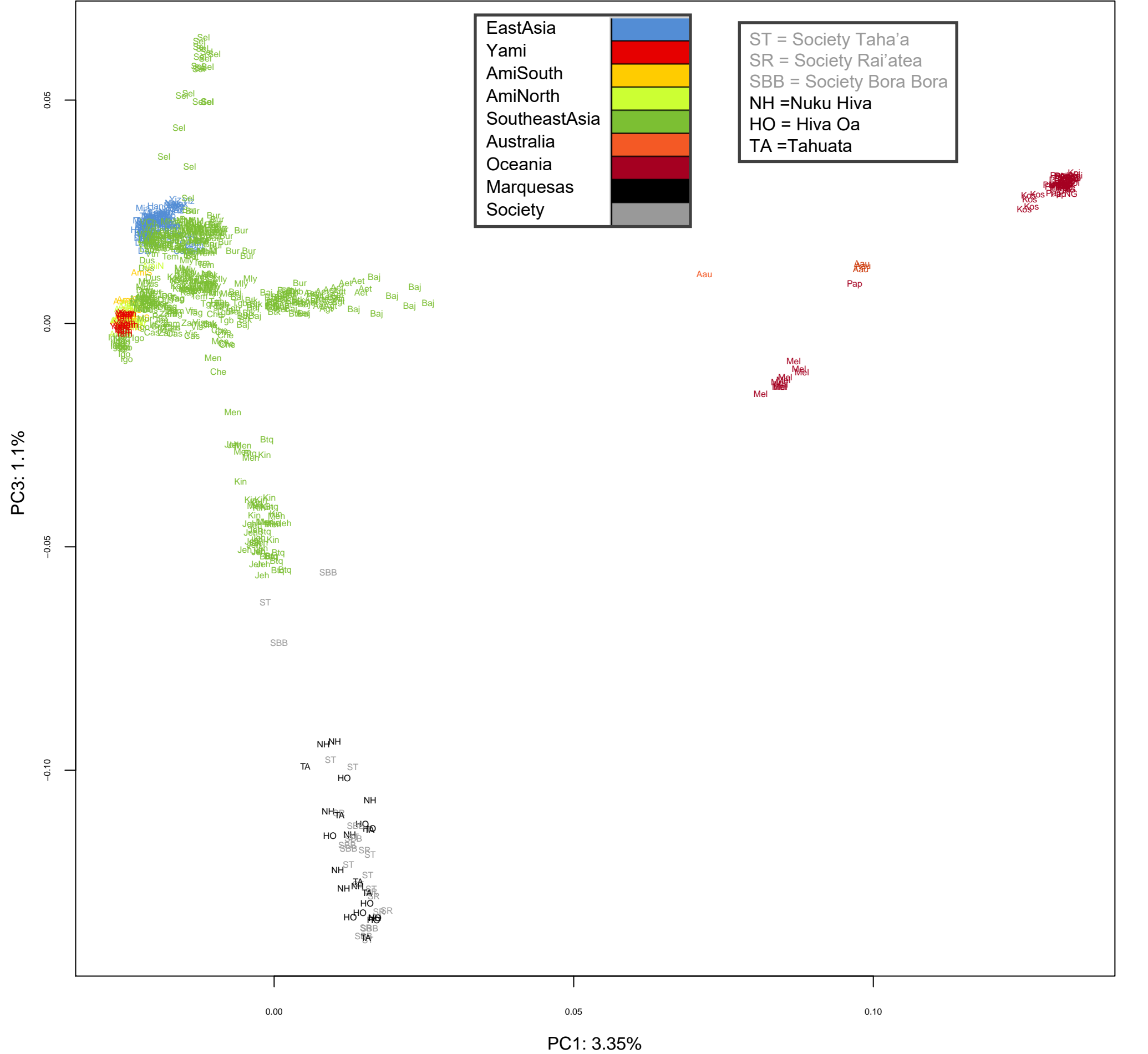

Supplement: Supplementary file 4 — Supplementary Figure 3. [file 41598_2022_8910_MOESM4_ESM.pdf]

Supplementary Figure 4. PC1/PC3 plot of East Polynesian populations.

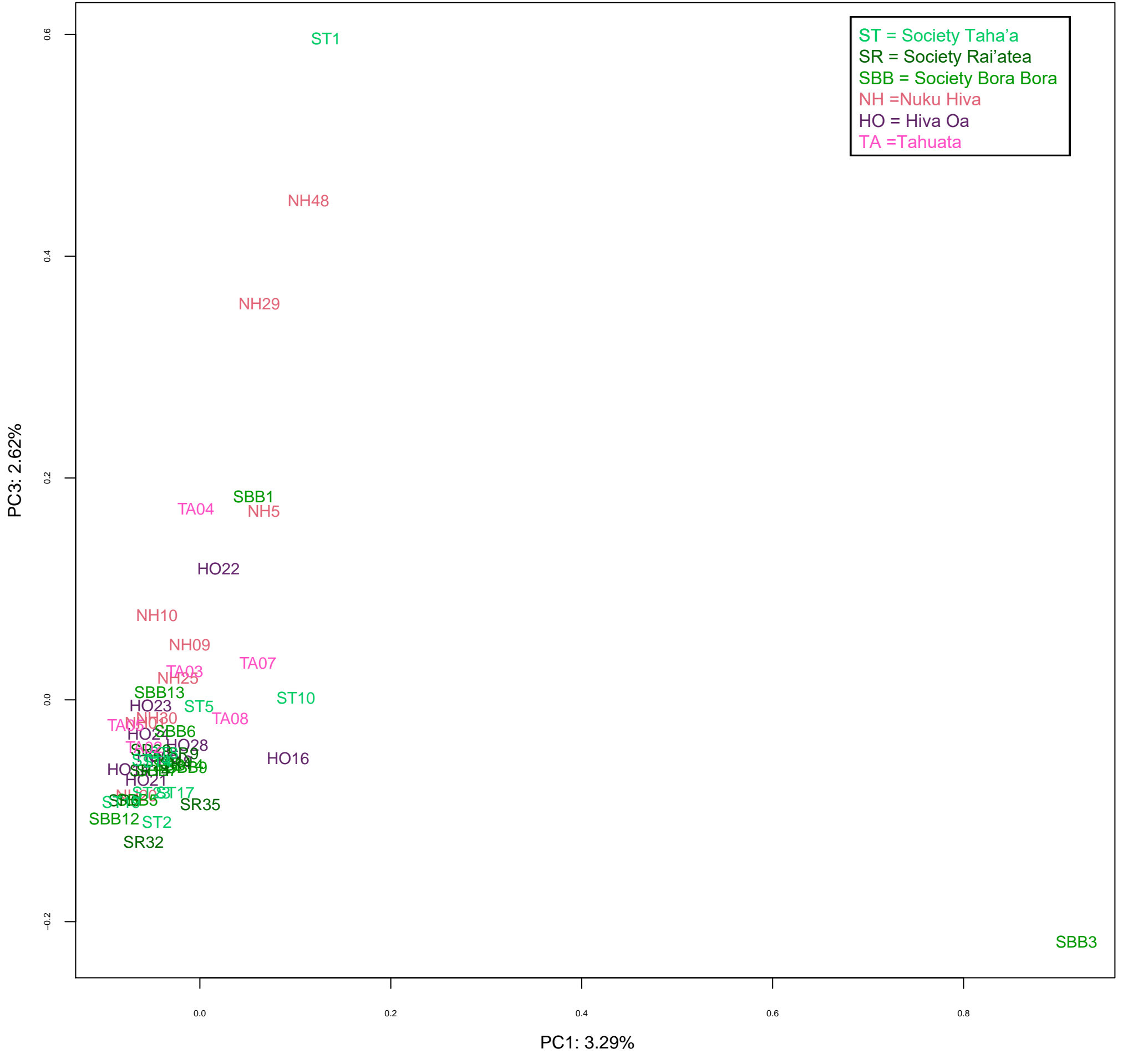

Supplement: Supplementary file 5 — Supplementary Figure 4. [file 41598_2022_8910_MOESM5_ESM.pdf]

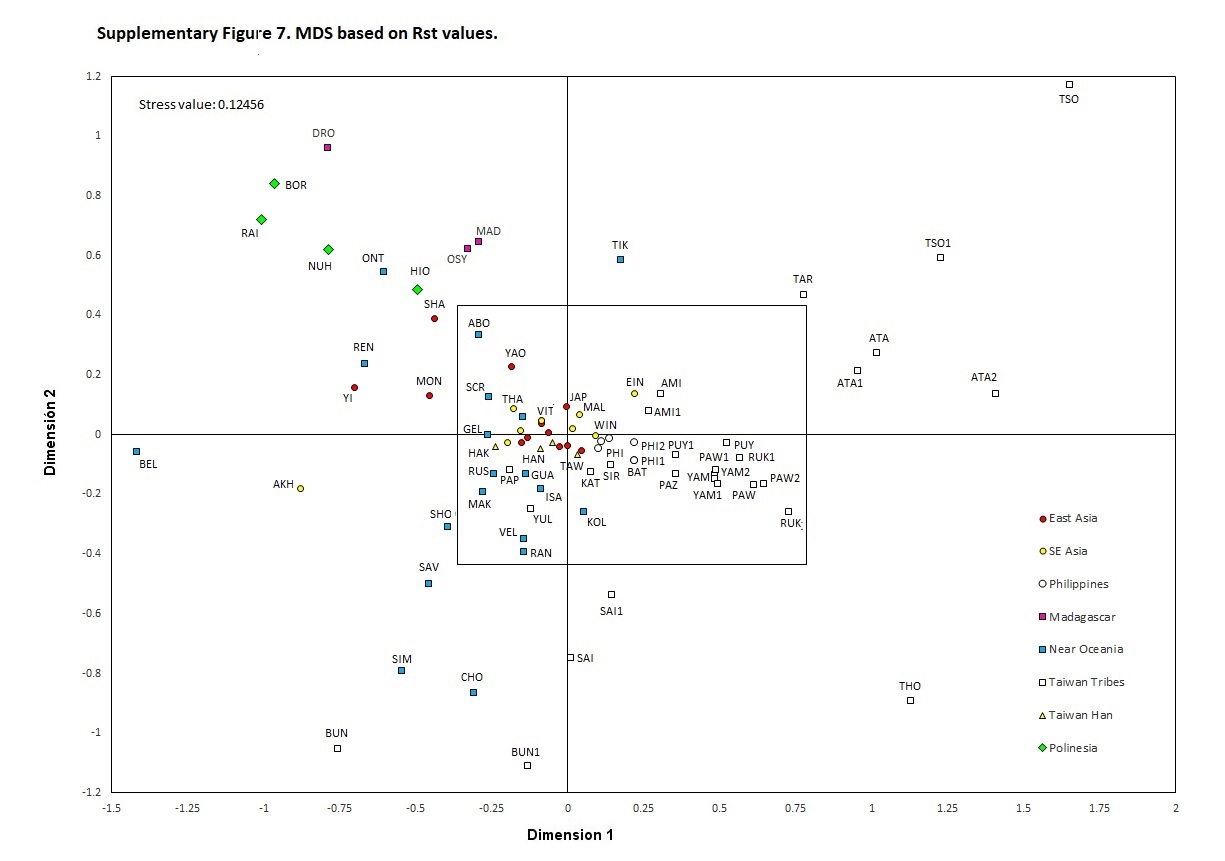

Supplement: Supplementary file 8 — Supplementary Figure 7. [file 41598_2022_8910_MOESM8_ESM.jpg]

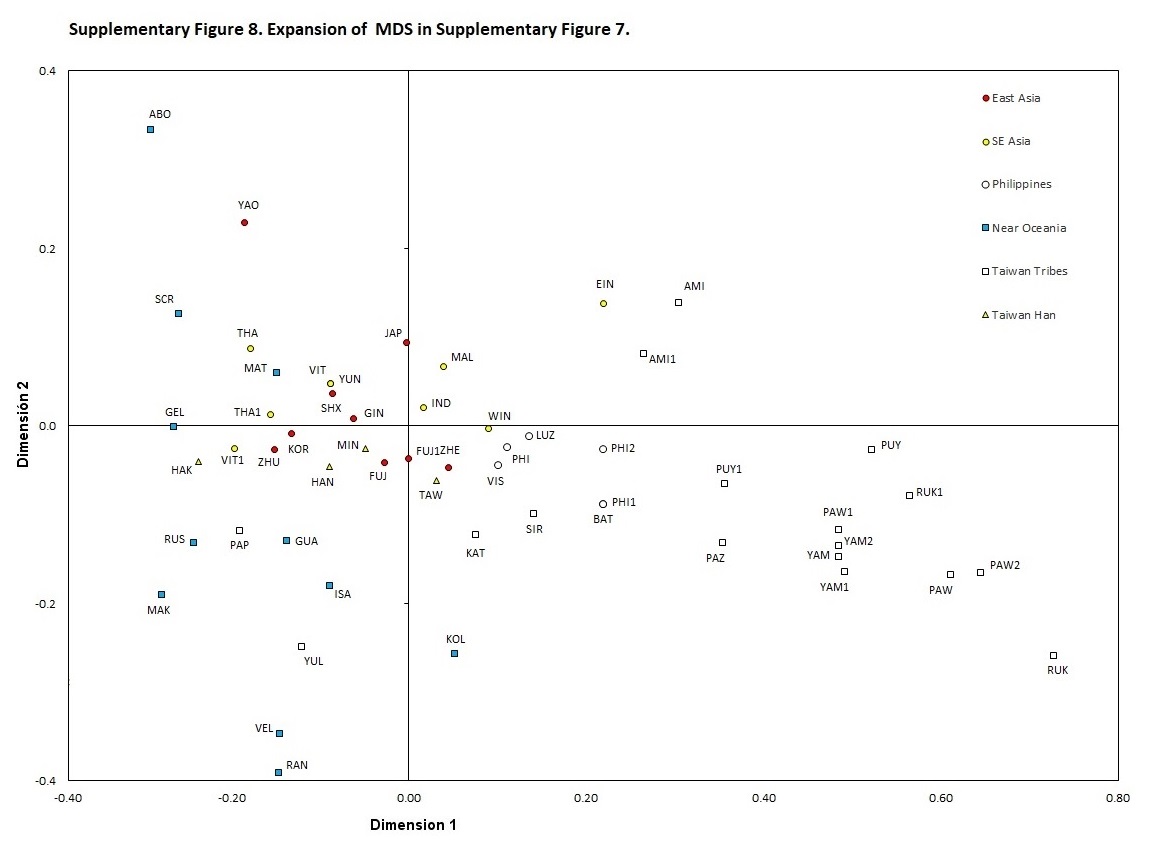

Supplement: Supplementary file 9 — Supplementary Figure 8. [file 41598_2022_8910_MOESM9_ESM.jpg]

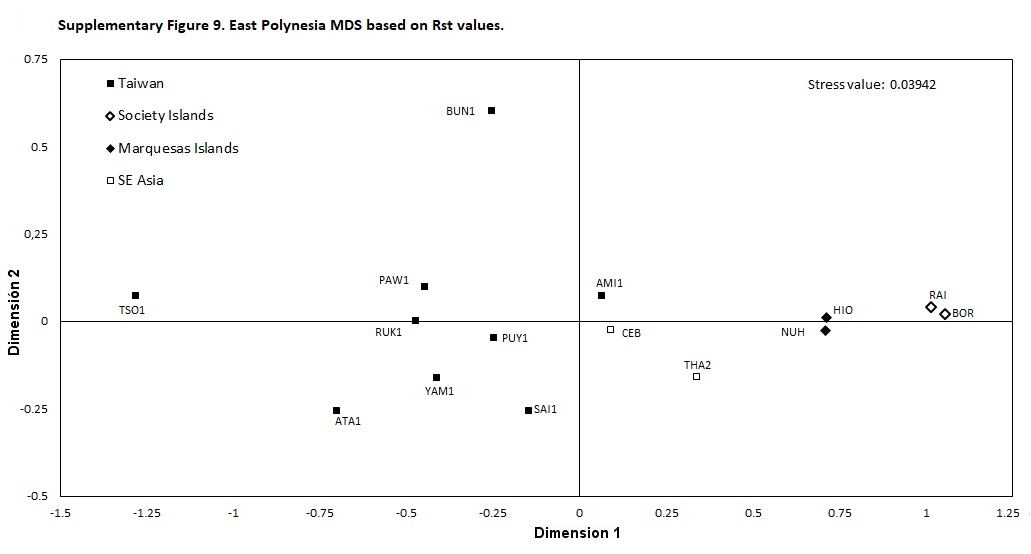

Supplement: Supplementary file 10 — Supplementary Figure 9. [file 41598_2022_8910_MOESM10_ESM.jpg]
