# Supplementary material for: Genetic characterization of populations in the Marquesas Archipelago in the context of the Austronesian expansion
Source: Sci Rep. 2022 Mar 29;12:5312. doi: 10.1038/s41598-022-08910-w (PMC8964752; doi:10.1038/s41598-022-08910-w)
Supplement: Supplementary file 27 — Supplementary Table 17. [file 41598_2022_8910_MOESM27_ESM.docx]

Accession nº Sample ID Haplogroup

MT135508 HO5 B4a1a1c

MT135509 HO22 B4a1a1m1

MT135510 HO23 B4a1a1

MT135511 NH20 B4a1a1m1

MT135512 NH25 B4a1a1a

MT135513 NH29 B4a1a1+16126

MT135514 NH35 B4a1a1c

MT135515 TA2 B4a1a1c

>MT135508.1 Homo sapiens isolate HO5 mitochondrion, complete genome

GATCACAGGTCTATCACCCTATTAACCACTCACGGGAGCTCTCCATGCATTTGGTATTTTCGTCTGGGGG

GTGTGCACGCGATAGCATTGCGAGACGCTGGAGCCGGAGCACCCTATGTCGCAGTATCTGTCTTTGATTC

CTGCCCCATCCTATTATTTATCGCACCTACGTTCAATATTACAGGCGAACATACTTACTAAAGTGTGTTA

ATTAATTAATGCTTGTAGGACATAATAATAACAATTGAATGTCTGCACAGCCGCTTTCCACACAGACATC

ATAACAAAAAATTTCCACCAAACCCCCCCCCCTCCCCCCGCTTCTGGCCACAGCACTTAAACACATCTCT

GCCAAACCCCAAAAACAAAGAACCCTAACACCAGCCTAACCAGATTTCAAATTTTATCTTTTGGCGGTAT

GCACTTTTAACAGTCACCCCCCAACTAACACATTATTTTCCCCTCCCACTCCCATACTACTAATCTCATC

AATACAACCCCCGCCCATCCTACCCAACACACACACCGCTGCTAACCCCATACCCCGAACCAACCAAACC

CCAAAGACACCCCCCACAGTTTATGTAGCTTACCTCCTCAAAGCAATACACTGAAAATGTTTAGACGGGC

TCACATCACCCCATAAACAAATAGGTTTGGTCCTAGCCTTTCTATTAGCTCTTAGTAAGATTACACATGC

AAGCATCCCCGTTCCAGTGAGTTCACCCTCTAAATCACCACGATCAAAAGGGACAAGCATCAAGCACGCA

GCAATGCAGCTCAAAACGCTTAGCCTAGCCACACCCCCACGGGAAACAGCAGTGATTAACCTTTAGCAAT

AAACGAAAGTTTAACTAAGCTATACTAACCCCAGGGTTGGTCAATTTCGTGCCAGCCACCGCGGTCACAC

GATTAACCCAAGTCAATAGAAGCCGGCGTAAAGAGTGTTTTAGATCACCCCCTCCCCAATAAAGCTAAAA

CTCACCTGAGTTGTAAAAAACTCCAGTTGACACAAAATAGACTACGAAAGTGGCTTTAACATATCTGAAC

ACACAATAGCTAAGACCCAAACTGGGATTAGATACCCCACTATGCTTAGCCCTAAACCTCAACAGTTAAA

TCAACAAAACTGCTCGCCAGAACACTACGAGCCACAGCTTAAAACTCAAAGGACCTGGCGGTGCTTTATA

TCCCTCTAGAGGAGCCTGTTCTGTAATCGATAAACCCCGATCAACCTCACCACCTCTTGCTCAGCCTATA

TACCGCCATCTTCAGCAAACCCTGATGAAGGCTACAAAGTAAGCGCAAGTACCCACGTAAAGACGTTAGG

TCAAGGTGTAGCCCATGAGGTGGCAAGAAATGGGCTACATTTTCTACCCCAGAAAACTACGATAGCCCTT

ATGAAACTTAAGGGTCGAAGGTGGATTTAGCAGTAAACTGAGAGTAGAGTGCTTAGTTGAACAGGGCCCT

GAAGCGCGTACACACCGCCCGTCACCCTCCTCAAGTATACTTCAAAGGACATTTAACTAAAACCCCTACG

CATTTATATAGAGGAGACAAGTCGTAACATGGTAAGTGTACTGGAAAGTGCACTTGGACGAACCAGAGTG

TAGCTTAACACAAAGCACCCAACTTACACTTAGGAGATTTCAACTTAACTTGACCGCTCTGAGCTAAACC

TAGCCCCAAACCCACTCCACCTTACTACCAGACAACCTTAGCCAAACCATTTACCCAAATAAAGTATAGG

CGATAGAAATTGAAACCTGGCGCAATAGATATAGTACCGCAAGGGAAAGATGAAAAATTATAACCAAGCA

TAATATAGCAAGGACTAACCCCTATACCTTCTGCATAATGAATTAACTAGAAATAACTTTGCAAGGAGAG

CCAAAGCTAAGACCCCCGAAACCAGACGAGCTACCTAAGAACAGCTAAAAGAGCACACCCGTCTATGTAG

CAAAATAGTGGGAAGATTTATAGGTAGAGGCGACAAACCTACCGAGCCTGGTGATAGCTGGTTGTCCAAG

ATAGAATCTTAGTTCAACTTTAAATTTGCCCACAGAACCCTCTAAATCCCCTTGTAAATTTAACTGTTAG

TCCAAAGAGGAACAGCTCTTTGGACACTAGGAAAAAACCTTGTAGAGAGAGTAAAAAATTTAACACCCAT

AGTAGGCCTAAAAGCAGCCACCAATTAAGAAAGCGTTCAAGCTCAACACCCACTACCTAAAAAATCCCAA

ACATATAACTGAACTCCTCACACCCAATTGGACCAATCTATCACCCTATAGAAGAACTAATGTTAGTATA

AGTAACATGAAAACATTCTCCTCCGCATAAGCCTGCGTCAGATTAAAACACTGAACTGACAATTAACAGC

CCAATATCTACAATCAACCAACAAGTCATTATTACCCTCACTGTCAACCCAACACAGGCATGCTCATAAG

GAAAGGTTAAAAAAAGTAAAAGGAACTCGGCAAATCTTACCCCGCCTGTTTACCAAAAACATCACCTCTA

GCATCACCAGTATTAGAGGCACCGCCTGCCCAGTGACACATGTTTAACGGCCGCGGTACCCTAACCGTGC

AAAGGTAGCATAATCACTTGTTCCTTAAATAGGGACCTGTATGAATGGCTCCACGAGGGTTCAGCTGTCT

CTTACTTTTAACCAGTGAAATTGACCTGCCCGTGAAGAGGCGGGCATGACACAGCAAGACGAGAAGACCC

TATGGAGCTTTAATTTATTAATGCAAACAGTACCTAACAAACCCACAGGTCCTAAACTACCAAACCTGCA

TTAAAAATTTCGGTTGGGGCGACCTCGGAGCAGAACCCAACCTCCGAGCAGTACATGCTAAGACTTCACC

AGTCAAAGCGAACTACTATACTCAATTGATCCAATAACTTGACCAACGGAACAAGTTACCCTAGGGATAA

CAGCGCAATCCTATTCTAGAGTCCATATCAACAATAGGGTTTACGACCTCGATGTTGGATCAGGACATCC

CGATGGTGCAGCCGCTATTAAAGGTTCGTTTGTTCAACGATTAAAGTCCTACGTGATCTGAGTTCAGACC

GGAGTAATCCAGGTCGGTTTCTATCTACNTTCAAATTCCTCCCTGTACGAAAGGACAAGAGAAATAAGGC

CTACTTCACAAAGCGCCTTCCCCCGTAAATGATATCATCTCAACTTAGTATTATACCCACACCCACCCAA

GAACAGGGTTTGTTAAGATGGCAGAGCCCGGTAATCGCATAAAACTTAAAACTTTACAGTCAGAGGTTCA

ATTCCTCTTCTTAACAACATACCCATGGCCAACCTCCTACTCCTCATTGTACCCATTCTAATCGCAATGG

CATTCCTAATGCTTACCGAACGAAAAATTCTAGGCTATATACAACTACGCAAAGGCCCCAACGTTGTAGG

CCCCTACGGGCTACTACAACCCTTCGCTGACGCCATAAAACTCTTCACCAAAGAGCCCCTAAAACCCGCC

ACATCTACCATCACCCTCTACATCACCGCCCCGACCTTAGCTCTCACCATCGCTCTTCTACTATGAACCC

CCCTCCCCATACCCAACCCCCTGGTCAACCTCAACCTAGGCCTCCTATTTATTCTAGCCACCTCTAGCCT

AGCCGTTTACTCAATCCTCTGATCAGGGTGAGCATCAAACTCAAACTACGCCCTGATCGGCGCACTGCGA

GCAGTAGCCCAAACAATCTCATATGAAGTCACCCTAGCCATCATTCTACTATCAACATTACTAATAAGTG

GCTCCTTTAACCTCTCCACCCTTATCACAACACAAGAACACCTCTGATTACTCCTGCCATCATGACCCTT

GGCCATAATATGATTTATCTCCACACTAGCAGAGACCAACCGAACCCCCTTCGACCTTGCCGAAGGGGAG

TCCGAACTAGTCTCAGGCTTCAACATCGAATACGCCGCAGGCCCCTTCGCCCTATTCTTCATAGCCGAAT

ACACAAACATTATTATAATAAACACCCTCACCACTACAATCTTCCTAGGAACAACATATGACGCACTCTC

CCCTGAACTCTACACAACATATTTTGTCACCAAGACCCTACTTCTAACCTCCCTGTTCTTATGAATTCGA

ACAGCATACCCCCGATTCCGCTACGACCAACTCATACACCTCCTATGAAAAAACTTCCTACCACTCACCC

TAGCATTACTTATATGATATGTCTCCATACCCATTACAATCTCCAGCATTCCCCCTCAAACCTAAGAAAT

ATGTCTGATAAAAGAGTTACTTTGATAGAGTAAATAATAGGAGCTTAAACCCCCTTATTTCTAGGACTAT

GAGAATCGAACCCATCCCTGAGAATCCAAAATTCTCCGTGCCACCTATCACACCCCATCCTAAAGTAAGG

TCAGCTAAATAAGCTATCGGGCCCATACCCCGAAAATGTTGGTTATACCCTTCCCGTACTAATTAATCCC

CTGGCCCAACCCGTCATCTACTCTACCATCTTTGCAGGCACACTCATCACAGCGCTAAGCTCGCACTGAT

TTTTTACCTGAGTAGGCCTAGAAATAAACATGCTAGCTTTTATTCCAGTTCTAACCAAAAAAATAAACCC

TCGTTCCACAGAAGCTGCCATCAAGTATTTCCTCACGCAAGCAACCGCATCCATAATCCTTCTAATAGCT

ATCCTCTTCAACAATATACTCTCCGGACAATGAACCATAACCAATACTACCAATCAATACTCATCATTAA

TAATCATAATAGCTATAGCAATAAAACTAGGAATAGCCCCCTTTCACTTCTGAGTCCCAGAGGTTACCCA

AGGCACCCCTCTGACATCCGGCCTGCTTCTTCTCACATGACAAAAACTAGCCCCCATCTCAATCATATAC

CAAATCTCTCCCTCACTAAACGTAAGCCTTCTCCTCACTCTCTCAATCTTATCCATCATAGCAGGCAGTT

GAGGTGGATTAAACCAAACCCAGCTACGCAAAATCTTAGCATACTCCTCAATTACCCACATAGGATGAAT

AATAGCAGTTCTACCGTACAACCCTAACATAACCATTCTTAATTTAACTATTTATATTATCCTAACTACT

ACCGCATTCCTACTACTCAACTTAAACTCCAGCACCACGACCCTACTACTATCTCGCACCTGAAACAAGC

TAACATGACTAACACCCTTAATTCCATCCACCCTCCTCTCCCTAGGAGGCCTGCCCCCGCTAACCGGCTT

TTTGCCCAAATGGGCCATTATCGAAGAATTCACAAAAAACAATAGCCTCATCATCCCCACCATCATAGCC

ACCATCACCCTCCTTAACCTCTACTTCTACCTACGCCTAATCTACTCCACCTCAATCACACTACTCCCCA

TATCTAACAACGTAAAAATAAAATGACAGTTTGAACATACAAAACCCACCCCATTCCTCCCCACACTCAT

CGCCCTCACCACGCTACTCCTACCTATCTCCCCTTTTATACTAATAATCTTATAGAAATTTAGGTTAAAT

ACAGACCAAGAGCCTTCAAAGCCCTCAGTAAGTTGCAATACTTAATTTCTGTAACAGCTAAGGACTGCAA

AACCCCACTCTGCATCAACTGAACGCAAATCAGCCACTTTAATTAAGCTAAGCCCTTACTAGACCAATGG

GACTTAAACCCACAAACACTTAGTTAACAGCTAAGCACCCTAATCAACTGGCTTCAATCTACTTCTCCCG

CCGCCGGGAAAAAAGGCGGGAGAAGCCCCGGCAGGTTTGAAGCTGCTTCTTCGAATTTGCAATTCAATAT

GAAAATCACCTCGGAGCTGGTAAAAAGAGGCCTAACCCCTGTCTTTAGATTTACAGTCCAATGCTTCACT

CAGCCATTTTACCTCACCCCCACTGATGTTCGCCGACCGTTGACTATTCTCTACAAACCACAAAGACATT

GGAACACTATACCTATTATTCGGCGCATGAGCTGGAGTCCTAGGCACAGCTCTAAGCCTCCTTATTCGAG

CCGAGCTGGGCCAGCCAGGCAACCTTCTAGGTAACGACCACATCTACAACGTTATCGTCACAGCCCATGC

ATTTGTAATAATCTTCTTCATAGTAATACCCATCATAATCGGAGGCTTTGGCAACTGACTAGTTCCCCTA

ATAATCGGTGCCCCCGATATGGCGTTTCCCCGCATAAACAACATAAGCTTCTGACTCTTACCTCCCTCTC

TCCTACTCCTGCTCGCATCTGCTATAGTGGAGGCCGGAGCAGGAACAGGTTGAACAGTCTACCCTCCCTT

AGCAGGGAACTACTCCCACCCTGGAGCCTCCGTAGACCTAACCATCTTCTCCTTACACCTAGCAGGTGTC

TCCTCTATCTTAGGGGCCATCAATTTCATCACAACAATTATCAATATAAAACCCCCTGCCATAACCCAAT

ACCAAACGCCCCTCTTCGTCTGATCCGTCCTAATCACAGCAGTCCTACTTCTCCTATCTCTCCCAGTCCT

AGCTGCTGGCATCACTATACTACTAACAGACCGCAACCTCAACACCACCTTCTTCGACCCCGCCGGAGGA

GGAGACCCCATTCTATACCAACACCTATTCTGATTTTTCGGTCACCCTGAAGTTTATATTCTTATCCTAC

CAGGCTTCGGAATAATCTCCCATATTGTAACTTACTACTCCGGAAAAAAAGAACCATTTGGATACATAGG

CATGGTCTGAGCTATGATATCAATTGGCTTCCTAGGGTTTATCGTGTGAGCACACCATATATTTACAGTA

GGAATAGACGTAGACACACGAGCATATTTCACCTCCGCTACCATAATCATCGCTATCCCCACCGGCGTCA

AAGTATTTAGCTGACTCGCCACACTCCACGGAAGCAATATGAAATGATCTGCTGCAGTGCTCTGAGCCCT

AGGATTCATCTTTCTTTTCACCGTAGGTGGCCTGACTGGCATTGTATTAGCAAACTCATCACTAGACATC

GTACTACACGACACGTACTACGTTGTAGCTCACTTCCACTATGTCCTATCAATAGGAGCTGTATTTGCCA

TCATAGGAGGCTTCATTCACTGATTTCCCCTATTCTCAGGCTACACCCTAGACCAAACCTACGCCAAAAT

CCATTTCACTATCATATTCATCGGCGTAAATCTAACTTTCTTCCCACAACACTTTCTCGGCCTATCCGGA

ATGCCCCGACGTTACTCGGACTACCCCGATGCATACACCACATGAAACATCCTATCATCTGTAGGCTCAT

TCATTTCTCTAACAGCAGTAATATTAATAATTTTCATGATTTGAGAAGCCTTCGCTTCGAAGCGAAAAGT

CCTAATAGTAGAAGAACCCTCCATAAACCTGGAGTGACTATATGGATGCCCCCCACCCTACCACACATTC

GAAGAACCCGTATACATAAAATCTAGACAAAAAAGGAAGGAATCGAACCCCCCAAAGCTGGTTTCAAGCC

AACCCCATGGCCTCCATGACTTTTTCAAAAAGGTATTAGAAAAACCATTTCATAACTTTGTCAAAGTTAA

ATTATAGGCTAAATCCTATATATCTTAATGGCACATGCAGCGCAAGTAGGTCTACAAGACGCTACTTCCC

CTATCATAGAAGAGCTTATCACCTTTCATGATCACGCCCTCATAATCATTTTCCTTATCTGCTTCCTAGT

CCTGTATGCCCTTTTCCTAACACTCACAACAAAACTAACTAATACTAACATCTCAGACGCTCAGGAAATA

GAAACCGTCTGAACTATCCTGCCCGCCATCATCCTAGTCCTCATCGCCCTCCCATCCCTACGCATCCTTT

ACATAACAGACGAGGTCAACGATCCCTCCCTTACCATCAAATCAATTGGCCACCAATGGTACTGAACCTA

CGAGTACACCGACTACGGCGGACTAATCTTCAACTCCTACATACTTCCCCCATTATTCCTAGAACCAGGC

GACCTGCGACTCCTTGACGTTGACAATCGAGTAGTACTCCCGATTGAAGCCCCCATTCGTATAATAATTA

CATCACAAGACGTCTTGCACTCATGAGCTGTCCCCACATTAGGCTTAAAAACAGATGCAATTCCCGGACG

TCTAAACCAAACCACTTTCACCGCTACACGACCGGGGGTATACTACGGTCAATGCTCTGAAATCTGTGGA

GCAAACCACAGTTTCATGCCCATCGTCCTAGAATTAATTCCCCTAAAAATCTTTGAAATAGGGCCCGTAT

TTACCCTATAGCACCCCCTCTAGAGCCCACTGTAAAGCTAACTTAGCATTAACCTTTTAAGTTAAAGATT

AAGAGAACCAACACCTCTTTACAGTGAAATGCCCCAACTAAATACTACCGTATGGCCCACCATAATTACC

CCCATACTCCTTACACTATTCCTCATCACCCAACTAAAAATATTAAACACAAACTACCACCTACCTCCCT

CACCAAAGCCCATAAAAATAAAAAATTATAACAAACCCTGAGAACCAAAATGAACGAAAATCTGTTCGCT

TCATTCATTGCCCCCACAATCCTAGGCCTACCCGCCGCAGTACTGATCATTCTATTTCCCCCTCTATTGA

TCCCCACCTCCAAATATCTCATCAACAACCGACTAATCACCACCCAACAATGACTAATCAAACTAACCTC

AAAACAAATGATAACCATACACAACACTAAAGGACGAACCTGATCTCTTATACTAGTATCCTTAATCATT

TTTATTGCCACAACTAACCTCCTCGGACTCCTGCCTCACTCATTTACACCAACCACCCAACTATCTATAA

ACCTAGCCATGGCCATCCCCTTATGAGCGGGCGCAGTGATTATAGGCTTTCGCTCTAAGATTAAAAATGC

CCTAGCCCACTTCTTACCACAAGGCACACCTACACCCCTTATCCCCATACTAGTTATTATCGAAACCATC

AGCCTACTCATTCAACCAATAGCCCTGGCCGTACGCCTAACCGCTAACATTACTGCAGGCCACCTACTCA

TGCACCTAATTGGAAGCGCCACCCTAGCAATATCAACCATTAACCTTCCCTCTACACTTATCATCTTCAC

AATTCTAATTCTACTAACTATCCTAGAAATCGCTGTCGCCTTAATCCAAGCCTACGTTTTCACACTTCTA

GTAAGCCTCTACCTGCACGACAACACATAATGACCCACCAATCACATGCCTATCATATAGTAAAACCCAG

CCCATGACCCCTAACAGGGGCCCTCTCAGCCCTCCTAATGACCTCCGGCCTAGCCATGTGATTTCACTTC

CACTCCATAACGCTCCTCATACTAGGCCTACTAACCAACACACTAACCATATACCAATGATGGCGCGATG

TAACACGAGAAAGCACATACCAAGGCCACCACACACCACCTGTCCAAAAAGGCCTTCGATACGGGATAAT

CCTATTTATTACCTCAGAAGTTTTTTTCTTCGCAGGATTTTTCTGAGCCTTTTACCACTCCAGCCTAGCC

CCTACCCCCCAATTAGGAGGGCACTGGCCCCCAACAGGCATCACCCCGCTAAATCCCCTAGAAGTCCCAC

TCCTAAACACATCCGTATTACTCGCATCAGGAGTATCAATCACCTGAGCTCACCATAGTCTAATAGAAAA

CAACCGAAACCAAATAATTCAAGCACTGCTTATTACAATTTTACTGGGTCTCTATTTTACCCTCCTACAA

GCCTCAGAGTACTTCGAGTCTCCCTTCACCATTTCCGACGGCATCTACGGCTCAACATTTTTTGTAGCCA

CAGGCTTCCACGGACTTCACGTCATTATTGGCTCAACTTTCCTCACTATCTGCTTCATCCGCCAACTAAT

ATTTCACTTTACATCCAAACATCACTTTGGCTTCGAAGCCGCCGCCTGATACTGGCATTTTGTAGATGTG

GTTTGACTATTTCTGTATGTCTCCATCTATTGATGAGGGTCTTACTCTTTTAGTATAAATAGTACCGTTA

ACTTCCAATTAACTAGTTTTGACAACATTCAAAAAAGAGTAATAAACTTCGCCTTAATTTTAATAATCAA

CACCCTCCTAGCCTTACTACTAATAATTATTACATTTTGACTACCACAACTCAACGGCTACATAGAAAAA

TCCACCCCTTACGAGTGCGGCTTCGACCCTATATCCCCCGCCCGCGTCCCTTTCTCCATAAAATTCTTCT

TAGTAGCTATCACCTTCTTATTATTTGATCTAGAAATTGCCCTCCTTTTACCCCTACCATGAGCCCTACA

AACAACTAACCTGCCACTAATAGTTATGTCATCCCTCTTATTAATCATCATCCTAGCCCTAAGTCTGGCC

TATGAGTGACTACAAAAAGGATTAGACTGAACCGAATTGGTATATAGTTTAAACAAAACGAATGATTTCG

ACTCATTAAATTATGATAATCATATTTACCAAATGCCCCTCATTTACATAAATATTATACTAGCATTTAC

CATCTCACTTCTAGGAATACTAGTATATCGCTCACACCTCATATCCTCCCTACTATGCCTAGAAGGAATA

ATACTATCGCTGTTCATTATAGCTACTCTCATAACCCTCAACACCCACTCCCTCTTAGCCAATATTGTGC

CTATTGCCATACTAGTCTTTGCCGCCTGCGAAGCAGCGGTGGGCCTAGCCCTACTAGTCTCAATCTCCAA

CACATATGGCCTAGACTACGTACATAACCTAAACCTACTCCAATGCTAAAACTAATCGTCCCAACAATTA

TATTACTACCACTGACATGACTTTCCAAAAAACACATAATTTGAATCAACACAACCACCCACAGCCTAAT

TATTAGCATCATCCCTCTACTATTTTTTAACCAAATCAACAACAACCTATTTAGCTGTTCCCCAACCTTT

TCCTCCGACCCCCTAACAACCCCCCTCCTAATACTAACTACCTGACTCCTACCCCTCACAATCATGGCAA

GCCAACGCCACTTATCCAGTGAACCACTATCACGAAAAAAACTCTACCTCTCTATACTAATCTCCCTACA

AATCTCCTTAATTATAACATTCACAGCCACAGAACTAATCATATTTTATATCTTCTTCGAAACCACACTT

ATCCCCACCTTGGCTATCATCACCCGATGAGGCAACCAGCCAGAACGCCTGAACGCAGGCACATACTTCC

TATTCTACACCCTAGTAGGCTCCCTTCCCCTACTCATCGCACTAATTTACACTCACAACACCCTAGGCTC

ACTAAACATTCTACTACTCACTCTCACTGCCCAAGAACTATCAAACTCCTGAGCCAACAACTTAATATGA

CTAGCTTACACAATAGCTTTTATAGTAAAGATACCTCTTTACGGACTCCACTTATGACTCCCTAAAGCCC

ATGTCGAAGCCCCCATCGCTGGGTCAATAGTACTTGCCGCAGTACTCTTAAAACTAGGCGGCTATGGTAT

AATACGCCTCACACTCATTCTCAACCCCCTGACAAAACACATAGCCTACCCCTTCCTTGTACTATCCCTA

TGAGGCATAATTATAACAAGCTCCATCTGCCTACGACAAACAGACCTAAAATCGCTCATTGCATACTCTT

CAATCAGCCACATAGCCCTCGTAGTAACAGCCATTCTCATCCAAACCCCCTGAAGCTTCACCGGCGCAGT

CATTCTCATAATCGCCCACGGACTTACATCCTCATTACTATTCTGCCTAGCAAACTCAAACTACGAACGC

ACTCACAGTCGCATCATAATCCTCTCTCAAGGACTTCAAACTCTACTCCCACTAATAGCTTTTTGATGAC

TTCTAGCAAGCCTCGCTAACCTCGCCTTACCCCCCACTATTAACCTACTGGGAGAACTCTCTGTGCTAGT

AACCACGTTCTCCTGATCAAATATCACTCTCCTACTTACAGGACTCAACATACTAGTCACAGCCCTATAC

TCCCTCTACATATTTACCACAACACAATGGGGCTCACTCACCCACCACATTAACAACATAAAACCCTCAT

TCACACGAGAAAACACCCTCATGTTCATACACCTATCCCCCATTCTCCTCCTATCCCTCAACCCCGACAT

CATTACCGGGTTTTCCTCTTGTAAATATAGTTTAACCAAAACATCAGATTGTGAATCTGACAACAGAGGC

TTACGACCCCTTATTTACCGAGAAAGCTCACAAGAACTGCTAACTCATGCCTCCATGTCTAACAACATGG

CTTTCTCAACTTTTAAAGGATAACAGCTATCCATTGGTCTTAGGCCCCAAAAATTTTGGTGCAACTCCAA

ATAAAAGTAATAACCATGCACACTACTATAACCACCCTAACCCTGACTTCCCTAATTCCCCCCATCCTTA

CCACCCTCGTTAACCCTAACAAAAAAAACTCATACCCCCATTATGTAAAATCCATTGTCGCATCCACCTT

TATTATCAGTCTCTTCCCCACAACAATATTCATGTGCCTAGACCAAGAAGTTATTATCTCGAACTGACAC

TGAGCCACAACCCAAACAACCCAGCTCTCCCTAAGCTTCAAACTAGACTACTTCTCCATAATATTCATCC

CTGTAGCATTGTTCGTTACATGGTCCATCATAGAATTCTCACTGTGATATATAAACTCAGACCCAAACAT

TAATCAGTTCTTCAAATATCTACTCATCTTCCTAATTACCATACTAATCTTAGTTACCGCTAACAACCTA

TTCCAACTGTTCATCGGCTGAGAGGGCGTAGGAATTATATCCTTCTTGCTCATCAGTTGATGATACGCCC

GAGCAGATGCCAACACAGCAGCCATTCAAGCAATCCTATACAACCGTATCGGCGATATCGGTTTCATCCT

CGCCTTAGCATGATTTATCCTACACTCCAACTCATGAGACCCACAACAAATAGCCCTTCTAAACGCTAAT

CCAAGCCTCACCCCACTACTAGGCCTCCTCCTAGCAGCAGCAGGCAAATCAGCCCAATTAGGTCTCCACC

CCTGACTCCCCTCAGCCATAGAAGGCCCCACCCCAGTCTCAGCCCTACTCCACTCAAGCACTATAGTTGT

AGCAGGAATCTTCTTACTCATCCGCTTCCACCCCCTAGCAGAAAATAGCCCACTAATCCAAACTCTAACA

CTATGCTTAGGCGCTATCACCACTCTGTTCGCAGCAGTCTGCGCCCTTACACAAAATGACATCAAAAAAA

TCGTAGCCTTCTCCACTTCAAGTCAACTAGGACTCATAATAGTTACAATCGGCATCAACCAACCACACCT

AGCATTCCTGCACATCTGTACCCACGCCTTCTTCAAAGCCATACTATTTATGTGCTCCGGGTCCATCATC

CACAACCTTAACAATGAACAAGATATTCGAAAAATAGGAGGACTACTCAAAACCATACCTCTCACTTCAA

CCTCCCTCACCATTGGCAGCCTAGCATTAGCAGGAATACCTTTCCTCACAGGTTTCTACTCCAAAGACCA

CATCATCGAAACCGCAAACATATCATACACAAACGCCTGAGCCCTATCTATTACTCTCATCGCTACCTCC

CTGACAAGCGCCTATAGCACTCGAATAATTCTTCTCACCCTAACAGGTCAACCTCGCTTCCCCACCCTTA

CTAACATTAACGAAAATAACCCCACCCTACTAAACCCCATTAAACGCCTGGCAGCCGGAAGCCTATTCGC

AGGATTTCTCATTACTAACAACATTTCCCCCGCATCCCCCTTCCAAACAACAATCCCCCTCTACCTAAAA

CTCACAGCCCTCGCTGTCACTTTCCTAGGACTTCTAACAGCCCTAGACCTCAACTACCTAACCAACAAAC

TTAAAATAAAATCCCCACTATGCACATTTTATTTCTCCAACATACTCGGATTCTACCCTAGCATCACACA

CCGCACAATCCCCTATCTAGGCCTTCTTACGAGCCAAAACCTGCCCCTACTCCTCCTAGACCTAACCTGA

CTAGAAAAGCTATTGCCTAAAACAATTTCACAGCACCAAATCTCCACCTCCATCATCACCTCAACCCAAA

AAGGCATAATTAAACTTTACTTCCTCTCTTTCTTCTTCCCACTCATCCTAACCCTACTCCTAATCACATA

ACCTATTCCCCCGAGCAATCTCAATTACAATATATACACCAACAAACAATGTTCAACCAGTAACTACTAC

TAATCAACGCCCATAATCATACAAAGCCCCCGCACCAATAGGATCCTCCCGAATCAACCCTGACCCCTCT

CCTTCATAAATTATTCAGCTTCCTACACTATTAAAGTTTACCACAACCACCACCCCATCATACTCTTTCA

CCCACAGCACCAATCCTACCTCCATCGCTAACCCCACTAAAACACTCACCAAGACCTCAACCCCTGACCC

CCATGCCTCAGGATACTCCTCAATAGCCATCGCTGTAGTATATCCAAAGACAACCATCATTCCCCCTAAA

TAAATTAAAAAAACTATTAAACCCATATAACCTCCCCCAAAATTCAGAATAATAACACACCCGACCACAC

CGCTAACAATCAATACTAAACCCCCATAAATAGGAGAAGGCTTAGAAGAAAACCCCACAAACCCCATTAC

TAAACCCACACTCAACAGAAACAAAGCATACATCATTATTCTCGCACGGACTACAACCACGACCAATGAT

ATGAAAAACCATCGTTGTATTTCAACTACAAGAACACCAATGACCCCAATACGCAAAACTAACCCCCTAA

TAAAATTAATTAACCACTCATTCATCGACCTCCCCACCCCATCCAACATCTCCGCATGATGAAACTTCGG

CTCACTCCTTGGCGCCTGCCTGATCCTCCAAATCACCACAGGACTATTCCTAGCCATGCACTACTCACCA

GACGCCTCAACCGCCTTTTCATCAATCGCCCACATCACTCGAGACGTAAATTATGGCTGAATCATCCGCT

ACCTTCACGCCAATGGCGCCTCAATATTCTTTATCTGCCTCTTCCTACACATCGGGCGAGGCCTATATTA

CGGATCATTTCTCTACTCAGAAACCTGAAACATCGGCATTATCCTCCTGCTTGCAACTATAGCAACAGCC

TTCATAGGCTATGTCCTCCCGTGAGGCCAAATATCATTCTGAGGGGCCACAGTAATTACAAACTTACTAT

CCGCCATCCCATACATTGGGACAGACCTAGTTCAATGAATCTGAGGAGGCTACTCAGTAGACAGTCCCAC

CCTCACACGATTCTTTACCTTTCACTTCATCTTGCCCTTCATTATTGCAGCCCTAGCAGCACTCCACCTC

CTATTCTTGCACGAAACGGGATCAAACAACCCCCTAGGAATCACCTCCCATTCCGATAAAATCACCTTCC

ACCCTTACTACACAATCAAAGACGCCCTCGGCTTACTTCTCTTCCTTCTCTCCTTAATGACATTAACACT

ATTCTCACCAGACCTCCTAGGCGACCCAGACAATTATACCCTAGCCAACCCCTTAAACACCCCTCCCCAC

ATCAAGCCCGAATGATATTTCCTATTCGCCTACACAATTCTCCGATCCGTCCCTAACAAACTAGGAGGCG

TCCTTGCCCTATTACTATCCATCCTCATCCTAGCAATAATCCCCATCCTCCATATATCCAAACAACAAAG

CATAATATTTCGCCCACTAAGCCAATCACTTTATTGACTCCTAGCCGCAGACCTCCTCGTTCTAACCTGA

ATCGGAGGACAACCAGTAAGCTACCCTTTTACCATCATTGGACAAGTAGCATCCGTACTATACTTCACAA

CAATCCTAATCCTAATACCAACTATCTCCCTAATTGAAAACAAAATACTCAAATGGGCCTGTCCTTGTAG

TATAAACTAATACGCCAGTCTTGTAAACCGGAGATGAAAACCTTTTTCCAAGGACAAATCAGAGAAAAAG

TCTTTAACTCCACCATTAGCACCCAAAGCTAAGATTCTAATTTAAACTATTCTCTGTTCTTTCATGGGGA

AGCAGATTTGGGTACCACCCAAGTATTGACTCACCCATCAACAACCGCTATGTATTTCGTACATTACTGC

CAGCCACCATGAATATTGTACGGTACCATAAATACTTGACCACCTGTAGTACATAAAAACCCAATCCACA

TCAACCCCCCCCCCCCATGCTTACAAGCAAGTACAGCAACCAACCCTCAACTATCACACATCAACTGCAG

CTCCAAAGCCACCTCTCACCCACTAGGATACCAACAAACCTACCCACCCTTAACAGTACATAGTACATAA

AGCCATTTACCGTACATAGCACATTACAGTCAAATCCCTTCTCGTCCCCATGGATGACCCCCCTCAGATA

GGGGTCCCTTGACCACCATCCTCCGTGAAATCAATATCCCGCACAAGAGTGCTACTCTCCTCGCTCCGGG

CCCATAACACTTGGGGGTAGCTAAAGTGAACTGTATCCGACATCTGGTTCCTACTTCAGGGCCATAAAGC

CTAAATAGCCCACACGTTCCCCTTAAATAAGACATCACGATG

>MT135509.1 Homo sapiens isolate HO22 mitochondrion, complete genome

GATCACAGGTCTATCACCCTATTAACCACTCACGGGAGCTCTCCATGCATTTGGTATTTTCGTCTGGGGG

GTGTGCACGCGATAGCATTGCGAGACGCTGGAGCCGGAGCACCCTATGTCGCAGTATCTGTCTTTGATTC

CTGCCCCATCTTATTATTTATCGCACCTACGTTCAATATTACAGGCGAACATACTTACTAAAGTGTGTTA

ATTAATTAATGCTTGTAGGACATAATAATAACAATTGAATGTCTGCACAGCCGCTTTCCACACAGACATC

ATAACAAAAAATTTCCACCAAACCCCCCCCTCCCCCCGCTTCTGGCCACAGCACTTAAACACATCTCTGC

CAAACCCCAAAAACAAAGAACCCTAACACCAGCCTAACCAGATTTCAAATTTTATCTTTTGGCGGTATGC

ACTTTTAACAGTCACCCCCCAACTAACACATTATTTTCCCCTCCCACTCCCATACTACTAATCTCATCAA

TACAACCCCCGCCCATCCTACCCAGCACACACACCGCTGCTAACCCCATACCCCGAACCAACCAAACCCC

AAAGACACCCCCCACAGTTTATGTAGCTTACCTCCTCAAAGCAATACACTGAAAATGTTTAGACGGGCTC

ACATCACCCCATAAACAAATAGGTTTGGTCCTAGCCTTTCTATTAGCTCTTAGTAAGATTACACATGCAA

GCATCCCCGTTCCAGTGAGTTCACCCTCTAAATCACCACGATCAAAAGGGACAAGCATCAAGCACGCAGC

AATGCAGCTCAAAACGCTTAGCCTAGCCACACCCCCACGGGAAACAGCAGTGATTAACCTTTAGCAATAA

ACGAAAGTTTAACTAAGCTATACTAACCCCAGGGTTGGTCAATTTCGTGCCAGCCACCGCGGTCACACGA

TTAACCCAAGTCAATAGAAGCCGGCGTAAAGAGTGTTTTAGATCACCCCCTCCCCAATAAAGCTAAAACT

CACCTGAGTTGTAAAAAACTCCAGTTGACACAAAATAGACTACGAAAGTGGCTTTAACATATCTGAACAC

ACAATAGCTAAGACCCAAACTGGGATTAGATACCCCACTATGCTTAGCCCTAAACCTCAACAGTTAAATC

AACAAAACTGCTCGCCAGAACACTACGAGCCACAGCTTAAAACTCAAAGGACCTGGCGGTGCTTCATATC

CCTCTAGAGGAGCCTGTTCTGTAATCGATAAACCCCGATCAACCTCACCACCTCTTGCTCAGCCTATATA

CCGCCATCTTCAGCAAACCCTGATGAAGGCTACAAAGTAAGCGCAAGTACCCACGTAAAGACGTTAGGTC

AAGGTGTAGCCCATGAGGTGGCAAGAAATGGGCTACATTTTCTACCCCAGAAAACTACGATAGCCCTTAT

GAAACTTAAGGGTCGAAGGTGGATTTAGCAGTAAACTGAGAGTAGAGTGCTTAGTTGAACAGGGCCCTGA

AGCGCGTACACACCGCCCGTCACCCTCCTCAAGTATACTTCAAAGGACATTTAACTAAAACCCCTACGCA

TTTATATAGAGGAGACAAGTCGTAACATGGTAAGTGTACTGGAAAGTGCACTTGGACGAACCAGAGTGTA

GCTTAACACAAAGCACCCAACTTACACTTAGGAGATTTCAACTTAACTTGACCGCTCTGAGCTAAACCTA

GCCCCAAACCCGCTCCACCTTACTACCAGACAACCTTAGCCAAACCATTTACCCAAATAAAGTATAGGCG

ATAGAAATTGAAACCTGGCGCAATAGATATAGTACCGCAAGGGAAAGATGAAAAATTATAACCAAGCATA

ATATAGCAAGGACTAACCCCTATACCTTCTGCATAATGAATTAACTAGAAATAACTTTGCAAGGAGAGCC

AAAGCTAAGACCCCCGAAACCAGACGAGCTACCTAAGAACAGCTAAAAGAGCACACCCGTCTATGTAGCA

AAATAGTGGGAAGATTTATAGGTAGAGGCGACAAACCTACCGAGCCTGGTGATAGCTGGTTGTCCAAGAT

AGAATCTTAGTTCAACTTTAAATTTGCCCACAGAACCCTCTAAATCCCCTTGTAAATTTAACTGTTAGTC

CAAAGAGGAACAGCTCTTTGGACACTAGGAAAAAACCTTGTAGAGAGAGTAAAAAATTTAACACCCATAG

TAGGCCTAAAAGCAGCCACCAATTAAGAAAGCGTTCAAGCTCAACACCCACTACCTAAAAAATCCCAAAC

ATATAACTGAACTCCTCACACCCAATTGGACCAATCTATCACCCTATAGAAGAACTAATGTTAGTATAAG

TAACATGAAAACATTCTCCTCCGCATAAGCCTGCGTCAGATTAAAACACTGAACTGACAATTAACAGCCC

AATATCTACAATCAACCAACAAGTCATTATTACCCCCACTGTCAACCCAACACAGGCATGCTCATAAGGA

AAGGTTAAAAAAAGTAAAAGGAACTCGGCAAATCTTACCCCGCCTGTTTACCAAAAACATCACCTCTAGC

ATCACCAGTATTAGAGGCACCGCCTGCCCAGTGACACATGTTTAACGGCCGCGGTACCCTAACCGTGCAA

AGGTAGCATAATCACTTGTTCCTTAAATAGGGACCTGTATGAATGGCTCCACGAGGGTTCAGCTGTCTCT

TACTTTTAACCAGTGAAATTGACCTGCCCGTGAAGAGGCGGGCATGACACAGCAAGACGAGAAGACCCTA

TGGAGCTTTAATTTATTAATGCAAACAGTACCTAACAAACCCACAGGTCCTAAACTACCAAACCTGCATT

AAAAATTTCGGTTGGGGCGACCTCGGAGCAGAACCCAACCTCCGAGCAGTACATGCTAAGACTTCACCAG

TCAAAGCGAACTACTATACTCAATTGATCCAATAACTTGACCAACGGAACAAGTTACCCTAGGGATAACA

GCGCAATCCTATTCTAGAGTCCATATCAACAATAGGGTTTACGACCTCGATGTTGGATCAGGACATCCCG

ATGGTGCAGCCGCTATTAAAGGTTCGTTTGTTCAACGATTAAAGTCCTACGTGATCTGAGTTCAGACCGG

AGTAATCCAGGTCGGTTTCTATCTACNTTCAAATTCCTCCCTGTACGAAAGGACAAGAGAAATAAGGCCT

ACTTCACAAAGCGCCTTCCCCCGTAAATGATATCATCTCAACTTAGTATTATACCCACACCCACCCAAGA

ACAGGGTTTGTTAAGATGGCAGAGCCCGGTAATCGCATAAAACTTAAAACTTTACAGTCAGAGGTTCAAT

TCCTCTTCTTAACAACATACCCATGGCCAACCTCCTACTCCTCATTGTACCCATTCTAATCGCAATGGCA

TTCCTAATGCTTACCGAACGAAAAATTCTAGGCTATATACAACTACGCAAAGGCCCCAACGTTGTAGGCC

CCTACGGGCTACTACAACCCTTCGCTGACGCCATAAAACTCTTCACCAAAGAGCCCCTAAAACCCGCCAC

ATCTACCATCACCCTCTACATCACCGCCCCGACCTTAGCTCTCACCATCGCTCTTCTACTATGAACCCCC

CTCCCCATACCCAACCCCCTGGTCAACCTCAACCTAGGCCTCCTATTTATTCTAGCCACCTCTAGCCTAG

CCGTTTACTCAATCCTCTGATCAGGGTGAGCATCAAACTCAAACTACGCCCTGATCGGCGCACTGCGAGC

AGTAGCCCAAACAATCTCATATGAAGTCACCCTAGCCATCATTCTACTATCAACATTACTAATAAGTGGC

TCCTTTAACCTCTCCACCCTTATCACAACACAAGAACACCTCTGATTACTCCTGCCATCATGACCCTTGG

CCATAATATGATTTATCTCCACACTAGCAGAGACCAACCGAACCCCCTTCGACCTTGCCGAAGGGGAGTC

CGAACTAGTCTCAGGCTTCAACATCGAATACGCCGCAGGCCCCTTCGCCCTATTCTTCATAGCCGAATAC

ACAAACATTATTATAATAAACACCCTCACCACTACAATCTTCCTAGGAACAACATATGACGCACTCTCCC

CTGAACTCTACACAACATATTTTGTCACCAAGACCCTACTTCTAACCTCCCTGTTCTTATGAATTCGAAC

AGCATACCCCCGATTCCGCTACGACCAACTCATACACCTCCTATGAAAAAACTTCCTACCACTCACCCTA

GCATTACTTATATGATATGTCTCCATACCCATTACAATCTCCAGCATTCCCCCTCAAACCTAAGAAATAT

GTCTGATAAAAGAGTTACTTTGATAGAGTAAATAATAGGAGCTTAAACCCCCTTATTTCTAGGACTATGA

GAATCGAACCCATCCCTGAGAATCCAAAATTCTCCGTGCCACCTATCACACCCCATCCTAAAGTAAGGTC

AGCTAAATAAGCTATCGGGCCCATACCCCGAAAATGTTGGTTATACCCTTCCCGTACTAATTAATCCCCT

GGCCCAACCCGTCATCTACTCTACCATCTTTGCAGGCACACTCATCACAGCGCTAAGCTCGCACTGATTT

TTTACCTGAGTAGGCCTAGAAATAAACATGCTAGCTTTTATTCCAGTTCTAACCAAAAAAATAAACCCTC

GTTCCACAGAAGCTGCCATCAAGTATTTCCTCACGCAAGCAACCGCATCCATAATCCTTCTAATAGCTAT

CCTCTTCAACAATATACTCTCCGGACAATGAACCATAACCAATACTACCAATCAATACTCATCATTAATA

ATCATAATGGCTATAGCAATAAAACTAGGAATAGCCCCCTTTCACTTCTGAGTCCCAGAGGTTACCCAAG

GCACCCCTCTGACATCCGGCCTGCTTCTTCTCACATGACAAAAACTAGCCCCCATCTCAATCATATACCA

AATCTCTCCCTCACTAAACGTAAGCCTTCTCCTCACTCTCTCAATCTTATCCATCATAGCAGGCAGTTGA

GGTGGATTAAACCAAACCCAGCTACGCAAAATCTTAGCATACTCCTCAATTACCCACATAGGATGAATAA

TAGCAGTTCTACCGTACAACCCTAACATAACCATTCTTAATTTAACTATTTATATTATCCTAACTACTAC

CGCATTCCTACTACTCAACTTAAACTCCAGCACCACGACCCTACTACTATCTCGCACCTGAAACAAGCTA

ACATGACTAACACCCTTAATTCCATCCACCCTCCTCTCCCTAGGAGGCCTGCCCCCGCTAACCGGCTTTT

TGCCCAAATGGGCCATTATCGAAGAATTCACAAAAAACAATAGCCTCATCATCCCCACCATCATAGCCAC

CATCACCCTCCTTAACCTCTACTTCTACCTACGCCTAATCTACTCCACCTCAATCACACTACTCCCCATA

TCTAACAACGTAAAAATAAAATGACAGTTTGAACATACAAAACCCACCCCATTCCTCCCCACACTCATCG

CCCTCACCACGCTACTCCTACCTATCTCCCCTTTTATACTAATAATCTTATAGAAATTTAGGTTAAATAC

AGACCAAGAGCCTTCAAAGCCCTCAGTAAGTTGCAATACTTAATTTCTGTAACAGCTAAGGACTGCAAAA

CCCCACTCTGCATCAACTGAACGCAAATCAGCCACTTTAATTAAGCTAAGCCCTTACTAGACCAATGGGA

CTTAAACCCACAAACACTTAGTTAACAGCTAAGCACCCTAATCAACTGGCTTCAATCTACTTCTCCCGCC

GCCGGGAAAAAAGGCGGGAGAAGCCCCGGCAGGTTTGAAGCTGCTTCTTCGAATTTGCAATTCAATATGA

AAATCACCTCGGAGCTGGTAAAAAGAGGCCTAACCCCTGTCTTTAGATTTACAGTCCAATGCTTCACTCA

GCCATTTTACCTCACCCCCACTGATGTTCGCCGACCGTTGACTATTCTCTACAAACCACAAAGACATTGG

AACACTATACCTATTATTCGGCGCATGAGCTGGAGTCCTAGGCACAGCTCTAAGCCTCCTTATTCGAGCC

GAGCTGGGCCAGCCAGGCAACCTTCTAGGTAACGACCACATCTACAACGTTATCGTCACAGCCCATGCAT

TTGTAATAATCTTCTTCATAGTAATACCCATCATAATCGGAGGCTTTGGCAACTGACTAGTTCCCCTAAT

AATCGGTGCCCCCGATATGGCGTTTCCCCGCATAAACAACATAAGCTTCTGACTCTTACCTCCCTCTCTC

CTACTCCTGCTCGCATCTGCTATAGTGGAGGCCGGAGCAGGAACAGGTTGAACAGTCTACCCTCCCTTAG

CAGGGAACTACTCCCACCCTGGAGCCTCCGTAGACCTAACCATCTTCTCCTTACACCTAGCAGGTGTCTC

CTCTATCTTAGGGGCCATCAATTTCATCACAACAATTATCAATATAAAACCCCCTGCCATAACCCAATAC

CAAACGCCCCTCTTCGTCTGATCCGTCCTAATCACAGCAGTCCTACTTCTCCTATCTCTCCCAGTCCTAG

CTGCTGGCATCACTATACTACTAACAGACCGCAACCTCAACACCACCTTCTTCGACCCCGCCGGAGGAGG

AGACCCCATTCTATACCAACACCTATTCTGATTTTTCGGTCACCCTGAAGTTTATATTCTTATCCTACCA

GGCTTCGGAATAATCTCCCATATTGTAACTTACTACTCCGGAAAAAAAGAACCATTTGGATACATAGGCA

TGGTCTGAGCTATGATATCAATTGGCTTCCTAGGGTTTATCGTGTGAGCACACCATATATTTACAGTAGG

AATAGACGTAGACACACGAGCATATTTCACCTCCGCTACCATAATCATCGCTATCCCCACCGGCGTCAAA

GTATTTAGCTGACTCGCCACACTCCACGGAAGCAATATGAAATGATCTGCTGCAGTGCTCTGAGCCCTAG

GATTCATCTTTCTTTTCACCGTAGGTGGCCTGACTGGCATTGTATTAGCAAACTCATCACTAGACATCGT

ACTACACGACACGTACTACGTTGTAGCTCACTTCCACTATGTCCTATCAATAGGAGCTGTATTTGCCATC

ATAGGAGGCTTCATTCACTGATTTCCCCTATTCTCAGGCTACACCCTAGACCAAACCTACGCCAAAATCC

ATTTCACTATCATATTCATCGGCGTAAATCTAACTTTCTTCCCACAACACTTTCTCGGCCTATCCGGAAT

GCCCCGACGTTACTCGGACTACCCCGATGCATACACCACATGAAACATCCTATCATCTGTAGGCTCATTC

ATTTCTCTAACAGCAGTAATATTAATAATTTTCATGATTTGAGAAGCCTTCGCTTCGAAGCGAAAAGTCC

TAATAGTAGAAGAACCCTCCATAAACCTGGAGTGACTATATGGATGCCCCCCACCCTACCACACATTCGA

AGAACCCGTATACATAAAATCTAGACAAAAAAGGAAGGAATCGAACCCCCCAAAGCTGGTTTCAAGCCAA

CCCCATGGCCTCCATGACTTTTTCAAAAAGGTATTAGAAAAACCATTTCATAACTTTGTCAAAGTTAAAT

TATAGGCTAAATCCTATATATCTTAATGGCACATGCAGCGCAAGTAGGTCTACAAGACGCTACTTCCCCT

ATCATAGAAGAGCTTATCACCTTTCATGATCACGCCCTCATAATCATTTTCCTTATCTGCTTCCTAGTCC

TGTATGCCCTTTTCCTAACACTCACAACAAAACTAACTAATACTAACATCTCAGACGCTCAGGAAATAGA

AACCGTCTGAACTATCCTGCCCGCCATCATCCTAGTCCTCATCGCCCTCCCATCCCTACGCATCCTTTAC

ATAACAGACGAGGTCAACGATCCCTCCCTTACCATCAAATCAATTGGCCACCAATGGTACTGAACCTACG

AGTACACCGACTACGGCGGACTAATCTTCAACTCCTACATACTTCCCCCATTATTCCTAGAACCAGGCGA

CCTGCGACTCCTTGACGTTGACAATCGAGTAGTACTCCCGATTGAAGCCCCCATTCGTATAATAATTACA

TCACAAGACGTCTTGCACTCATGAGCTGTCCCCACATTAGGCTTAAAAACAGATGCAATTCCCGGACGTC

TAAACCAAACCACTTTCACCGCTACACGACCGGGGGTATACTACGGTCAATGCTCTGAAATCTGTGGAGC

AAACCACAGTTTCATGCCCATCGTCCTAGAATTAATTCCCCTAAAAATCTTTGAAATAGGGCCCGTATTT

ACCCTATAGCACCCCCTCTAGAGCCCACTGTAAAGCTAACTTAGCATTAACCTTTTAAGTTAAAGATTAA

GAGAACCAACACCTCTTTACAGTGAAATGCCCCAACTAAATACTACCGTATGGCCCACCATAATTACCCC

CATACTCCTTACACTATTCCTCATCACCCAACTAAAAATATTAAACACAAACTACCACCTACCTCCCTCA

CCAAAGCCCATAAAAATAAAAAATTATAACAAACCCTGAGAACCAAAATGAACGAAAATCTGTTCGCTTC

ATTCATTGCCCCCACAATCCTAGGCCTACCCGCCGCAGTACTGATCATTCTATTTCCCCCTCTATTGATC

CCCACCTCCAAATATCTCATCAACAACCGACTAATCACCACCCAACAATGACTAATCAAACTAACCTCAA

AACAAATGATAACCATACACAACACTAAAGGACGAACCTGATCTCTTATACTAGTATCCTTAATCATTTT

TATTGCCACAACTAACCTCCTCGGACTCCTGCCTCACTCATTTACACCAACCACCCAACTATCTATAAAC

CTAGCCATGGCCATCCCCTTATGAGCGGGCGCAGTGATTATAGGCTTTCGCTCTAAGATTAAAAATGCCC

TAGCCCACTTCTTACCACAAGGCACACCTACACCCCTTATCCCCATACTAGTTATTATCGAAACCATCAG

CCTACTCATTCAACCAATAGCCCTGGCCGTACGCCTAACCGCTAACATTACTGCAGGCCACCTACTCATG

CACCTAATTGGAAGCGCCACCCTAGCAATATCAACCATTAACCTTCCCTCTACACTTATCATCTTCACAA

TTCTAATTCTACTAACTATCCTAGAAATCGCTGTCGCCTTAATCCAAGCCTACGTTTTCACACTTCTAGT

AAGCCTCTACCTGCACGACAACACATAATGACCCACCAATCACATGCCTATCATATAGTAAAACCCAGCC

CATGACCCCTAACAGGGGCCCTCTCAGCCCTCCTAATGACCTCCGGCCTAGCCATGTGATTTCACTTCCA

CTCCATAACGCTCCTCATACTAGGCCTACTAACCAACACACTAACCATATACCAATGATGGCGCGATGTA

ACACGAGAAAGCACATACCAAGGCCACCACACACCACCTGTCCAAAAAGGCCTTCGATACGGGATAATCC

TATTTATTACCTCAGAAGTTTTTTTCTTCGCAGGATTTTTCTGAGCCTTTTACCACTCCAGCCTAGCCCC

TACCCCCCAATTAGGAGGGCACTGGCCCCCAACAGGCATCACCCCGCTAAATCCCCTAGAAGTCCCACTC

CTAAACACATCCGTATTACTCGCATCAGGAGTATCAATCACCTGAGCTCACCATAGTCTAATAGAAAACA

ACCGAAACCAAATAATTCAAGCACTGCTTATTACAATTTTACTGGGTCTCTATTTTACCCTCCTACAAGC

CTCAGAGTACTTCGAGTCTCCCTTCACCATTTCCGACGGCATCTACGGCTCAACATTTTTTGTAGCCACA

GGCTTCCACGGACTTCACGTCATTATTGGCTCAACTTTCCTCACTATCTGCTTCATCCGCCAACTAATAT

TTCACTTTACATCCAAACATCACTTTGGCTTCGAAGCCGCCGCCTGATACTGGCATTTTGTAGATGTGGT

TTGACTATTTCTGTATGTCTCCATCTATTGATGAGGGTCTTACTCTTTTAGTATAAATAGTACCGTTAAC

TTCCAATTAACTAGTTTTGACAACATTCAAAAAAGAGTAATAAACTTCGCCTTAATTTTAATAATCAACA

CCCTCCTAGCCTTACTACTAATAATTATTACATTTTGACTACCACAACTCAACGGCTACATAGAAAAATC

CACCCCTTACGAGTGCGGCTTCGACCCTATATCCCCCGCCCGCGTCCCTTTCTCCATAAAATTCTTCTTA

GTAGCTATCACCTTCTTATTATTTGATCTAGAAATTGCCCTCCTTTTACCCCTACCATGAGCCCTACAAA

CAACTAACCTGCCACTAATAGTTATGTCATCCCTCTTATTAATCATCATCCTAGCCCTAAGTCTGGCCTA

TGAGTGACTACAAAAAGGATTAGACTGAACCGAATTGGTATATAGTTTAAACAAAACGAATGATTTCGAC

TCATTAAATTATGATAATCATATTTACCAAATGCCCCTCATTTACATAAATATTATACTAGCATTTACCA

TCTCACTTCTAGGAATACTAGTATATCGCTCACACCTCATATCCTCCCTACTATGCCTAGAAGGAATAAT

ACTATCGCTGTTCATTATAGCTACTCTCATAACCCTCAACACCCACTCCCTCTTAGCCAATATTGTGCCT

ATTGCCATACTAGTCTTTGCCGCCTGCGAAGCAGCGGTGGGCCTAGCCCTACTAGTCTCAATCTCCAACA

CATATGGCCTAGACTACGTACATAACCTAAACCTACTCCAATGCTAAAACTAATCGTCCCAACAATTATA

TTACTACCACTGACATGACTTTCCAAAAAACACATAATTTGAATCAACACAACCACCCACAGCCTAATTA

TTAGCATCATCCCTCTACTATTTTTTAACCAAATCAACAACAACCTATTTAGCTGTTCCCCAACCTTTTC

CTCCGACCCCCTAACAACCCCCCTCCTAATACTAACTACCTGACTCCTACCCCTCACAATCATGGCAAGC

CAACGCCACTTATCCAGTGAACCACTATCACGAAAAAAACTCTACCTCTCTATACTAATCTCCCTACAAA

TCTCCTTAATTATAACATTCACAGCCACAGAACTAATCATATTTTATATCTTCTTCGAAACCACACTTAT

CCCCACCTTGGCTATCATCACCCGATGAGGCAACCAGCCAGAACGCCTGAACGCAGGCACATACTTCCTA

TTCTACACCCTAGTAGGCTCCCTTCCCCTACTCATCGCACTAATTTACACTCACAACACCCTAGGCTCAC

TAAACATTCTACTACTCACTCTCACTGCCCAAGAACTATCAAACTCCTGAGCCAACAACTTAATATGACT

AGCTTACACAATAGCTTTTATAGTAAAGATACCTCTTTACGGACTCCACTTATGACTCCCTAAAGCCCAT

GTCGAAGCCCCCATCGCTGGGTCAATAGTACTTGCCGCAGTACTCTTAAAACTAGGCGGCTATGGTATAA

TACGCCTCACACTCATTCTCAACCCCCTGACAAAACACATAGCCTACCCCTTCCTTGTACTATCCCTATG

AGGCATAATTATAACAAGCTCCATCTGCCTACGACAAACAGACCTAAAATCGCTCATTGCATACTCTTCA

ATCAGCCACATAGCCCTCGTAGTAACAGCCATTCTCATCCAAACCCCCTGAAGCTTCACCGGCGCAGTCA

TTCTCATAATCGCCCACGGACTTACATCCTCATTACTATTCTGCCTAGCAAACTCAAACTACGAACGCAC

TCACAGTCGCATCATAATCCTCTCTCAAGGACTTCAAACTCTACTCCCACTAATAGCTTTTTGATGACTT

CTAGCAAGCCTCGCTAACCTCGCCTTACCCCCCACTATTAACCTACTGGGAGAACTCTCTGTGCTAGTAA

CCACGTTCTCCTGATCAAATATCACTCTCCTACTTACAGGACTCAACATACTAGTCACAGCCCTATACTC

CCTCTACATATTTACCACAACACAATGGGGCTCACTCACCCACCACATTAACAACATAAAACCCTCATTC

ACACGAGAAAACACCCTCATGTTCATACACCTATCCCCCATTCTCCTCCTATCCCTCAACCCCGACATCA

TTACCGGGTTTTCCTCTTGTAAATATAGTTTAACCAAAACATCAGATTGTGAATCTGACAACAGAGGCTT

ACGACCCCTTATTTACCGAGAAAGCTCACAAGAACTGCTAACTCATGCCTCCATGTCTAACAACATGGCT

TTCTCAACTTTTAAAGGATAACAGCTATCCATTGGTCTTAGGCCCCAAAAATTTTGGTGCAACTCCAAAT

AAAAGTAATAACCATGCACACTACTATAACCACCCTAACCCTGACTTCCCTAATTCCCCCCATCCTTACC

ACCCTCGTTAACCCTAACAAAAAAAACTCATACCCCCATTATGTAAAATCCATTGTCGCATCCACCTTTA

TTATCAGTCTCTTCCCCACAACAATATTCATGTGCCTAGACCAAGAAGTTATTATCTCGAACTGACACTG

AGCCACAACCCAAACAACCCAGCTCTCCCTAAGCTTCAAACTAGACTACTTCTCCATAATATTCATCCCT

GTAGCATTGTTCGTTACATGGTCCATCATAGAATTCTCACTGTGATATATAAACTCAGACCCAAACATTA

ATCAGTTCTTCAAATATCTACTCATCTTCCTAATTACCATACTAATCTTAGTTACCGCTAACAACCTATT

CCAACTGTTCATCGGCTGAGAGGGCGTAGGAATTATATCCTTCTTGCTCATCAGTTGATGATACGCCCGA

GCAGATGCCAACACAGCAGCCATTCAAGCAATCCTATACAACCGTATCGGCGATATCGGTTTCATCCTCG

CCTTAGCATGATTTATCCTACACTCCAACTCATGAGACCCACAACAAATAGCCCTTCTAAACGCTAATCC

AAGCCTCACCCCACTACTAGGCCTCCTCCTAGCAGCAGCAGGCAAATCAGCCCAATTAGGTCTCCACCCC

TGACTCCCCTCAGCCATAGAAGGCCCCACCCCAGTCTCAGCCCTACTCCACTCAAGCACTATAGTTGTAG

CAGGAATCTTCTTACTCATCCGCTTCCACCCCCTAGCAGAAAATAGCCCACTAATCCAAACTCTAACACT

ATGCTTAGGCGCTATCACCACTCTGTTCGCAGCAGTCTGCGCCCTTACACAAAATGACATCAAAAAAATC

GTAGCCTTCTCCACTTCAAGTCAACTAGGACTCATAATAGTTACAATCGGCATCAACCAACCACACCTAG

CATTCCTGCACATCTGTACCCACGCCTTCTTCAAAGCCATACTATTTATGTGCTCCGGGTCCATCATCCA

CAACCTTAACAATGAACAAGATATTCGAAAAATAGGAGGACTACTCAAAACCATACCTCTCACTTCAACC

TCCCTCACCATTGGCAGCCTAGCATTAGCAGGAATACCTTTCCTCACAGGTTTCTACTCCAAAGACCACA

TCATCGAAACCGCAAACATATCATACACAAACGCCTGAGCCCTATCTATTACTCTCATCGCTACCTCCCT

GACAAGCGCCTATAGCACTCGAATAATTCTTCTCACCCTAACAGGTCAACCTCGCTTCCCCACCCTTACT

AACATTAACGAAAATAACCCCACCCTACTAAACCCCATTAAACGCCTGGCAGCCGGAAGCCTATTCGCAG

GATTTCTCATTACTAACAACATTTCCCCCGCATCCCCCTTCCAAACAACAATCCCCCTCTACCTAAAACT

CACAGCCCTCGCTGTCACTTTCCTAGGACTTCTAACAGCCCTAGACCTCAACTACCTAACCAACAAACTT

AAAATAAAATCCCCACTATGCACATTTTATTTCTCCAACATACTCGGATTCTACCCTAGCATCACACACC

GCACAATCCCCTATCTAGGCCTTCTTACGAGCCAAAACCTGCCCCTACTCCTCCTAGACCTAACCTGACT

AGAAAAGCTATTGCCTAAAACAATTTCACAGCACCAAATCTCCACCTCCATCATCACCTCAACCCAAAAA

GGCATAATTAAACTTTACTTCCTCTCTTTCTTCTTCCCACTCATCCTAACCCTACTCCTAATCACATAAC

CTATTCCCCCGAGCAATCTCAATTACAATATATACACCAACAAACAATGTTCAACCAGTAACTACTACTA

ATCAACGCCCATAATCATACAAAGCCCCCGCACCAATAGGATCCTCCCGAATCAACCCTGACCCCTCTCC

TTCATAAATTATTCAGCTTCCTACACTATTAAAGTTTACCACAACCACCACCCCATCATACTCTTTCACC

CACAGCACCAATCCTACCTCCATCGCTAACCCCACTAAAACACTCACCAAGACCTCAACCCCTGACCCCC

ATGCCTCAGGATACTCCTCAATAGCCATCGCTGTAGTATATCCAAAGACAACCATCATTCCCCCTAAATA

AATTAAAAAAACTATTAAACCCATATAACCTCCCCCAAAATTCAGAATAATAACACACCCGACCACACCG

CTAACAATCAATACTAAACCCCCATAAATAGGAGAAGGCTTAGAAGAAAACCCCACAAACCCCATTACTA

AACCCACACTCAACAGAAACAAAGCATACATCATTATTCTCGCACGGACTACAACCACGACCAATGATAT

GAAAAACCATCGTTGTATTTCAACTACAAGAACACCAATGACCCCAATACGCAAAATTAACCCCCTAATA

AAATTAATTAACCACTCATTCATCGACCTCCCCACCCCATCCAACATCTCCGCATGATGAAACTTCGGCT

CACTCCTTGGCGCCTGCCTGATCCTCCAAATCACCACAGGACTATTCCTAGCCATGCACTACTCACCAGA

CGCCTCAACCGCCTTTTCATCAATCGCCCACATCACTCGAGACGTAAATTATGGCTGAATCATCCGCTAC

CTTCACGCCAATGGCGCCTCAATATTCTTTATCTGCCTCTTCCTACACATCGGGCGAGGCCTATATTACG

GATCATTTCTCTACTCAGAAACCTGAAACATCGGCATTATCCTCCTGCTTGCAACTATAGCAACAGCCTT

CATAGGCTATGTCCTCCCGTGAGGCCAAATATCATTCTGAGGGGCCACAGTAATTACAAACTTACTATCC

GCCATCCCATACATTGGGACAGACCTAGTTCAATGAATCTGAGGAGGCTACTCAGTAGACAGTCCCACCC

TCACACGATTCTTTACCTTTCACTTCATCTTGCCCTTCATTATTGCAGCCCTAGCAGCACTCCACCTCCT

ATTCTTGCACGAAACGGGATCAAACAACCCCCTAGGAATCACCTCCCATTCCGATAAAATCACCTTCCAC

CCTTACTACACAATCAAAGACGCCCTCGGCTTACTTCTCTTCCTTCTCTCCTTAATGACATTAACACTAT

TCTCACCAGACCTCCTAGGCGACCCAGACAATTATACCCTAGCCAACCCCTTAAACACCCCTCCCCACAT

CAAGCCCGAATGATATTTCCTATTCGCCTACACAATTCTCCGATCCGTCCCTAACAAACTAGGAGGCGTC

CTTGCCCTATTACTATCCATCCTCATCCTAGCAATAATCCCCATCCTCCATATATCCAAACAACAAAGCA

TAATATTTCGCCCACTAAGCCAATCACTTTATTGACTCCTAGCCGCAGACCTCCTCGTTCTAACCTGAAT

CGGAGGACAACCAGTAAGCTACCCTTTTACCATCATTGGACAAGTAGCATCCGTACTATACTTCACAACA

ATCCTAATCCTAATACCAACTATCTCCCTAATTGAAAACAAAATACTCAAATGGGCCTGTCCTTGTAGTA

TAAACTAATACACCAGTCTTGTAAACCGGAGATGAAAACCTTTTTCCAAGGACAAATCAGAGAAAAAGTC

TTTAACTCCACCATTAGCACCCAAAGCTAAGATTCTAATTTAAACTATTCTCTGTTCTTTCATGGGGAAG

CAGATTTGGGTACCACCCAAGTATTGACTCACCCATCAACAACCGCTATGTATTTCGTACATTACTGCCA

GCCACCATGAATATTGTACGGTACCATAAATACTTGACCACCTGTAGTACATAAAAACCCAATCCACATC

AACCCCCCCCCCCCATGCTTACAAGCAAGTACAGCAACCAACCCTCAACTATCACACATCAACTGCAGCT

CCAAAGCCACCTCTCACCCACTAGGATACCAACAAACCTACCCACCCTTAACAGTACATAGTACATAAAG

CCATTTACCGTACATAGCACATTACAGTCAAATCCCTTCTCGTCCCCATGGATGACCCCCCTCAGATAGG

GGTCCCTTGACCACCATCCTCCGTGAAATCAATATCCCGCACAAGAGTGCTACTCTCCTCGCTCCGGGCC

CATAACACTTGGGGGTAGCTAAAGTGAACTGTATCCGACATCTGGTTCCTACTTCAGGGCCATAAAGCCT

AAATAGCCCACACGTTCCCCTTAAATAAGACATCACGATG

>MT135510.1 Homo sapiens isolate HO23 mitochondrion, complete genome

GATCACAGGTCTATCACCCTATTAACCACTCACGGGAGCTCTCCATGCATTTGGTATTTTCGTCTGGGGG

GTGTGCACGCGATAGCATTGCGAGACGCTGGAGCCGGAGCACCCTATGTCGCAGTATCTGTCTTTGATTC

CTGCCCCATCCTATTATTTATCGCACCTACGTTCAATATTACAGGCGAACATACTTACTAAAGTGTGTTA

ATTAATTAATGCTTGTAGGACATAATAATAACAATTGAATGTCTGCACAGCCGCTTTCCACACAGACATC

ATAACAAAAAATTTCCACCAAACCCCCCCCCTCCCCCCGCCTCTGGCCACAGCACTTAAACACATCTCTG

CCAAACCCCAAAAACAAAGAACCCTAACACCAGCCTAACCAGATTTCAAATTTTATCTTTTGGCGGTATG

CACTTTTAACAGTCACCCCCCAACTAACACATTATTTTCCCCTCCCACTCCCATACTACTAATCTCATCA

ATACAACCCCCGCCCATCCTACCCAGCACACACACCGCTGCTAACCCCATACCCCGAACCAACCAAACCC

CAAAGACACCCCCCACAGTTTATGTAGCTTACCTCCTCAAAGCAATACACTGAAAATGTTTAGACGGGCT

CACATCACCCCATAAACAAATAGGTTTGGTCCTAGCCTTTCTATTAGCTCTTAGTAAGATTACACATGCA

AGCATCCCCGTTCCAGTGAGTTCACCCTCTAAATCACCACGATCAAAAGGGACAAGCATCAAGCACGCAG

CAATGCAGCTCAAAACGCTTAGCCTAGCCACACCCCCACGGGAAACAGCAGTGATTAACCTTTAGCAATA

AACGAAAGTTTAACTAAGCTATACTAACCCCAGGGTTGGTCAATTTCGTGCCAGCCACCGCGGTCACACG

ATTAACCCAAGTCAATAGAAGCCGGCGTAAAGAGTGTTTTAGATCACCCCCTCCCCAATAAAGCTAAAAC

TCACCTGAGTTGTAAAAAACTCCAGTTGACACAAAATAGACTACGAAAGTGGCTTTAACATATCTGAACA

CACAATAGCTAAGACCCAAACTGGGATTAGATACCCCACTATGCTTAGCCCTAAACCTCAACAGTTAAAT

CAACAAAACTGCTCGCCAGAACACTACGAGCCACAGCTTAAAACTCAAAGGACCTGGCGGTGCTTCATAT

CCCTCTAGAGGAGCCTGTTCTGTAATCGATAAACCCCGATCAACCTCACCACCTCTTGCTCAGCCTATAT

ACCGCCATCTTCAGCAAACCCTGATGAAGGCTACAAAGTAAGCGCAAGTACCCACGTAAAGACGTTAGGT

CAAGGTGTAGCCCATGAGGTGGCAAGAAATGGGCTACATTTTCTACCCCAGAAAACTACGATAGCCCTTA

TGAAACTTAAGGGTCGAAGGTGGATTTAGCAGTAAACTGAGAGTAGAGTGCTTAGTTGAACAGGGCCCTG

AAGCGCGTACACACCGCCCGTCACCCTCCTCAAGTATACTTCAAAGGACATTTAACTAAAACCCCTACGC

ATTTATATAGAGGAGACAAGTCGTAACATGGTAAGTGTACTGGAAAGTGCACTTGGACGAACCAGAGTGT

AGCTTAACACAAAGCACCCAACTTACACTTAGGAGATTTCAACTTAACTTGACCGCTCTGAGCTAAACCT

AGCCCCAAACCCACTCCACCTTACTACCAGACAACCTTAGCCAAACCATTTACCCAAATAAAGTATAGGC

GATAGAAATTGAAACCTGGCGCAATAGATATAGTACCGCAAGGGAAAGATGAAAAATTATAACCAAGCAT

AATATAGCAAGGACTAACCCCTATACCTTCTGCATAATGAATTAACTAGAAATAACTTTGCAAGGAGAGC

CAAAGCTAAGACCCCCGAAACCAGACGAGCTACCTAAGAACAGCTAAAAGAGCACACCCGTCTATGTAGC

AAAATAGTGGGAAGATTTATAGGTAGAGGCGACAAACCTACCGAGCCTGGTGATAGCTGGTTGTCCAAGA

TAGAATCTTAGTTCAACTTTAAATTTGCCCACAGAACCCTCTAAATCCCCTTGTAAATTTAACTGTTAGT

CCAAAGAGGAACAGCTCTTTGGACACTAGGAAAAAACCTTGTAGAGAGAGTAAAAAATTTAACACCCATA

GTAGGCCTAAAAGCAGCCACCAATTAAGAAAGCGTTCAAGCTCAACACCCACTACCTAAAAAATCCCAAA

CATATAACTGAACTCCTCACACCCAATTGGACCAATCTATCACCCTATAGAAGAACTAATGTTAGTATAA

GTAACATGAAAACATTCTCCTCCGCATAAGCCTGCGTCAGATTAAAACACTGAACTGACAATTAACAGCC

CAATATCTACAATCAACCAACAAGTCATTATTACCCTCACTGTCAACCCAACACAGGCATGCTCATAAGG

AAAGGTTAAAAAAAGTAAAAGGAACTCGGCAAATCTTACCCCGCCTGTTTACCAAAAACATCACCTCTAG

CATCACCAGTATTAGAGGCACCGCCTGCCCAGTGACACATGTTTAACGGCCGCGGTACCCTAACCGTGCA

AAGGTAGCATAATCACTTGTTCCTTAAATAGGGACCTGTATGAATGGCTCCACGAGGGTTCAGCTGTCTC

TTACTTTTAACCAGTGAAATTGACCTGCCCGTGAAGAGGCGGGCATGACACAGCAAGACGAGAAGACCCT

ATGGAGCTTTAATTTATTAATGCAAACAGTACCTAACAAACCCACAGGTCCTAAACTACCAAACCTGCAT

TAAAAATTTCGGTTGGGGCGACCTCGGAGCAGAACCCAACCTCCGAGCAGTACATGCTAAGACTTCACCA

GTCAAAGCGAACTACTATACTCAATTGATCCAATAACTTGACCAACGGAACAAGTTACCCTAGGGATAAC

AGCGCAATCCTATTCTAGAGTCCATATCAACAATAGGGTTTACGACCTCGATGTTGGATCAGGACATCCC

GATGGTGCAGCCGCTATTAAAGGTTCGTTTGTTCAACGATTAAAGTCCTACGTGATCTGAGTTCAGACCG

GAGTAATCCAGGTCGGTTTCTATCTACNTTCAAATTCCTCCCTGTACGAAAGGACAAGAGAAATAAGGCC

TACTTCACAAAGCGCCTTCCCCCGTAAATGATATCATCTCAACTTAGTATTATACCCACACCCACCCAAG

AACAGGGTTTGTTAAGATGGCAGAGCCCGGTAATCGCATAAAACTTAAAACTTTACAGTCAGAGGTTCAA

TTCCTCTTCTTAACAACATACCCATGGCCAACCTCCTACTCCTCATTGTACCCATTCTAATCGCAATGGC

ATTCCTAATGCTTACCGAACGAAAAATTCTAGGCTATATACAACTACGCAAAGGCCCCAACGTTGTAGGC

CCCTACGGGCTACTACAACCCTTCGCTGACGCCATAAAACTCTTCACCAAAGAGCCCCTAAAACCCGCCA

CATCTACCATCACCCTCTACATCACCGCCCCGACCTTAGCTCTCACCATCGCTCTTCTACTATGAACCCC

CCTCCCCATACCCAACCCCCTGGTCAACCTCAACCTAGGCCTCCTATTTATTCTAGCCACCTCTAGCCTA

GCCGTTTACTCAATCCTCTGATCAGGGTGAGCATCAAACTCAAACTACGCCCTGATCGGCGCACTGCGAG

CAGTAGCCCAAACAATCTCATATGAAGTCACCCTAGCCATCATTCTACTATCAACATTACTAATAAGTGG

CTCCTTTAACCTCTCCACCCTTATCACAACACAAGAACACCTCTGATTACTCCTGCCATCATGACCCTTG

GCCATAATATGATTTATCTCCACACTAGCAGAGACCAACCGAACCCCCTTCGACCTTGCCGAAGGGGAGT

CCGAACTAGTCTCAGGCTTCAACATCGAATACGCCGCAGGCCCCTTCGCCCTATTCTTCATAGCCGAATA

CACAAACATTATTATAATAAACACCCTCACCACTACAATCTTCCTAGGAACAACATATGACGCACTCTCC

CCTGAACTCTACACAACATATTTTGTCACCAAGACCCTACTTCTAACCTCCCTGTTCTTATGAATTCGAA

CAGCATACCCCCGATTCCGCTACGACCAACTCATACACCTCCTATGAAAAAACTTCCTACCACTCACCCT

AGCATTACTTATATGATATGTCTCCATACCCATTACAATCTCCAGCATTCCCCCTCAAACCTAAGAAATA

TGTCTGATAAAAGAGTTACTTTGATAGAGTAAATAATAGGAGCTTAAACCCCCTTATTTCTAGGACTATG

AGAATCGAACCCATCCCTGAGAATCCAAAATTCTCCGTGCCACCTATCACACCCCATCCTAAAGTAAGGT

CAGCTAAATAAGCTATCGGGCCCATACCCCGAAAATGTTGGTTATACCCTTCCCGTACTAATTAATCCCC

TGGCCCAACCCGTCATCTACTCTACCATCTTTGCAGGCACACTCATCACAGCGCTAAGCTCGCACTGATT

TTTTACCTGAGTAGGCCTAGAAATAAACATGCTAGCTTTTATTCCAGTTCTAACCAAAAAAATAAACCCT

CGTTCCACAGAAGCTGCCATCAAGTATTTCCTCACGCAAGCAACCGCATCCATAATCCTTCTAATAGCTA

TCCTCTTCAACAATATACTCTCCGGACAATGAACCATAACCAATACTACCAATCAATACTCATCATTAAT

AATCATAATGGCTATAGCAATAAAACTAGGAATAGCCCCCTTTCACTTCTGAGTCCCAGAGGTTACCCAA

GGCACCCCTCTGACATCCGGCCTGCTTCTTCTCACATGACAAAAACTAGCCCCCATCTCAATCATATACC

AAATCTCTCCCTCACTAAACGTAAGCCTTCTCCTCACTCTCTCAATCTTATCCATCATAGCAGGCAGTTG

AGGTGGATTAAACCAAACCCAGCTACGCAAAATCTTAGCATACTCCTCAATTACCCACATAGGATGAATA

ATAGCAGTTCTACCGTACAACCCTAACATAACCATTCTTAATTTAACTATTTATATTATCCTAACTACTA

CCGCATTCCTACTACTCAACTTAAACTCCAGCACCACGACCCTACTACTATCTCGCACCTGAAACAAGCT

AACATGACTAACACCCTTAATTCCATCCACCCTCCTCTCCCTAGGAGGCCTGCCCCCGCTAACCGGCTTT

TTGCCCAAATGGGCCATTATCGAAGAATTCACAAAAAACAATAGCCTCATCATCCCCACCATCATAGCCA

CCATCACCCTCCTTAACCTCTACTTCTACCTACGCCTAATCTACTCCACCTCAATCACACTACTCCCCAT

ATCTAACAACGTAAAAATAAAATGACAGTTTGAACATACAAAACCCACCCCATTCCTCCCCACACTCATC

GCCCTCACCACGCTACTCCTACCTATCTCCCCTTTTATACTAATAATCTTATAGAAATTTAGGTTAAATA

CAGACCAAGAGCCTTCAAAGCCCTCAGTAAGTTGCAATACTTAATTTCTGTAACAGCTAAGGACTGCAAA

ACCCCACTCTGCATCAACTGAACGCAAATCAGCCACTTTAATTAAGCTAAGCCCTTACTAGACCAATGGG

ACTTAAACCCACAAACACTTAGTTAACAGCTAAGCACCCTAATCAACTGGCTTCAATCTACTTCTCCCGC

CGCCGGGAAAAAAGGCGGGAGAAGCCCCGGCAGGTTTGAAGCTGCTTCTTCGAATTTGCAATTCAATATG

AAAATCACCTCGGAGCTGGTAAAAAGAGGCCTAACCCCTGTCTTTAGATTTACAGTCCAATGCTTCACTC

AGCCATTTTACCTCACCCCCACTGATGTTCGCCGACCGTTGACTATTCTCTACAAACCACAAAGACATTG

GAACACTATACCTATTATTCGGCGCATGAGCTGGAGTCCTAGGCACAGCTCTAAGCCTCCTTATTCGAGC

CGAGCTGGGCCAGCCAGGCAACCTTCTAGGTAACGACCACATCTACAACGTTATCGTCACAGCCCATGCA

TTTGTAATAATCTTCTTCATAGTAATACCCATCATAATCGGAGGCTTTGGCAACTGACTAGTTCCCCTAA

TAATCGGTGCCCCCGATATGGCGTTTCCCCGCATAAACAACATAAGCTTCTGACTCTTACCTCCCTCTCT

CCTACTCCTGCTCGCATCTGCTATAGTGGAGGCCGGAGCAGGAACAGGTTGAACAGTCTACCCTCCCTTA

GCAGGGAACTACTCCCACCCTGGAGCCTCCGTAGACCTAACCATCTTCTCCTTACACCTAGCAGGTGTCT

CCTCTATCTTAGGGGCCATCAATTTCATCACAACAATTATCAATATAAAACCCCCTGCCATAACCCAATA

CCAAACGCCCCTCTTCGTCTGATCCGTCCTAATCACAGCAGTCCTACTTCTCCTATCTCTCCCAGTCCTA

GCTGCTGGCATCACTATACTACTAACAGACCGCAACCTCAACACCACCTTCTTCGACCCCGCCGGAGGAG

GAGACCCCATTCTATACCAACACCTATTCTGATTTTTCGGTCACCCTGAAGTTTATATTCTTATCCTACC

AGGCTTCGGAATAATCTCCCATATTGTAACTTACTACTCCGGAAAAAAAGAACCATTTGGATACATAGGC

ATGGTCTGAGCTATGATATCAATTGGCTTCCTAGGGTTTATCGTGTGAGCACACCATATATTTACAGTAG

GAATAGACGTAGACACACGAGCATATTTCACCTCCGCTACCATAATCATCGCTATCCCCACCGGCGTCAA

AGTATTTAGCTGACTCGCCACACTCCACGGAAGCAATATGAAATGATCTGCTGCAGTGCTCTGAGCCCTA

GGATTCATCTTTCTTTTCACCGTAGGTGGCCTGACTGGCATTGTATTAGCAAACTCATCACTAGACATCG

TACTACACGACACGTACTACGTTGTAGCTCACTTCCACTATGTCCTATCAATAGGAGCTGTATTTGCCAT

CATAGGAGGCTTCATTCACTGATTTCCCCTATTCTCAGGCTACACCCTAGACCAAACCTACGCCAAAATC

CATTTCACTATCATATTCATCGGCGTAAATCTAACTTTCTTCCCACAACACTTTCTCGGCCTATCCGGAA

TGCCCCGACGTTACTCGGACTACCCCGATGCATACACCACATGAAACATCCTATCATCTGTAGGCTCATT

CATTTCTCTAACAGCAGTAATATTAATAATTTTCATGATTTGAGAAGCCTTCGCTTCGAAGCGAAAAGTC

CTAATAGTAGAAGAACCCTCCATAAACCTGGAGTGACTATATGGATGCCCCCCACCCTACCACACATTCG

AAGAACCCGTATACATAAAATCTAGACAAAAAAGGAAGGAATCGAACCCCCCAAAGCTGGTTTCAAGCCA

ACCCCATGGCCTCCATGACTTTTTCAAAAAGGTATTAGAAAAACCATTTCATAACTTTGTCAAAGTTAAA

TTATAGGCTAAATCCTATATATCTTAATGGCACATGCAGCGCAAGTAGGTCTACAAGACGCTACTTCCCC

TATCATAGAAGAGCTTATCACCTTTCATGATCACGCCCTCATAATCATTTTCCTTATCTGCTTCCTAGTC

CTGTATGCCCTTTTCCTAACACTCACAACAAAACTAACTAATACTAACATCTCAGACGCTCAGGAAATAG

AAACCGTCTGAACTATCCTGCCCGCCATCATCCTAGTCCTCATCGCCCTCCCATCCCTACGCATCCTTTA

CATAACAGACGAGGTCAACGATCCCTCCCTTACCATCAAATCAATTGGCCACCAATGGTACTGAACCTAC

GAGTACACCGACTACGGCGGACTAATCTTCAACTCCTACATACTTCCCCCATTATTCCTAGAACCAGGCG

ACCTGCGACTCCTTGACGTTGACAATCGAGTAGTACTCCCGATTGAAGCCCCCATTCGTATAATAATTAC

ATCACAAGACGTCTTGCACTCATGAGCTGTCCCCACATTAGGCTTAAAAACAGATGCAATTCCCGGACGT

CTAAACCAAACCACTTTCACCGCTACACGACCGGGGGTATACTACGGTCAATGCTCTGAAATCTGTGGAG

CAAACCACAGTTTCATGCCCATCGTCCTAGAATTAATTCCCCTAAAAATCTTTGAAATAGGGCCCGTATT

TACCCTATAGCACCCCCTCTAGAGCCCACTGTAAAGCTAACTTAGCATTAACCTTTTAAGTTAAAGATTA

AGAGAACCAACACCTCTTTACAGTGAAATGCCCCAACTAAATACTACCGTATGGCCCACCATAATTACCC

CCATACTCCTTACACTATTCCTCATCACCCAACTAAAAATATTAAACACAAACTACCACCTACCTCCCTC

ACCAAAGCCCATAAAAATAAAAAATTATAACAAACCCTGAGAACCAAAATGAACGAAAATCTGTTCGCTT

CATTCATTGCCCCCACAATCCTAGGCCTACCCGCCGCAGTACTGATCATTCTATTTCCCCCTCTATTGAT

CCCCACCTCCAAATATCTCATCAACAACCGACTAATCACCACCCAACAATGACTAATCAAACTAACCTCA

AAACAAATGATAACCATACACAACACTAAAGGACGAACCTGATCTCTTATACTAGTATCCTTAATCATTT

TTATTGCCACAACTAACCTCCTCGGACTCCTGCCTCACTCATTTACACCAACCACCCAACTATCTATAAA

CCTAGCCATGGCCATCCCCTTATGAGCGGGCGCAGTGATTATAGGCTTTCGCTCTAAGATTAAAAATGCC

CTAGCCCACTTCTTACCACAAGGCACACCTACACCCCTTATCCCCATACTAGTTATTATCGAAACCATCA

GCCTACTCATTCAACCAATAGCCCTGGCCGTACGCCTAACCGCTAACATTACTGCAGGCCACCTACTCAT

GCACCTAATTGGAAGCGCCACCCTAGCAATATCAACCATTAACCTTCCCTCTACACTTATCATCTTCACA

ATTCTAATTCTACTAACTATCCTAGAAATCGCTGTCGCCTTAATCCAAGCCTACGTTTTCACACTTCTAG

TAAGCCTCTACCTGCACGACAACACATAATGACCCACCAATCACATGCCTATCATATAGTAAAACCCAGC

CCATGACCCCTAACAGGGGCCCTCTCAGCCCTCCTAATGACCTCCGGCCTAGCCATGTGATTTCACTTCC

ACTCCATAACGCTCCTCATACTAGGCCTACTAACCAACACACTAACCATATACCAATGATGGCGCGATGT

AACACGAGAAAGCACATACCAAGGCCACCACACACCACCTGTCCAAAAAGGCCTTCGATACGGGATAATC

CTATTTATTACCTCAGAAGTTTTTTTCTTCGCAGGATTTTTCTGAGCCTTTTACCACTCCAGCCTAGCCC

CTACCCCCCAATTAGGAGGGCACTGGCCCCCAACAGGCATCACCCCGCTAAATCCCCTAGAAGTCCCACT

CCTAAACACATCCGTATTACTCGCATCAGGAGTATCAATCACCTGAGCTCACCATAGTCTAATAGAAAAC

AACCGAAACCAAATAATTCAAGCACTGCTTATTACAATTTTACTGGGTCTCTATTTTACCCTCCTACAAG

CCTCAGAGTACTTCGAGTCTCCCTTCACCATTTCCGACGGCATCTACGGCTCAACATTTTTTGTAGCCAC

AGGCTTCCACGGACTTCACGTCATTATTGGCTCAACTTTCCTCACTATCTGCTTCATCCGCCAACTAATA

TTTCACTTTACATCCAAACATCACTTTGGCTTCGAAGCCGCCGCCTGATACTGGCATTTTGTAGATGTGG

TTTGACTATTTCTGTATGTCTCCATCTATTGATGAGGGTCTTACTCTTTTAGTATAAATAGTACCGTTAA

CTTCCAATTAACTAGTTTTGACAACATTCAAAAAAGAGTAATAAACTTCGCCTTAATTTTAATAATCAAC

ACCCTCCTAGCCTTACTACTAATAATTATTACATTTTGACTACCACAACTCAACGGCTACATAGAAAAAT

CCACCCCTTACGAGTGCGGCTTCGACCCTATATCCCCCGCCCGCGTCCCTTTCTCCATAAAATTCTTCTT

AGTAGCTATCACCTTCTTATTATTTGATCTAGAAATTGCCCTCCTTTTACCCCTACCATGAGCCCTACAA

ACAACTAACCTGCCACTAATAGTTATGTCATCCCTCTTATTAATCATCATCCTAGCCCTAAGTCTGGCCT

ATGAGTGACTACAAAAAGGATTAGACTGAACCGAATTGGTATATAGTTTAAACAAAACGAATGATTTCGA

CTCATTAAATTATGATAATCATATTTACCAAATGCCCCTCATTTACATAAATATTATACTAGCATTTACC

ATCTCACTTCTAGGAATACTAGTATATCGCTCACACCTCATATCCTCCCTACTATGCCTAGAAGGAATAA

TACTATCGCTGTTCATTATAGCTACTCTCATAACCCTCAACACCCACTCCCTCTTAGCCAATATTGTGCC

TATTGCCATACTAGTCTTTGCCGCCTGCGAAGCAGCGGTGGGCCTAGCCCTACTAGTCTCAATCTCCAAC

ACATATGGCCTAGACTACGTACATAACCTAAACCTACTCCAATGCTAAAACTAATCGTCCCAACAATTAT

ATTACTACCACTGACATGACTTTCCAAAAAACACATAATTTGAATCAACACAACCACCCACAGCCTAATT

ATTAGCATCATCCCTCTACTATTTTTTAACCAAATCAACAACAACCTATTTAGCTGTTCCCCAACCTTTT

CCTCCGACCCCCTAACAACCCCCCTCCTAATACTAACTACCTGACTCCTACCCCTCACAATCATGGCAAG

CCAACGCCACTTATCCAGTGAACCACTATCACGAAAAAAACTCTACCTCTCTATACTAATCTCCCTACAA

ATCTCCTTAATTATAACATTCACAGCCACAGAACTAATCATATTTTATATCTTCTTCGAAACCACACTTA

TCCCCACCTTGGCTATCATCACCCGATGAGGCAACCAGCCAGAACGCCTGAACGCAGGCACATACTTCCT

ATTCTACACCCTAGTAGGCTCCCTTCCCCTACTCATCGCACTAATTTACACTCACAACACCCTAGGCTCA

CTAAACATTCTACTACTCACTCTCACTGCCCAAGAACTATCAAACTCCTGAGCCAACAACTTAATATGAC

TAGCTTACACAATAGCTTTTATAGTAAAGATACCTCTTTACGGACTCCACTTATGACTCCCTAAAGCCCA

TGTCGAAGCCCCCATCGCTGGGTCAATAGTACTTGCCGCAGTACTCTTAAAACTAGGCGGCTATGGTATA

ATACGCCTCACACTCATTCTCAACCCCCTGACAAAACACATAGCCTACCCCTTCCTTGTACTATCCCTAT

GAGGCATAATTATAACAAGCTCCATCTGCCTACGACAAACAGACCTAAAATCGCTCATTGCATACTCTTC

AATCAGCCACATAGCCCTCGTAGTAACAGCCATTCTCATCCAAACCCCCTGAAGCTTCACCGGCGCAGTC

ATTCTCATAATCGCCCACGGACTTACATCCTCATTACTATTCTGCCTAGCAAACTCAAACTACGAACGCA

CTCACAGTCGCATCATAATCCTCTCTCAAGGACTTCAAACTCTACTCCCACTAATAGCTTTTTGATGACT

TCTAGCAAGCCTCGCTAACCTCGCCTTACCCCCCACTATTAACCTACTGGGAGAACTCTCTGTGCTAGTA

ACCACGTTCTCCTGATCAAATATCACTCTCCTACTTACAGGACTCAACATACTAGTCACAGCCCTATACT

CCCTCTACATATTTACCACAACACAATGGGGCTCACTCACCCACCACATTAACAACATAAAACCCTCATT

CACACGAGAAAACACCCTCATGTTCATACACCTATCCCCCATTCTCCTCCTATCCCTCAACCCCGACATC

ATTACCGGGTTTTCCTCTTGTAAATATAGTTTAACCAAAACATCAGATTGTGAATCTGACAACAGAGGCT

TACGACCCCTTATTTACCGAGAAAGCTCACAAGAACTGCTAACTCATGCCTCCATGTCTAACAACATGGC

TTTCTCAACTTTTAAAGGATAACAGCTATCCATTGGTCTTAGGCCCCAAAAATTTTGGTGCAACTCCAAA

TAAAAGTAATAACCATGCACACTACTATAACCACCCTAACCCTGACTTCCCTAATTCCCCCCATCCTTAC

CACCCTCGTTAACCCTAACAAAAAAAACTCATACCCCCATTATGTAAAATCCATTGTCGCATCCACCTTT

ATTATCAGTCTCTTCCCCACAACAATATTCATGTGCCTAGACCAAGAAGTTATTATCTCGAACTGACACT

GAGCCACAACCCAAACAACCCAGCTCTCCCTAAGCTTCAAACTAGACTACTTCTCCATAATATTCATCCC

TGTAGCATTGTTCGTTACATGGTCCATCATAGAATTCTCACTGTGATATATAAACTCAGACCCAAACATT

AATCAGTTCTTCAAATATCTACTCATCTTCCTAATTACCATACTAATCTTAGTTACCGCTAACAACCTAT

TCCAACTGTTCATCGGCTGAGAGGGCGTAGGAATTATATCCTTCTTGCTCATCAGTTGATGATACGCCCG

AGCAGATGCCAACACAGCAGCCATTCAAGCAATCCTATACAACCGTATCGGCGATATCGGTTTCATCCTC

GCCTTAGCATGATTTATCCTACACTCCAACTCATGAGACCCACAACAAATAGCCCTTCTAAACGCTAATC

CAAGCCTCACCCCACTACTAGGCCTCCTCCTAGCAGCAGCAGGCAAATCAGCCCAATTAGGTCTCCACCC

CTGACTCCCCTCAGCCATAGAAGGCCCCACCCCAGTCTCAGCCCTACTCCACTCAAGCACTATAGTTGTA

GCAGGAATCTTCTTACTCATCCGCTTCCACCCCCTAGCAGAAAATAGCCCACTAATCCAAACTCTAACAC

TATGCTTAGGCGCTATCACCACTCTGTTCGCAGCAGTCTGCGCCCTTACACAAAATGACATCAAAAAAAT

CGTAGCCTTCTCCACTTCAAGTCAACTAGGACTCATAATAGTTACAATCGGCATCAACCAACCACACCTA

GCATTCCTGCACATCTGTACCCACGCCTTCTTCAAAGCCATACTATTTATGTGCTCCGGGTCCATCATCC

ACAACCTTAACAATGAACAAGATATTCGAAAAATAGGAGGACTACTCAAAACCATACCTCTCACTTCAAC

CTCCCTCACCATTGGCAGCCTAGCATTAGCAGGAATACCTTTCCTCACAGGTTTCTACTCCAAAGACCAC

ATCATCGAAACCGCAAACATATCATACACAAACGCCTGAGCCCTATCTATTACTCTCATCGCTACCTCCC

TGACAAGCGCCTATAGCACTCGAATAATTCTTCTCACCCTAACAGGTCAACCTCGCTTCCCCACCCTTAC

TAACATTAACGAAAATAACCCCACCCTACTAAACCCCATTAAACGCCTGGCAGCCGGAAGCCTATTCGCA

GGATTTCTCATTACTAACAACATTTCCCCCGCATCCCCCTTCCAAACAACAATCCCCCTCTACCTAAAAC

TCACAGCCCTCGCTGTCACTTTCCTAGGACTTCTAACAGCCCTAGACCTCAACTACCTAACCAACAAACT

TAAAATAAAATCCCCACTATGCACATTTTATTTCTCCAACATACTCGGATTCTACCCTAGCATCACACAC

CGCACAATCCCCTATCTAGGCCTTCTTACGAGCCAAAACCTGCCCCTACTCCTCCTAGACCTAACCTGAC

TAGAAAAGCTATTGCCTAAAACAATTTCACAGCACCAAATCTCCACCTCCATCATCACCTCAACCCAAAA

AGGCATAATTAAACTTTACTTCCTCTCTTTCTTCTTCCCACTCATCCTAACCCTACTCCTAATCACATAA

CCTATTCCCCCGAGCAATCTCAATTACAATATATACACCAACAAACAATGTTCAACCAGTAACTACTACT

AATCAACGCCCATAATCATACAAAGCCCCCGCACCAATAGGATCCTCCCGAATCAACCCTGACCCCTCTC

CTTCATAAATTATTCAGCTTCCTACACTATTAAAGTTTACCACAACCACCACCCCATCATACTCTTTCAC

CCACAGCACCAATCCTACCTCCATCGCTAACCCCACTAAAACACTCACCAAGACCTCAACCCCTGACCCC

CATGCCTCAGGATACTCCTCAATAGCCATCGCTGTAGTATATCCAAAGACAACCATCATTCCCCCTAAAT

AAATTAAAAAAACTATTAAACCCATATAACCTCCCCCAAAATTCAGAATAATAACACACCCGACCACACC

GCTAACAATCAATACTAAACCCCCATAAATAGGAGAAGGCTTAGAAGAAAACCCCACAAACCCCATTACT

AAACCCACACTCAACAGAAACAAAGCATACATCATTATTCTCGCACGGACTACAACCACGACCAATGATA

TGAAAAACCATCGTTGTATTTCAACTACAAGAACACCAATGACCCCAATACGCAAAATTAACCCCCTAAT

AAAATTAATTAACCACTCATTCATCGACCTCCCCACCCCATCCAACATCTCCGCATGATGAAACTTCGGC

TCACTCCTTGGCGCCTGCCTGATCCTCCAAATCACCACAGGACTATTCCTAGCCATGCACTACTCACCAG

ACGCCTCAACCGCCTTTTCATCAATCGCCCACATCACTCGAGACGTAAATTATGGCTGAATCATCCGCTA

CCTTCACGCCAATGGCGCCTCAATATTCTTTATCTGCCTCTTCCTACACATCGGGCGAGGCCTATATTAC

GGATCATTTCTCTACTCAGAAACCTGAAACATCGGCATTATCCTCCTGCTTGCAACTATAGCAACAGCCT

TCATAGGCTATGTCCTCCCGTGAGGCCAAATATCATTCTGAGGGGCCACAGTAATTACAAACTTACTATC

CGCCATCCCATACATTGGGACAGACCTAGTTCAATGAATCTGAGGAGGCTACTCAGTAGACAGTCCCACC

CTCACACGATTCTTTACCTTTCACTTCATCTTGCCCTTCATTATTGCAGCCCTAGCAGCACTCCACCTCC

TATTCTTGCACGAAACGGGATCAAACAACCCCCTAGGAATCACCTCCCATTCCGATAAAATCACCTTCCA

CCCTTACTACACAATCAAAGACGCCCTCGGCTTACTTCTCTTCCTTCTCTCCTTAATGACATTAACACTA

TTCTCACCAGACCTCCTAGGCGACCCAGACAATTATACCCTAGCCAACCCCTTAAACACCCCTCCCCACA

TCAAGCCCGAATGATATTTCCTATTCGCCTACACAATTCTCCGATCCGTCCCTAACAAACTAGGAGGCGT

CCTTGCCCTATTACTATCCATCCTCATCCTAGCAATAATCCCCATCCTCCATATATCCAAACAACAAAGC

ATAATATTTCGCCCACTAAGCCAATCACTTTATTGACTCCTAGCCGCAGACCTCCTCGTTCTAACCTGAA

TCGGAGGACAACCAGTAAGCTACCCTTTTACCATCATTGGACAAGTAGCATCCGTACTATACTTCACAAC

AATCCTAATCCTAATACCAACTATCTCCCTAATTGAAAACAAAATACTCAAATGGGCCTGTCCTTGTAGT

ATAAACTAATACACCAGTCTTGTAAACCGGAGATGAAAACCTTTTTCCAAGGACAAATCAGAGAAAAAGT

CTTTAACTCCACCATTAGCACCCAAAGCTAAGATTCTAATTTAAACTATTCTCTGTTCTTTCATGGGGAA

GCAGATTTGGGTACCACCCAAGTATTGACTCACCCATCAACAACCGCTATGTATTTCGTACATTACTGCC

AGCCACCATGAATATTGTACGGTACCATAAATACTTGACCACCTGTAGTACATAAAAACCCAATCCACAT

CAACCCCCCCCCCCCATGCTTACAAGCAAGTACAGCAACCAACCCTCAACTATCACACATCAACTGCAGC

TCCAAAGCCACCTCTCACCCACCAGGATACCAACAAACCTACCCACCCTTAACAGTACATAGTACATAAA

GCCATTTACCGTACATAGCACATTACAGTCAAATCCCTTCTCGTCCCCATGGATGACCCCCCTCAGATAG

GGGTCCCTTGACCACCATCCTCCGTGAAATCAATATCCCGCACAAGAGTGCTACTCTCCTCGCTCCGGGC

CCATAACACTTGGGGGTAGCTAAAGTGAACTGTATCCGACATCTGGTTCCTACTTCAGGGCCATAAAGCC

TAAATAGCCCACACGTTCCCCTTAAATAAGACATCACGATG

>MT135511.1 Homo sapiens isolate NH20 mitochondrion, complete genome

GATCACAGGTCTATCACCCTATTAACCACTCACGGGAGCTCTCCATGCATTTGGTATTTTCGTCTGGGGG

GTGTGCACGCGATAGCATTGCGAGACGCTGGAGCCGGAGCACCCTATGTCGCAGTATCTGTCTTTGATTC

CTGCCCCATCTTATTATTTATCGCACCTACGTTCAATATTACAGGCGAACATACTTACTAAAGTGTGTTA

ATTAATTAATGCTTGTAGGACATAATAATAACAATTGAATGTCTGCACAGCCGCTTTCCACACAGACATC

ATAACAAAAAATTTCCACCAAACCCCCCCCCTCCCCCCGCTTCTGGCCACAGCACTTAAACACATCTCTG

CCAAACCCCAAAAACAAAGAACCCTAACACCAGCCTAACCAGATTTCAAATTTTATCTTTTGGCGGTATG

CACTTTTAACAGTCACCCCCCAACTAACACATTATTTTCCCCTCCCACTCCCATACTACTAATCTCATCA

ATACAACCCCCGCCCATCCTACCCAGCACACACACCGCTGCTAACCCCATACCCCGAACCAACCAAACCC

CAAAGACACCCCCCACAGTTTATGTAGCTTACCTCCTCAAAGCAATACACTGAAAATGTTTAGACGGGCT

CACATCACCCCATAAACAAATAGGTTTGGTCCTAGCCTTTCTATTAGCTCTTAGTAAGATTACACATGCA

AGCATCCCCGTTCCAGTGAGTTCACCCTCTAAATCACCACGATCAAAAGGGACAAGCATCAAGCACGCAG

CAATGCAGCTCAAAACGCTTAGCCTAGCCACACCCCCACGGGAAACAGCAGTGATTAACCTTTAGCAATA

AACGAAAGTTTAACTAAGCTATACTAACCCCAGGGTTGGTCAATTTCGTGCCAGCCACCGCGGTCACACG

ATTAACCCAAGTCAATAGAAGCCGGCGTAAAGAGTGTTTTAGATCACCCCCTCCCCAATAAAGCTAAAAC

TCACCTGAGTTGTAAAAAACTCCAGTTGACACAAAATAGACTACGAAAGTGGCTTTAACATATCTGAACA

CACAATAGCTAAGACCCAAACTGGGATTAGATACCCCACTATGCTTAGCCCTAAACCTCAACAGTTAAAT

CAACAAAACTGCTCGCCAGAACACTACGAGCCACAGCTTAAAACTCAAAGGACCTGGCGGTGCTTCATAT

CCCTCTAGAGGAGCCTGTTCTGTAATCGATAAACCCCGATCAACCTCACCACCTCTTGCTCAGCCTATAT

ACCGCCATCTTCAGCAAACCCTGATGAAGGCTACAAAGTAAGCGCAAGTACCCACGTAAAGACGTTAGGT

CAAGGTGTAGCCCATGAGGTGGCAAGAAATGGGCTACATTTTCTACCCCAGAAAACTACGATAGCCCTTA

TGAAACTTAAGGGTCGAAGGTGGATTTAGCAGTAAACTGAGAGTAGAGTGCTTAGTTGAACAGGGCCCTG

AAGCGCGTACACACCGCCCGTCACCCTCCTCAAGTATACTTCAAAGGACATTTAACTAAAACCCCTACGC

ATTTATATAGAGGAGACAAGTCGTAACATGGTAAGTGTACTGGAAAGTGCACTTGGACGAACCAGAGTGT

AGCTTAACACAAAGCACCCAACTTACACTTAGGAGATTTCAACTTAACTTGACCGCTCTGAGCTAAACCT

AGCCCCAAACCCGCTCCACCTTACTACCAGACAACCTTAGCCAAACCATTTACCCAAATAAAGTATAGGC

GATAGAAATTGAAACCTGGCGCAATAGATATAGTACCGCAAGGGAAAGATGAAAAATTATAACCAAGCAT

AATATAGCAAGGACTAACCCCTATACCTTCTGCATAATGAATTAACTAGAAATAACTTTGCAAGGAGAGC

CAAAGCTAAGACCCCCGAAACCAGACGAGCTACCTAAGAACAGCTAAAAGAGCACACCCGTCTATGTAGC

AAAATAGTGGGAAGATTTATAGGTAGAGGCGACAAACCTACCGAGCCTGGTGATAGCTGGTTGTCCAAGA

TAGAATCTTAGTTCAACTTTAAATTTGCCCACAGAACCCTCTAAATCCCCTTGTAAATTTAACTGTTAGT

CCAAAGAGGAACAGCTCTTTGGACACTAGGAAAAAACCTTGTAGAGAGAGTAAAAAATTTAACACCCATA

GTAGGCCTAAAAGCAGCCACCAATTAAGAAAGCGTTCAAGCTCAACACCCACTACCTAAAAAATCCCAAA

CATATAACTGAACTCCTCACACCCAATTGGACCAATCTATCACCCTATAGAAGAACTAATGTTAGTATAA

GTAACATGAAAACATTCTCCTCCGCATAAGCCTGCGTCAGATTAAAACACTGAACTGACAATTAACAGCC

CAATATCTACAATCAACCAACAAGTCATTATTACCCCCACTGTCAACCCAACACAGGCATGCTCATAAGG

AAAGGTTAAAAAAAGTAAAAGGAACTCGGCAAATCTTACCCCGCCTGTTTACCAAAAACATCACCTCTAG

CATCACCAGTATTAGAGGCACCGCCTGCCCAGTGACACATGTTTAACGGCCGCGGTACCCTAACCGTGCA

AAGGTAGCATAATCACTTGTTCCTTAAATAGGGACCTGTATGAATGGCTCCACGAGGGTTCAGCTGTCTC

TTACTTTTAACCAGTGAAATTGACCTGCCCGTGAAGAGGCGGGCATGACACAGCAAGACGAGAAGACCCT

ATGGAGCTTTAATTTATTAATGCAAACAGTACCTAACAAACCCACAGGTCCTAAACTACCAAACCTGCAT

TAAAAATTTCGGTTGGGGCGACCTCGGAGCAGAACCCAACCTCCGAGCAGTACATGCTAAGACTTCACCA

GTCAAAGCGAACTACTATACTCAATTGATCCAATAACTTGACCAACGGAACAAGTTACCCTAGGGATAAC

AGCGCAATCCTATTCTAGAGTCCATATCAACAATAGGGTTTACGACCTCGATGTTGGATCAGGACATCCC

GATGGTGCAGCCGCTATTAAAGGTTCGTTTGTTCAACGATTAAAGTCCTACGTGATCTGAGTTCAGACCG

GAGTAATCCAGGTCGGTTTCTATCTACNTTCAAATTCCTCCCTGTACGAAAGGACAAGAGAAATAAGGCC

TACTTCACAAAGCGCCTTCCCCCGTAAATGATATCATCTCAACTTAGTATTATACCCACACCCACCCAAG

AACAGGGTTTGTTAAGATGGCAGAGCCCGGTAATCGCATAAAACTTAAAACTTTACAGTCAGAGGTTCAA

TTCCTCTTCTTAACAACATACCCATGGCCAACCTCCTACTCCTCATTGTACCCATTCTAATCGCAATGGC

ATTCCTAATGCTTACCGAACGAAAAATTCTAGGCTATATACAACTACGCAAAGGCCCCAACGTTGTAGGC

CCCTACGGGCTACTACAACCCTTCGCTGACGCCATAAAACTCTTCACCAAAGAGCCCCTAAAACCCGCCA

CATCTACCATCACCCTCTACATCACCGCCCCGACCTTAGCTCTCACCATCGCTCTTCTACTATGAACCCC

CCTCCCCATACCCAACCCCCTGGTCAACCTCAACCTAGGCCTCCTATTTATTCTAGCCACCTCTAGCCTA

GCCGTTTACTCAATCCTCTGATCAGGGTGAGCATCAAACTCAAACTACGCCCTGATCGGCGCACTGCGAG

CAGTAGCCCAAACAATCTCATATGAAGTCACCCTAGCCATCATTCTACTATCAACATTACTAATAAGTGG

CTCCTTTAACCTCTCCACCCTTATCACAACACAAGAACACCTCTGATTACTCCTGCCATCATGACCCTTG

GCCATAATATGATTTATCTCCACACTAGCAGAGACCAACCGAACCCCCTTCGACCTTGCCGAAGGGGAGT

CCGAACTAGTCTCAGGCTTCAACATCGAATACGCCGCAGGCCCCTTCGCCCTATTCTTCATAGCCGAATA

CACAAACATTATTATAATAAACACCCTCACCACTACAATCTTCCTAGGAACAACATATGACGCACTCTCC

CCTGAACTCTACACAACATATTTTGTCACCAAGACCCTACTTCTAACCTCCCTGTTCTTATGAATTCGAA

CAGCATACCCCCGATTCCGCTACGACCAACTCATACACCTCCTATGAAAAAACTTCCTACCACTCACCCT

AGCATTACTTATATGATATGTCTCCATACCCATTACAATCTCCAGCATTCCCCCTCAAACCTAAGAAATA

TGTCTGATAAAAGAGTTACTTTGATAGAGTAAATAATAGGAGCTTAAACCCCCTTATTTCTAGGACTATG

AGAATCGAACCCATCCCTGAGAATCCAAAATTCTCCGTGCCACCTATCACACCCCATCCTAAAGTAAGGT

CAGCTAAATAAGCTATCGGGCCCATACCCCGAAAATGTTGGTTATACCCTTCCCGTACTAATTAATCCCC

TGGCCCAACCCGTCATCTACTCTACCATCTTTGCAGGCACACTCATCACAGCGCTAAGCTCGCACTGATT

TTTTACCTGAGTAGGCCTAGAAATAAACATGCTAGCTTTTATTCCAGTTCTAACCAAAAAAATAAACCCT

CGTTCCACAGAAGCTGCCATCAAGTATTTCCTCACGCAAGCAACCGCATCCATAATCCTTCTAATAGCTA

TCCTCTTCAACAATATACTCTCCGGACAATGAACCATAACCAATACTACCAATCAATACTCATCATTAAT

AATCATAATGGCTATAGCAATAAAACTAGGAATAGCCCCCTTTCACTTCTGAGTCCCAGAGGTTACCCAA

GGCACCCCTCTGACATCCGGCCTGCTTCTTCTCACATGACAAAAACTAGCCCCCATCTCAATCATATACC

AAATCTCTCCCTCACTAAACGTAAGCCTTCTCCTCACTCTCTCAATCTTATCCATCATAGCAGGCAGTTG

AGGTGGATTAAACCAAACCCAGCTACGCAAAATCTTAGCATACTCCTCAATTACCCACATAGGATGAATA

ATAGCAGTTCTACCGTACAACCCTAACATAACCATTCTTAATTTAACTATTTATATTATCCTAACTACTA

CCGCATTCCTACTACTCAACTTAAACTCCAGCACCACGACCCTACTACTATCTCGCACCTGAAACAAGCT

AACATGACTAACACCCTTAATTCCATCCACCCTCCTCTCCCTAGGAGGCCTGCCCCCGCTAACCGGCTTT

TTGCCCAAATGGGCCATTATCGAAGAATTCACAAAAAACAATAGCCTCATCATCCCCACCATCATAGCCA

CCATCACCCTCCTTAACCTCTACTTCTACCTACGCCTAATCTACTCCACCTCAATCACACTACTCCCCAT

ATCTAACAACGTAAAAATAAAATGACAGTTTGAACATACAAAACCCACCCCATTCCTCCCCACACTCATC

GCCCTCACCACGCTACTCCTACCTATCTCCCCTTTTATACTAATAATCTTATAGAAATTTAGGTTAAATA

CAGACCAAGAGCCTTCAAAGCCCTCAGTAAGTTGCAATACTTAATTTCTGTAACAGCTAAGGACTGCAAA

ACCCCACTCTGCATCAACTGAACGCAAATCAGCCACTTTAATTAAGCTAAGCCCTTACTAGACCAATGGG

ACTTAAACCCACAAACACTTAGTTAACAGCTAAGCACCCTAATCAACTGGCTTCAATCTACTTCTCCCGC

CGCCGGGAAAAAAGGCGGGAGAAGCCCCGGCAGGTTTGAAGCTGCTTCTTCGAATTTGCAATTCAATATG

AAAATCACCTCGGAGCTGGTAAAAAGAGGCCTAACCCCTGTCTTTAGATTTACAGTCCAATGCTTCACTC

AGCCATTTTACCTCACCCCCACTGATGTTCGCCGACCGTTGACTATTCTCTACAAACCACAAAGACATTG

GAACACTATACCTATTATTCGGCGCATGAGCTGGAGTCCTAGGCACAGCTCTAAGCCTCCTTATTCGAGC

CGAGCTGGGCCAGCCAGGCAACCTTCTAGGTAACGACCACATCTACAACGTTATCGTCACAGCCCATGCA

TTTGTAATAATCTTCTTCATAGTAATACCCATCATAATCGGAGGCTTTGGCAACTGACTAGTTCCCCTAA

TAATCGGTGCCCCCGATATGGCGTTTCCCCGCATAAACAACATAAGCTTCTGACTCTTACCTCCCTCTCT

CCTACTCCTGCTCGCATCTGCTATAGTGGAGGCCGGAGCAGGAACAGGTTGAACAGTCTACCCTCCCTTA

GCAGGGAACTACTCCCACCCTGGAGCCTCCGTAGACCTAACCATCTTCTCCTTACACCTAGCAGGTGTCT

CCTCTATCTTAGGGGCCATCAATTTCATCACAACAATTATCAATATAAAACCCCCTGCCATAACCCAATA

CCAAACGCCCCTCTTCGTCTGATCCGTCCTAATCACAGCAGTCCTACTTCTCCTATCTCTCCCAGTCCTA

GCTGCTGGCATCACTATACTACTAACAGACCGCAACCTCAACACCACCTTCTTCGACCCCGCCGGAGGAG

GAGACCCCATTCTATACCAACACCTATTCTGATTTTTCGGTCACCCTGAAGTTTATATTCTTATCCTACC

AGGCTTCGGAATAATCTCCCATATTGTAACTTACTACTCCGGAAAAAAAGAACCATTTGGATACATAGGC

ATGGTCTGAGCTATGATATCAATTGGCTTCCTAGGGTTTATCGTGTGAGCACACCATATATTTACAGTAG

GAATAGACGTAGACACACGAGCATATTTCACCTCCGCTACCATAATCATCGCTATCCCCACCGGCGTCAA

AGTATTTAGCTGACTCGCCACACTCCACGGAAGCAATATGAAATGATCTGCTGCAGTGCTCTGAGCCCTA

GGATTCATCTTTCTTTTCACCGTAGGTGGCCTGACTGGCATTGTATTAGCAAACTCATCACTAGACATCG

TACTACACGACACGTACTACGTTGTAGCTCACTTCCACTATGTCCTATCAATAGGAGCTGTATTTGCCAT

CATAGGAGGCTTCATTCACTGATTTCCCCTATTCTCAGGCTACACCCTAGACCAAACCTACGCCAAAATC

CATTTCACTATCATATTCATCGGCGTAAATCTAACTTTCTTCCCACAACACTTTCTCGGCCTATCCGGAA

TGCCCCGACGTTACTCGGACTACCCCGATGCATACACCACATGAAACATCCTATCATCTGTAGGCTCATT

CATTTCTCTAACAGCAGTAATATTAATAATTTTCATGATTTGAGAAGCCTTCGCTTCGAAGCGAAAAGTC

CTAATAGTAGAAGAACCCTCCATAAACCTGGAGTGACTATATGGATGCCCCCCACCCTACCACACATTCG

AAGAACCCGTATACATAAAATCTAGACAAAAAAGGAAGGAATCGAACCCCCCAAAGCTGGTTTCAAGCCA

ACCCCATGGCCTCCATGACTTTTTCAAAAAGGTATTAGAAAAACCATTTCATAACTTTGTCAAAGTTAAA

TTATAGGCTAAATCCTATATATCTTAATGGCACATGCAGCGCAAGTAGGTCTACAAGACGCTACTTCCCC

TATCATAGAAGAGCTTATCACCTTTCATGATCACGCCCTCATAATCATTTTCCTTATCTGCTTCCTAGTC

CTGTATGCCCTTTTCCTAACACTCACAACAAAACTAACTAATACTAACATCTCAGACGCTCAGGAAATAG

AAACCGTCTGAACTATCCTGCCCGCCATCATCCTAGTCCTCATCGCCCTCCCATCCCTACGCATCCTTTA

CATAACAGACGAGGTCAACGATCCCTCCCTTACCATCAAATCAATTGGCCACCAATGGTACTGAACCTAC

GAGTACACCGACTACGGCGGACTAATCTTCAACTCCTACATACTTCCCCCATTATTCCTAGAACCAGGCG

ACCTGCGACTCCTTGACGTTGACAATCGAGTAGTACTCCCGATTGAAGCCCCCATTCGTATAATAATTAC

ATCACAAGACGTCTTGCACTCATGAGCTGTCCCCACATTAGGCTTAAAAACAGATGCAATTCCCGGACGT

CTAAACCAAACCACTTTCACCGCTACACGACCGGGGGTATACTACGGTCAATGCTCTGAAATCTGTGGAG

CAAACCACAGTTTCATGCCCATCGTCCTAGAATTAATTCCCCTAAAAATCTTTGAAATAGGGCCCGTATT

TACCCTATAGCACCCCCTCTAGAGCCCACTGTAAAGCTAACTTAGCATTAACCTTTTAAGTTAAAGATTA

AGAGAACCAACACCTCTTTACAGTGAAATGCCCCAACTAAATACTACCGTATGGCCCACCATAATTACCC

CCATACTCCTTACACTATTCCTCATCACCCAACTAAAAATATTAAACACAAACTACCACCTACCTCCCTC

ACCAAAGCCCATAAAAATAAAAAATTATAACAAACCCTGAGAACCAAAATGAACGAAAATCTGTTCGCTT

CATTCATTGCCCCCACAATCCTAGGCCTACCCGCCGCAGTACTGATCATTCTATTTCCCCCTCTATTGAT

CCCCACCTCCAAATATCTCATCAACAACCGACTAATCACCACCCAACAATGACTAATCAAACTAACCTCA

AAACAAATGATAACCATACACAACACTAAAGGACGAACCTGATCTCTTATACTAGTATCCTTAATCATTT

TTATTGCCACAACTAACCTCCTCGGACTCCTGCCTCACTCATTTACACCAACCACCCAACTATCTATAAA

CCTAGCCATGGCCATCCCCTTATGAGCGGGCGCAGTGATTATAGGCTTTCGCTCTAAGATTAAAAATGCC

CTAGCCCACTTCTTACCACAAGGCACACCTACACCCCTTATCCCCATACTAGTTATTATCGAAACCATCA

GCCTACTCATTCAACCAATAGCCCTGGCCGTACGCCTAACCGCTAACATTACTGCAGGCCACCTACTCAT

GCACCTAATTGGAAGCGCCACCCTAGCAATATCAACCATTAACCTTCCCTCTACACTTATCATCTTCACA

ATTCTAATTCTACTAACTATCCTAGAAATCGCTGTCGCCTTAATCCAAGCCTACGTTTTCACACTTCTAG

TAAGCCTCTACCTGCACGACAACACATAATGACCCACCAATCACATGCCTATCATATAGTAAAACCCAGC

CCATGACCCCTAACAGGGGCCCTCTCAGCCCTCCTAATGACCTCCGGCCTAGCCATGTGATTTCACTTCC

ACTCCATAACGCTCCTCATACTAGGCCTACTAACCAACACACTAACCATATACCAATGATGGCGCGATGT

AACACGAGAAAGCACATACCAAGGCCACCACACACCACCTGTCCAAAAAGGCCTTCGATACGGGATAATC

CTATTTATTACCTCAGAAGTTTTTTTCTTCGCAGGATTTTTCTGAGCCTTTTACCACTCCAGCCTAGCCC

CTACCCCCCAATTAGGAGGGCACTGGCCCCCAACAGGCATCACCCCGCTAAATCCCCTAGAAGTCCCACT

CCTAAACACATCCGTATTACTCGCATCAGGAGTATCAATCACCTGAGCTCACCATAGTCTAATAGAAAAC

AACCGAAACCAAATAATTCAAGCACTGCTTATTACAATTTTACTGGGTCTCTATTTTACCCTCCTACAAG

CCTCAGAGTACTTCGAGTCTCCCTTCACCATTTCCGACGGCATCTACGGCTCAACATTTTTTGTAGCCAC

AGGCTTCCACGGACTTCACGTCATTATTGGCTCAACTTTCCTCACTATCTGCTTCATCCGCCAACTAATA

TTTCACTTTACATCCAAACATCACTTTGGCTTCGAAGCCGCCGCCTGATACTGGCATTTTGTAGATGTGG

TTTGACTATTTCTGTATGTCTCCATCTATTGATGAGGGTCTTACTCTTTTAGTATAAATAGTACCGTTAA

CTTCCAATTAACTAGTTTTGACAACATTCAAAAAAGAGTAATAAACTTCGCCTTAATTTTAATAATCAAC

ACCCTCCTAGCCTTACTACTAATAATTATTACATTTTGACTACCACAACTCAACGGCTACATAGAAAAAT

CCACCCCTTACGAGTGCGGCTTCGACCCTATATCCCCCGCCCGCGTCCCTTTCTCCATAAAATTCTTCTT

AGTAGCTATCACCTTCTTATTATTTGATCTAGAAATTGCCCTCCTTTTACCCCTACCATGAGCCCTACAA

ACAACTAACCTGCCACTAATAGTTATGTCATCCCTCTTATTAATCATCATCCTAGCCCTAAGTCTGGCCT

ATGAGTGACTACAAAAAGGATTAGACTGAACCGAATTGGTATATAGTTTAAACAAAACGAATGATTTCGA

CTCATTAAATTATGATAATCATATTTACCAAATGCCCCTCATTTACATAAATATTATACTAGCATTTACC

ATCTCACTTCTAGGAATACTAGTATATCGCTCACACCTCATATCCTCCCTACTATGCCTAGAAGGAATAA

TACTATCGCTGTTCATTATAGCTACTCTCATAACCCTCAACACCCACTCCCTCTTAGCCAATATTGTGCC

TATTGCCATACTAGTCTTTGCCGCCTGCGAAGCAGCGGTGGGCCTAGCCCTACTAGTCTCAATCTCCAAC

ACATATGGCCTAGACTACGTACATAACCTAAACCTACTCCAATGCTAAAACTAATCGTCCCAACAATTAT

ATTACTACCACTGACATGACTTTCCAAAAAACACATAATTTGAATCAACACAACCACCCACAGCCTAATT

ATTAGCATCATCCCTCTACTATTTTTTAACCAAATCAACAACAACCTATTTAGCTGTTCCCCAACCTTTT

CCTCCGACCCCCTAACAACCCCCCTCCTAATACTAACTACCTGACTCCTACCCCTCACAATCATGGCAAG

CCAACGCCACTTATCCAGTGAACCACTATCACGAAAAAAACTCTACCTCTCTATACTAATCTCCCTACAA

ATCTCCTTAATTATAACATTCACAGCCACAGAACTAATCATATTTTATATCTTCTTCGAAACCACACTTA

TCCCCACCTTGGCTATCATCACCCGATGAGGCAACCAGCCAGAACGCCTGAACGCAGGCACATACTTCCT

ATTCTACACCCTAGTAGGCTCCCTTCCCCTACTCATCGCACTAATTTACACTCACAACACCCTAGGCTCA

CTAAACATTCTACTACTCACTCTCACTGCCCAAGAACTATCAAACTCCTGAGCCAACAACTTAATATGAC

TAGCTTACACAATAGCTTTTATAGTAAAGATACCTCTTTACGGACTCCACTTATGACTCCCTAAAGCCCA

TGTCGAAGCCCCCATCGCTGGGTCAATAGTACTTGCCGCAGTACTCTTAAAACTAGGCGGCTATGGTATA

ATACGCCTCACACTCATTCTCAACCCCCTGACAAAACACATAGCCTACCCCTTCCTTGTACTATCCCTAT

GAGGCATAATTATAACAAGCTCCATCTGCCTACGACAAACAGACCTAAAATCGCTCATTGCATACTCTTC

AATCAGCCACATAGCCCTCGTAGTAACAGCCATTCTCATCCAAACCCCCTGAAGCTTCACCGGCGCAGTC

ATTCTCATAATCGCCCACGGACTTACATCCTCATTACTATTCTGCCTAGCAAACTCAAACTACGAACGCA

CTCACAGTCGCATCATAATCCTCTCTCAAGGACTTCAAACTCTACTCCCACTAATAGCTTTTTGATGACT

TCTAGCAAGCCTCGCTAACCTCGCCTTACCCCCCACTATTAACCTACTGGGAGAACTCTCTGTGCTAGTA

ACCACGTTCTCCTGATCAAATATCACTCTCCTACTTACAGGACTCAACATACTAGTCACAGCCCTATACT

CCCTCTACATATTTACCACAACACAATGGGGCTCACTCACCCACCACATTAACAACATAAAACCCTCATT

CACACGAGAAAACACCCTCATGTTCATACACCTATCCCCCATTCTCCTCCTATCCCTCAACCCCGACATC

ATTACCGGGTTTTCCTCTTGTAAATATAGTTTAACCAAAACATCAGATTGTGAATCTGACAACAGAGGCT

TACGACCCCTTATTTACCGAGAAAGCTCACAAGAACTGCTAACTCATGCCTCCATGTCTAACAACATGGC

TTTCTCAACTTTTAAAGGATAACAGCTATCCATTGGTCTTAGGCCCCAAAAATTTTGGTGCAACTCCAAA

TAAAAGTAATAACCATGCACACTACTATAACCACCCTAACCCTGACTTCCCTAATTCCCCCCATCCTTAC

CACCCTCGTTAACCCTAACAAAAAAAACTCATACCCCCATTATGTAAAATCCATTGTCGCATCCACCTTT

ATTATCAGTCTCTTCCCCACAACAATATTCATGTGCCTAGACCAAGAAGTTATTATCTCGAACTGACACT

GAGCCACAACCCAAACAACCCAGCTCTCCCTAAGCTTCAAACTAGACTACTTCTCCATAATATTCATCCC

TGTAGCATTGTTCGTTACATGGTCCATCATAGAATTCTCACTGTGATATATAAACTCAGACCCAAACATT

AATCAGTTCTTCAAATATCTACTCATCTTCCTAATTACCATACTAATCTTAGTTACCGCTAACAACCTAT

TCCAACTGTTCATCGGCTGAGAGGGCGTAGGAATTATATCCTTCTTGCTCATCAGTTGATGATACGCCCG

AGCAGATGCTAACACAGCAGCCATTCAAGCAATCCTATACAACCGTATCGGCGATATCGGTTTCATCCTC

GCCTTAGCATGATTTATCCTACACTCCAACTCATGAGACCCACAACAAATAGCCCTTCTAAACGCTAATC

CAAGCCTCACCCCACTACTAGGCCTCCTCCTAGCAGCAGCAGGCAAATCAGCCCAATTAGGTCTCCACCC

CTGACTCCCCTCAGCCATAGAAGGCCCCACCCCAGTCTCAGCCCTACTCCACTCAAGCACTATAGTTGTA

GCAGGAATCTTCTTACTCATCCGCTTCCACCCCCTAGCAGAAAATAGCCCACTAATCCAAACTCTAACAC

TATGCTTAGGCGCTATCACCACTCTGTTCGCAGCAGTCTGCGCCCTTACACAAAATGACATCAAAAAAAT

CGTAGCCTTCTCCACTTCAAGTCAACTAGGACTCATAATAGTTACAATCGGCATCAACCAACCACACCTA

GCATTCCTGCACATCTGTACCCACGCCTTCTTCAAAGCCATACTATTTATGTGCTCCGGGTCCATCATCC

ACAACCTTAACAATGAACAAGATATTCGAAAAATAGGAGGACTACTCAAAACCATACCTCTCACTTCAAC

CTCCCTCACCATTGGCAGCCTAGCATTAGCAGGAATACCTTTCCTCACAGGTTTCTACTCCAAAGACCAC

ATCATCGAAACCGCAAACATATCATACACAAACGCCTGAGCCCTATCTATTACTCTCATCGCTACCTCCC

TGACAAGCGCCTATAGCACTCGAATAATTCTTCTCACCCTAACAGGTCAACCTCGCTTCCCCACCCTTAC

TAACATTAACGAAAATAACCCCACCCTACTAAACCCCATTAAACGCCTGGCAGCCGGAAGCCTATTCGCA

GGATTTCTCATTACTAACAACATTTCCCCCGCATCCCCCTTCCAAACAACAATCCCCCTCTACCTAAAAC

TCACAGCCCTCGCTGTCACTTTCCTAGGACTTCTAACAGCCCTAGACCTCAACTACCTAACCAACAAACT

TAAAATAAAATCCCCACTATGCACATTTTATTTCTCCAACATACTCGGATTCTACCCTAGCATCACACAC

CGCACAATCCCCTATCTAGGCCTTCTTACGAGCCAAAACCTGCCCCTACTCCTCCTAGACCTAACCTGAC

TAGAAAAGCTATTGCCTAAAACAATTTCACAGCACCAAATCTCCACCTCCATCATCACCTCAACCCAAAA

AGGCATAATTAAACTTTACTTCCTCTCTTTCTTCTTCCCACTCATCCTAACCCTACTCCTAATCACATAA

CCTATTCCCCCGAGCAATCTCAATTACAATATATACACCAACAAACAATGTTCAACCAGTAACTACTACT

AATCAACGCCCATAATCATACAAAGCCCCCGCACCAATAGGATCCTCCCGAATCAACCCTGACCCCTCTC

CTTCATAAATTATTCAGCTTCCTACACTATTAAAGTTTACCACAACCACCACCCCATCATACTCTTTCAC

CCACAGCACCAATCCTACCTCCATCGCTAACCCCACTAAAACACTCACCAAGACCTCAACCCCTGACCCC

CATGCCTCAGGATACTCCTCAATAGCCATCGCTGTAGTATATCCAAAGACAACCATCATTCCCCCTAAAT

AAATTAAAAAAACTATTAAACCCATATAACCTCCCCCAAAATTCAGAATAATAACACACCCGACCACACC

GCTAACAATCAATACTAAACCCCCATAAATAGGAGAAGGCTTAGAAGAAAACCCCACAAACCCCATTACT

AAACCCACACTCAACAGAAACAAAGCATACATCATTATTCTCGCACGGACTACAACCACGACCAATGATA

TGAAAAACCATCGTTGTATTTCAACTACAAGAACACCAATGACCCCAATACGCAAAATTAACCCCCTAAT

AAAATTAATTAACCACTCATTCATCGACCTCCCCACCCCATCCAACATCTCCGCATGATGAAACTTCGGC

TCACTCCTTGGCGCCTGCCTGATCCTCCAAATCACCACAGGACTATTCCTAGCCATGCACTACTCACCAG

ACGCCTCAACCGCCTTTTCATCAATCGCCCACATCACTCGAGACGTAAATTATGGCTGAATCATCCGCTA

CCTTCACGCCAATGGCGCCTCAATATTCTTTATCTGCCTCTTCCTACACATCGGGCGAGGCCTATATTAC

GGATCATTTCTCTACTCAGAAACCTGAAACATCGGCATTATCCTCCTGCTTGCAACTATAGCAACAGCCT

TCATAGGCTATGTCCTCCCGTGAGGCCAAATATCATTCTGAGGGGCCACAGTAATTACAAACTTACTATC

CGCCATCCCATACATTGGGACAGACCTAGTTCAATGAATCTGAGGAGGCTACTCAGTAGACAGTCCCACC

CTCACACGATTCTTTACCTTTCACTTCATCTTGCCCTTCATTATTGCAGCCCTAGCAGCACTCCACCTCC

TATTCTTGCACGAAACGGGATCAAACAACCCCCTAGGAATCACCTCCCATTCCGATAAAATCACCTTCCA

CCCTTACTACACAATCAAAGACGCCCTCGGCTTACTTCTCTTCCTTCTCTCCTTAATGACATTAACACTA

TTCTCACCAGACCTCCTAGGCGACCCAGACAATTATACCCTAGCCAACCCCTTAAACACCCCTCCCCACA

TCAAGCCCGAATGATATTTCCTATTCGCCTACACAATTCTCCGATCCGTCCCTAACAAACTAGGAGGCGT

CCTTGCCCTATTACTATCCATCCTCATCCTAGCAATAATCCCCATCCTCCATATATCCAAACAACAAAGC

ATAATATTTCGCCCACTAAGCCAATCACTTTATTGACTCCTAGCCGCAGACCTCCTCGTTCTAACCTGAA

TCGGAGGACAACCAGTAAGCTACCCTTTTACCATCATTGGACAAGTAGCATCCGTACTATACTTCACAAC

AATCCTAATCCTAATACCAACTATCTCCCTAATTGAAAACAAAATACTCAAATGGGCCTGTCCTTGTAGT

ATAAACTAATACACCAGTCTTGTAAACCGGAGATGAAAACCTTTTTCCAAGGACAAATCAGAGAAAAAGT

CTTTAACTCCACCATTAGCACCCAAAGCTAAGATTCTAATTTAAACTATTCTCTGTTCTTTCATGGGGAA

GCAGATTTGGGTACCACCCAAGTATTGACTCACCCATCAACAACCGCTATGTATTTCGTACATTACTGCC

AGCCACCATGAATATTGTACGGTACCATAAATACTTGACCACCTGTAGTACATAAAAACCCAATCCACAT

CAACCCCCCCCCCCCATGCTTACAAGCAAGTACAGCAACCAACCCTCAACTATCACACATCAACTGCAGC

TCCAAAGCCACCTCTCACCCACTAGGATACCAACAAACCTACCCACCCTTAACAGTACATAGTACATAAA

GCCATTTACCGTACATAGCACATTACAGTCAAATCCCTTCTCGTCCCCATGGATGACCCCCCTCAGATAG

GGGTCCCTTGACCACCATCCTCCGTGAAATCAATATCCCGCACAAGAGTGCTACTCTCCTCGCTCCGGGC

CCATAACACTTGGGGGTAGCTAAAGTGAACTGTATCCGACATCTGGTTCCTACTTCAGGGCCATAAAGCC

TAAATAGCCCACACGTTCCCCTTAAATAAGACATCACGATG

>MT135512.1 Homo sapiens isolate NH25 mitochondrion, complete genome

GATCACAGGTCTATCACCCTATTAACCACTCACGGGAGCTCTCCATGCATTTGGTATTTTCGTCTGGGGG

GTGTGCACGCGATAGCATTGCGAGACGCTGGAGCCGGAGCACCCTATGTCGCAGTATCTGTCTTTGATTC

CTGCCCCATCCTATTATTTATCGCACCTACGTTCAATATTACAGGCGAACATACTTACTAAAGTGTGTTA

ATTAATTAATGCTTGTAGGACATAATAATAACAATTGAATGTCTGCACAGCCGCTTTCCACACAGACATC

ATAACAAAAAATTTCCACCAAACCCCCCCCTCCCCCCGCTTCTGGCCACAGCACTTAAACACATCTCTGC

CAAACCCCAAAAACAAAGAACCCTAACACCAGCCTAACCAGATTTCAAATTTTATCTTTTGGCGGTATGC

ACTTTTAACAGTCACCCCCCAACTAACACATTATTTTCCCCTCCCACTCCCATACTACTAATCTCATCAA

TACAACCCCCGCCCATCCTACCCAGCACACACACCGCTGCTAACCCCATACCCCGAACCAACCAAACCCC

AAAGACACCCCCCACAGTTTATGTAGCTTACCTCCTCAAAGCAATACACTGAAAATGTTTAGACGGGCTC

ACATCACCCCATAAACAAATAGGTTTGGTCCTAGCCTTTCTATTAGCTCTTAGTAAGATTACACATGCAA

GCATCCCCGTTCCAGTGAGTTCACCCTCTAAATCACCACGATCAAAAGGGACAAGCATCAAGCACGCAGC

AATGCAGCTCAAAACGCTTAGCCTAGCCACACCCCCACGGGAAACAGCAGTGATTAACCTTTAGCAATAA

ACGAAAGTTTAACTAAGCTATACTAACCCCAGGGTTGGTCAATTTCGTGCCAGCCACCGCGGTCACACGA

TTAACCCAAGTCAATAGAAGCCGGCGTAAAGAGTGTTTTAGATCACCCCCTCCCCAATAAAGCTAAAACT

CACCTGAGTTGTAAAAAACTCCAGTTGACACAAAATAGACTACGAAAGTGGCTTTAACATATCTGAACAC

ACAATAGCTAAGACCCAAACTGGGATTAGATACCCCACTATGCTTAGCCCTAAACCTCAACAGTTAAATC

AACAAAACTGCTCGCCAGAACACTACGAGCCACAGCTTAAAACTCAAAGGACCTGGCGGTGCTTCATATC

CCTCTAGAGGAGCCTGTTCTGTAATCGATAAACCCCGATCAACCTCACCACCTCTTGCTCAGCCTATATA

CCGCCATCTTCAGCAAACCCTGATGAAGGCTACAAAGTAAGCGCAAGTACCCACGTAAAGACGTTAGGTC

AAGGTGTAGCCCATGAGGTGGCAAGAAATGGGCTACATTTTCTACCCCAGAAAACTACGATAGCCCTTAT

GAAACTTAAGGGTCGAAGGTGGATTTAGCAGTAAACTGAGAGTAGAGTGCTTAGTTGAACAGGGCCCTGA

AGCGCGTACACACCGCCCGTCACCCTCCTCAAGTATACTTCAAAGGACATTTAACTAAAACCCCTACGCA

TTTATATAGAGGAGACAAGTCGTAACATGGTAAGTGTACTGGAAAGTGCACTTGGACGAACCAGAGTGTA

GCTTAACACAAAGCACCCAACTTACACTTAGGAGATTTCAACTTAACTTGACCGCTCTGAGCTAAACCTA

GCCCCAAACCCACTCCACCTTACTACCAGACAACCTTAGCCAAACCATTTACCCAAATAAAGTATAGGCG

ATAGAAATTGAAACCTGGCGCAATAGATATAGTACCGCAAGGGAAAGATGAAAAATTATAACCAAGCATA

ATATAGCAAGGACTAACCCCTATACCTTCTGCATAATGAATTAACTAGAAATAACTTTGCAAGGAGAGCC

AAAGCTAAGACCCCCGAAACCAGACGAGCTACCTAAGAACAGCTAAAAGAGCACACCCGTCTATGTAGCA

AAATAGTGGGAAGATTTATAGGTAGAGGCGACAAACCTACCGAGCCTGGTGATAGCTGGTTGTCCAAGAT

AGAATCTTAGTTCAACTTTAAATTTGCCCACAGAACCCTCTAAATCCCCTTGTAAATTTAACTGTTAGTC

CAAAGAGGAACAGCTCTTTGGACACTAGGAAAAAACCTTGTAGAGAGAGTAAAAAATTTAACACCCATAG

TAGGCCTAAAAGCAGCCACCAATTAAGAAAGCGTTCAAGCTCAACACCCACTACCTAAAAAATCCCAAAC

ATATAACTGAACTCCTCACACCCAATTGGACCAATCTATCACCCTATAGAAGAACTAATGTTAGTATAAG

TAACATGAAAACATTCTCCTCCGCATAAGCCTGCGTCAGATTAAAACACTGAACTGACAATTAACAGCCC

AATATCTACAATCAACCAACAAGTCATTATTACCCTCACTGTCAACCCAACACAGGCATGCTCATAAGGA

AAGGTTAAAAAAAGTAAAAGGAACTCGGCAAATCTTACCCCGCCTGTTTACCAAAAACATCACCTCTAGC

ATCACCAGTATTAGAGGCACCGCCTGCCCAGTGACACATGTTTAACGGCCGCGGTACCCTAACCGTGCAA

AGGTAGCATAATCACTTGTTCCTTAAATAGGGACCTGTATGAATGGCTCCACGAGGGTTCAGCTGTCTCT

TACTTTTAACCAGTGAAATTGACCTGCCCGTGAAGAGGCGGGCATGACACAGCAAGACGAGAAGACCCTA

TGGAGCTTTAATTTATTAATGCAAACAGTACCTAACAAACCCACAGGTCCTAAACTACCAAACCTGCATT

AAAAATTTCGGTTGGGGCGACCTCGGAGCAGAACCCAACCTCCGAGCAGTACATGCTAAGACTTCACCAG

TCAAAGCGAACTACTATACTCAATTGATCCAATAACTTGACCAACGGAACAAGTTACCCTAGGGATAACA

GCGCAATCCTATTCTAGAGTCCATATCAACAATAGGGTTTACGACCTCGATGTTGGATCAGGACATCCCG

ATGGTGCAGCCGCTATTAAAGGTTCGTTTGTTCAACGATTAAAGTCCTACGTGATCTGAGTTCAGACCGG

AGTAATCCAGGTCGGTTTCTATCTACNTTCAAATTCCTCCCTGTACGAAAGGACAAGAGAAATAAGGCCT

ACTTCACAAAGCGCCTTCCCCCGTAAATGATATCATCTCAACTTAGTATTATACCCACACCCACCCAAGA

ACAGGGTTTGTTAAGATGGCAGAGCCCGGTAATCGCATAAAACTTAAAACTTTACAGTCAGAGGTTCAAT

TCCTCTTCTTAACAACATACCCATGGCCAACCTCCTACTCCTCATTGTACCCATTCTAATCGCAATGGCA

TTCCTAATGCTTACCGAACGAAAAATTCTAGGCTATATACAACTACGCAAAGGCCCCAACGTTGTAGGCC

CCTACGGGCTACTACAACCCTTCGCTGACGCCATAAAACTCTTCACCAAAGAGCCCCTAAAACCCGCCAC

ATCTACCATCACCCTCTACATCACCGCCCCGACCTTAGCTCTCACCATCGCTCTTCTACTATGAACCCCC

CTCCCCATACCCAACCCCCTGGTCAACCTCAACCTAGGCCTCCTATTTATTCTAGCCACCTCTAGCCTAG

CCGTTTACTCAATCCTCTGATCAGGGTGAGCATCAAACTCAAACTACGCCCTGATCGGCGCACTGCGAGC

AGTAGCCCAAACAATCTCATATGAAGTCACCCTAGCCATCATTCTACTATCAACATTACTAATAAGTGGC

TCCTTTAACCTCTCCACCCTTATCACAACACAAGAACACCTCTGATTACTCCTGCCATCATGACCCTTGG

CCATAATATGATTTATCTCCACACTAGCAGAGACCAACCGAACCCCCTTCGACCTTGCCGAAGGGGAGTC

CGAACTAGTCTCAGGCTTCAACATCGAATACGCCGCAGGCCCCTTCGCCCTATTCTTCATAGCCGAATAC

ACAAACATTATTATAATAAACACCCTCACCACTACAATCTTCCTAGGAACAACATATGACGCACTCTCCC

CTGAACTCTACACAACATATTTTGTCACCAAGACCCTACTTCTAACCTCCCTGTTCTTATGAATTCGAAC

AGCATACCCCCGATTCCGCTACGACCAACTCATACACCTCCTATGAAAAAACTTCCTACCACTCACCCTA

GCATTACTTATATGATATGTCTCCATACCCATTACAATCTCCAGCATTCCCCCTCAAACCTAAGAAATAT

GTCTGATAAAAGAGTTACTTTGATAGAGTAAATAATAGGAGCTTAAACCCCCTTATTTCTAGGACTATGA

GAATCGAACCCATCCCTGAGAATCCAAAATTCTCCGTGCCACCTATCACACCCCATCCTAAAGTAAGGTC

AGCTAAATAAGCTATCGGGCCCATACCCCGAAAATGTTGGTTATACCCTTCCCGTACTAATTAATCCCCT

GGCCCAACCCGTCATCTACTCTACCATCTTTGCAGGCACACTCATCACAGCGCTAAGCTCGCACTGATTT

TTTACCTGAGTAGGCCTAGAAATAAACATGCTAGCTTTTATTCCAGTTCTAACCAAAAAAATAAACCCTC

GTTCCACAGAAGCTGCCATCAAGTATTTCCTCACGCAAGCAACCGCATCCATAATCCTTCTAATAGCTAT

CCTCTTCAACAATATACTCTCCGGACAATGAACCATAACCAATACTACCAATCAATACTCATCATTAATA

ATCATAATGGCTATAGCAATAAAACTAGGAATAGCCCCCTTTCACTTCTGAGTCCCAGAGGTTACCCAAG

GCACCCCTCTGACATCCGGCCTGCTTCTTCTCACATGACAAAAACTAGCCCCCATCTCAATCATATACCA

AATCTCTCCCTCACTAAACGTAAGCCTTCTCCTCACTCTCTCAATCTTATCCATCATAGCAGGCAGTTGA

GGTGGATTAAACCAAACCCAGCTACGCAAAATCTTAGCATACTCCTCAATTACCCACATAGGATGAATAA

TAGCAGTTCTACCGTACAACCCTAACATAACCATTCTTAATTTAACTATTTATATTATCCTAACTACTAC

CGCATTCCTACTACTCAACTTAAACTCCAGCACCACGACCCTACTACTATCTCGCACCTGAAACAAGCTA

ACATGACTAACACCCTTAATTCCATCCACCCTCCTCTCCCTAGGAGGCCTGCCCCCGCTAACCGGCTTTT

TGCCCAAATGGGCCATTATCGAAGAATTCACAAAAAACAATAGCCTCATCATCCCCACCATCATAGCCAC

CATCACCCTCCTTAACCTCTACTTCTACCTACGCCTAATCTACTCCACCTCAATCACACTACTCCCCATA

TCTAACAACGTAAAAATAAAATGACAGTTTGAACATACAAAACCCACCCCATTCCTCCCCACACTCATCG

CCCTCACCACGCTACTCCTACCTATCTCCCCTTTTATACTAATAATCTTATAGAAATTTAGGTTAAATAC

AGACCAAGAGCCTTCAAAGCCCTCAGTAAGTTGCAATACTTAATTTCTGTAACAGCTAAGGACTGCAAAA

CCCCACTCTGCATCAACTGAACGCAAATCAGCCACTTTAATTAAGCTAAGCCCTTACTAGACCAATGGGA

CTTAAACCCACAAACACTTAGTTAACAGCTAAGCACCCTAATCAACTGGCTTCAATCTACTTCTCCCGCC

GCCGGGAAAAAAGGCGGGAGAAGCCCCGGCAGGTTTGAAGCTGCTTCTTCGAATTTGCAATTCAATATGA

AAATCACCTCGGAGCTGGTAAAAAGAGGCCTAACCCCTGTCTTTAGATTTACAGTCCAATGCTTCACTCA

GCCATTTTACCTCACCCCCACTGATGTTCGCCGACCGTTGACTATTCTCTACAAACCACAAAGACATTGG

AACACTATACCTATTATTCGGCGCATGAGCTGGAGTCCTAGGCACAGCTCTAAGCCTCCTTATTCGAGCC

GAGCTGGGCCAGCCAGGCAACCTTCTAGGTAACGACCACATCTACAACGTTATCGTCACAGCCCATGCAT

TTGTAATAATCTTCTTCATAGTAATACCCATCATAATCGGAGGCTTTGGCAACTGACTAGTTCCCCTAAT

AATCGGTGCCCCCGATATGGCGTTTCCCCGCATAAACAACATAAGCTTCTGACTCTTACCTCCCTCTCTC

CTACTCCTGCTCGCATCTGCTATAGTGGAGGCCGGAGCAGGAACAGGTTGAACAGTCTACCCTCCCTTAG

CAGGGAACTACTCCCACCCTGGAGCCTCCGTAGACCTAACCATCTTCTCCTTACACCTAGCAGGTGTCTC

CTCTATCTTAGGGGCCATCAATTTCATCACAACAATTATCAATATAAAACCCCCTGCCATAACCCAATAC

CAAACGCCCCTCTTCGTCTGATCCGTCCTAATCACAGCAGTCCTACTTCTCCTATCTCTCCCAGTCCTAG

CTGCTGGCATCACTATACTACTAACAGACCGCAACCTCAACACCACCTTCTTCGACCCCGCCGGAGGAGG

AGACCCCATTCTATACCAACACCTATTCTGATTTTTCGGTCACCCTGAAGTTTATATTCTTATCCTACCA

GGCTTCGGAATAATCTCCCATATTGTAACTTACTACTCCGGAAAAAAAGAACCATTTGGATACATAGGCA

TGGTCTGAGCTATGATATCAATTGGCTTCCTAGGGTTTATCGTGTGAGCACACCATATATTTACAGTAGG

AATAGACGTAGACACACGAGCATATTTCACCTCCGCTACCATAATCATCGCTATCCCCACCGGCGTCAAA

GTATTTAGCTGACTCGCCACACTCCACGGAAGCAATATGAAATGGTCTGCTGCAGTGCTCTGAGCCCTAG

GATTCATCTTTCTTTTCACCGTAGGTGGCCTGACTGGCATTGTATTAGCAAACTCATCACTAGACATCGT

ACTACACGACACGTACTACGTTGTAGCTCACTTCCACTATGTCCTATCAATAGGAGCTGTATTTGCCATC

ATAGGAGGCTTCATTCACTGATTTCCCCTATTCTCAGGCTACACCCTAGACCAAACCTACGCCAAAATCC

ATTTCACTATCATATTCATCGGCGTAAATCTAACTTTCTTCCCACAACACTTTCTCGGCCTATCCGGAAT

GCCCCGACGTTACTCGGACTACCCCGATGCATACACCACATGAAACATCCTATCATCTGTAGGCTCATTC

ATTTCTCTAACAGCAGTAATATTAATAATTTTCATGATTTGAGAAGCCTTCGCTTCGAAGCGAAAAGTCC

TAATAGTAGAAGAACCCTCCATAAACCTGGAGTGACTATATGGATGCCCCCCACCCTACCACACATTCGA

AGAACCCGTATACATAAAATCTAGACAAAAAAGGAAGGAATCGAACCCCCCAAAGCTGGTTTCAAGCCAA

CCCCATGGCCTCCATGACTTTTTCAAAAAGGTATTAGAAAAACCATTTCATAACTTTGTCAAAGTTAAAT

TATAGGCTAAATCCTATATATCTTAATGGCACATGCAGCGCAAGTAGGTCTACAAGACGCTACTTCCCCT

ATCATAGAAGAGCTTATCACCTTTCATGATCACGCCCTCATAATCATTTTCCTTATCTGCTTCCTAGTCC

TGTATGCCCTTTTCCTAACACTCACAACAAAACTAACTAATACTAACATCTCAGACGCTCAGGAAATAGA

AACCGTCTGAACTATCCTGCCCGCCATCATCCTAGTCCTCATCGCCCTCCCATCCCTACGCATCCTTTAC

ATAACAGACGAGGTCAACGATCCCTCCCTTACCATCAAATCAATTGGCCACCAATGGTACTGAACCTACG

AGTACACCGACTACGGCGGACTAATCTTCAACTCCTACATACTTCCCCCATTATTCCTAGAACCAGGCGA

CCTGCGACTCCTTGACGTTGACAATCGAGTAGTACTCCCGATTGAAGCCCCCATTCGTATAATAATTACA

TCACAAGACGTCTTGCACTCATGAGCTGTCCCCACATTAGGCTTAAAAACAGATGCAATTCCCGGACGTC

TAAACCAAACCACTTTCACCGCTACACGACCGGGGGTATACTACGGTCAATGCTCTGAAATCTGTGGAGC

AAACCACAGTTTCATGCCCATCGTCCTAGAATTAATTCCCCTAAAAATCTTTGAAATAGGGCCCGTATTT

ACCCTATAGCACCCCCTCTAGAGCCCACTGTAAAGCTAACTTAGCATTAACCTTTTAAGTTAAAGATTAA

GAGAACCAACACCTCTTTACAGTGAAATGCCCCAACTAAATACTACCGTATGGCCCACCATAATTACCCC

CATACTCCTTACACTATTCCTCATCACCCAACTAAAAATATTAAACACAAACTACCACCTACCTCCCTCA

CCAAAGCCCATAAAAATAAAAAATTATAACAAACCCTGAGAACCAAAATGAACGAAAATCTGTTCGCTTC

ATTCATTGCCCCCACAATCCTAGGCCTACCCGCCGCAGTACTGATCATTCTATTTCCCCCTCTATTGATC

CCCACCTCCAAATATCTCATCAACAACCGACTAATCACCACCCAACAATGACTAATCAAACTAACCTCAA

AACAAATGATAACCATACACAACACTAAAGGACGAACCTGATCTCTTATACTAGTATCCTTAATCATTTT

TATTGCCACAACTAACCTCCTCGGACTCCTGCCTCACTCATTTACACCAACCACCCAACTATCTATAAAC

CTAGCCATGGCCATCCCCTTATGAGCGGGCGCAGTGATTATAGGCTTTCGCTCTAAGATTAAAAATGCCC

TAGCCCACTTCTTACCACAAGGCACACCTACACCCCTTATCCCCATACTAGTTATTATCGAAACCATCAG

CCTACTCATTCAACCAATAGCCCTGGCCGTACGCCTAACCGCTAACATTACTGCAGGCCACCTACTCATG

CACCTAATTGGAAGCGCCACCCTAGCAATATCAACCATTAACCTTCCCTCTACACTTATCATCTTCACAA

TTCTAATTCTACTAACTATCCTAGAAATCGCTGTCGCCTTAATCCAAGCCTACGTTTTCACACTTCTAGT

AAGCCTCTACCTGCACGACAACACATAATGACCCACCAATCACATGCCTATCATATAGTAAAACCCAGCC

CATGACCCCTAACAGGGGCCCTCTCAGCCCTCCTAATGACCTCCGGCCTAGCCATGTGATTTCACTTCCA

CTCCATAACGCTCCTCATACTAGGCCTACTAACCAACACACTAACCATATACCAATGATGGCGCGATGTA

ACACGAGAAAGCACATACCAAGGCCACCACACACCACCTGTCCAAAAAGGCCTTCGATACGGGATAATCC

TATTTATTACCTCAGAAGTTTTTTTCTTCGCAGGATTTTTCTGAGCCTTTTACCACTCCAGCCTAGCCCC

TACCCCCCAATTAGGAGGGCACTGGCCCCCAACAGGCATCACCCCGCTAAATCCCCTAGAAGTCCCACTC

CTAAACACATCCGTATTACTCGCATCAGGAGTATCAATCACCTGAGCTCACCATAGTCTAATAGAAAACA

ACCGAAACCAAATAATTCAAGCACTGCTTATTACAATTTTACTGGGTCTCTATTTTACCCTCCTACAAGC

CTCAGAGTACTTCGAGTCTCCCTTCACCATTTCCGACGGCATCTACGGCTCAACATTTTTTGTAGCCACA

GGCTTCCACGGACTTCACGTCATTATTGGCTCAACTTTCCTCACTATCTGCTTCATCCGCCAACTAATAT

TTCACTTTACATCCAAACATCACTTTGGCTTCGAAGCCGCCGCCTGATACTGGCATTTTGTAGATGTGGT

TTGACTATTTCTGTATGTCTCCATCTATTGATGAGGGTCTTACTCTTTTAGTATAAATAGTACCGTTAAC

TTCCAATTAACTAGTTTTGACAACATTCAAAAAAGAGTAATAAACTTCGCCTTAATTTTAATAATCAACA

CCCTCCTAGCCTTACTACTAATAATTATTACATTTTGACTACCACAACTCAACGGCTACATAGAAAAATC

CACCCCTTACGAGTGCGGCTTCGACCCTATATCCCCCGCCCGCGTCCCTTTCTCCATAAAATTCTTCTTA

GTAGCTATCACCTTCTTATTATTTGATCTAGAAATTGCCCTCCTTTTACCCCTACCATGAGCCCTACAAA

CAACTAACCTGCCACTAATAGTTATGTCATCCCTCTTATTAATCATCATCCTAGCCCTAAGTCTGGCCTA

TGAGTGACTACAAAAAGGATTAGACTGAACCGAATTGGTATATAGTTTAAACAAAACGAATGATTTCGAC

TCATTAAATTATGATAATCATATTTACCAAATGCCCCTCATTTACATAAATATTATACTAGCATTTACCA

TCTCACTTCTAGGAATACTAGTATATCGCTCACACCTCATATCCTCCCTACTATGCCTAGAAGGAATAAT

ACTATCGCTGTTCATTATAGCTACTCTCATAACCCTCAACACCCACTCCCTCTTAGCCAATATTGTGCCT

ATTGCCATACTAGTCTTTGCCGCCTGCGAAGCAGCGGTGGGCCTAGCCCTACTAGTCTCAATCTCCAACA

CATATGGCCTAGACTACGTACATAACCTAAACCTACTCCAATGCTAAAACTAATCGTCCCAACAATTATA

TTACTACCACTGACATGACTTTCCAAAAAACACATAATTTGAATCAACACAACCACCCACAGCCTAATTA

TTAGCATCATCCCTCTACTATTTTTTAACCAAATCAACAACAACCTATTTAGCTGTTCCCCAACCTTTTC

CTCCGACCCCCTAACAACCCCCCTCCTAATACTAACTACCTGACTCCTACCCCTCACAATCATGGCAAGC

CAACGCCACTTATCCAGTGAACCACTATCACGAAAAAAACTCTACCTCTCTATACTAATCTCCCTACAAA

TCTCCTTAATTATAACATTCACAGCCACAGAACTAATCATATTTTATATCTTCTTCGAAACCACACTTAT

CCCCACCTTGGCTATCATCACCCGATGAGGCAACCAGCCAGAACGCCTGAACGCAGGCACATACTTCCTA

TTCTACACCCTAGTAGGCTCCCTTCCCCTACTCATCGCACTAATTTACACTCACAACACCCTAGGCTCAC

TAAACATTCTACTACTCACTCTCACTGCCCAAGAACTATCAAACTCCTGAGCCAACAACTTAATATGACT

AGCTTACACAATAGCTTTTATAGTAAAGATACCTCTTTACGGACTCCACTTATGACTCCCTAAAGCCCAT

GTCGAAGCCCCCATCGCTGGGTCAATAGTACTTGCCGCAGTACTCTTAAAACTAGGCGGCTATGGTATAA

TACGCCTCACACTCATTCTCAACCCCCTGACAAAACACATAGCCTACCCCTTCCTTGTACTATCCCTATG

AGGCATAATTATAACAAGCTCCATCTGCCTACGACAAACAGACCTAAAATCGCTCATTGCATACTCTTCA

ATCAGCCACATAGCCCTCGTAGTAACAGCCATTCTCATCCAAACCCCCTGAAGCTTCACCGGCGCAGTCA

TTCTCATAATCGCCCACGGACTTACATCCTCATTACTATTCTGCCTAGCAAACTCAAACTACGAACGCAC

TCACAGTCGCATCATAATCCTCTCTCAAGGACTTCAAACTCTACTCCCACTAATAGCTTTTTGATGACTT

CTAGCAAGCCTCGCTAACCTCGCCTTACCCCCCACTATTAACCTACTGGGAGAACTCTCTGTGCTAGTAA

CCACGTTCTCCTGATCAAATATCACTCTCCTACTTACAGGACTCAACATACTAGTCACAGCCCTATACTC

CCTCTACATATTTACCACAACACAATGGGGCTCACTCACCCACCACATTAACAACATAAAACCCTCATTC

ACACGAGAAAACACCCTCATGTTCATACACCTATCCCCCATTCTCCTCCTATCCCTCAACCCCGACATCA

TTACCGGGTTTTCCTCTTGTAAATATAGTTTAACCAAAACATCAGATTGTGAATCTGACAACAGAGGCTT

ACGACCCCTTATTTACCGAGAAAGCTCACAAGAACTGCTAACTCATGCCTCCATGTCTAACAACATGGCT

TTCTCAACTTTTAAAGGATAACAGCTATCCATTGGTCTTAGGCCCCAAAAATTTTGGTGCAACTCCAAAT

AAAAGTAATAACCATGCACACTACTATAACCACCCTAACCCTGACTTCCCTAATTCCCCCCATCCTTACC

ACCCTCGTTAACCCTAACAAAAAAAACTCATACCCCCATTATGTAAAATCCATTGTCGCATCCACCTTTA

TTATCAGTCTCTTCCCCACAACAATATTCATGTGCCTAGACCAAGAAGTTATTATCTCGAACTGACACTG

AGCCACAACCCAAACAACCCAGCTCTCCCTAAGCTTCAAACTAGACTACTTCTCCATAATATTCATCCCT

GTAGCATTGTTCGTTACATGGTCCATCATAGAATTCTCACTGTGATATATAAACTCAGACCCAAACATTA

ATCAGTTCTTCAAATATCTACTCATCTTCCTAATTACCATACTAATCTTAGTTACCGCTAACAACCTATT

TCAACTGTTCATCGGCTGAGAGGGCGTAGGAATTATATCCTTCTTGCTCATCAGTTGATGATACGCCCGA

GCAGATGCCAACACAGCAGCCATTCAAGCAATCCTATACAACCGTATCGGCGATATCGGTTTCATCCTCG

CCTTAGCATGATTTATCCTACACTCCAACTCATGAGACCCACAACAAATAGCCCTTCTAAACGCTAATCC

AAGCCTCACCCCACTACTAGGCCTCCTCCTAGCAGCAGCAGGCAAATCAGCCCAATTAGGTCTCCACCCC

TGACTCCCCTCAGCCATAGAAGGCCCCACCCCAGTCTCAGCCCTACTCCACTCAAGCACTATAGTTGTAG

CAGGAATCTTCTTACTCATCCGCTTCCACCCCCTAGCAGAAAATAGCCCACTAATCCAAACTCTAACACT

ATGCTTAGGCGCTATCACCACTCTGTTCGCAGCAGTCTGCGCCCTTACACAAAATGACATCAAAAAAATC

GTAGCCTTCTCCACTTCAAGTCAACTAGGACTCATAATAGTTACAATCGGCATCAACCAACCACACCTAG

CATTCCTGCACATCTGTACCCACGCCTTCTTCAAAGCCATACTATTTATGTGCTCCGGGTCCATCATCCA

CAACCTTAACAATGAACAAGATATTCGAAAAATAGGAGGACTACTCAAAACCATACCTCTCACTTCAACC

TCCCTCACCATTGGCAGCCTAGCATTAGCAGGAATACCTTTCCTCACAGGTTTCTACTCCAAAGACCACA

TCATCGAAACCGCAAACATATCATACACAAACGCCTGAGCCCTATCTATTACTCTCATCGCTACCTCCCT

GACAAGCGCCTATAGCACTCGAATAATTCTTCTCACCCTAACAGGTCAACCTCGCTTCCCCACCCTTACT

AACATTAACGAAAATAACCCCACCCTACTAAACCCCATTAAACGCCTGGCAGCCGGAAGCCTATTCGCAG

GATTTCTCATTACTAACAACATTTCCCCCGCATCCCCCTTCCAAACAACAATCCCCCTCTACCTAAAACT

CACAGCCCTCGCTGTCACTTTCCTAGGACTTCTAACAGCCCTAGACCTCAACTACCTAACCAACAAACTT

AAAATAAAATCCCCACTATGCACATTTTATTTCTCCAACATACTCGGATTCTACCCTAGCATCACACACC

GCACAATCCCCTATCTAGGCCTTCTTACGAGCCAAAACCTGCCCCTACTCCTCCTAGACCTAACCTGACT

AGAAAAGCTATTGCCTAAAACAATTTCACAGCACCAAATCTCCACCTCCATCATCACCTCAACCCAAAAA

GGCATAATTAAACTTTACTTCCTCTCTTTCTTCTTCCCACTCATCCTAACCCTACTCCTAATCACATAAC

CTATTCCCCCGAGCAATCTCAATTACAATATATACACCAACAAACAATGTTCAACCAGTAACTACTACTA

ATCAACGCCCATAATCATACAAAGCCCCCGCACCAATAGGATCCTCCCGAATCAACCCTGACCCCTCTCC

TTCATAAATTATTCAGCTTCCTACACTATTAAAGTTTACCACAACCACCACCCCATCATACTCTTTCACC

CACAGCACCAATCCTACCTCCATCGCTAACCCCACTAAAACACTCACCAAGACCTCAACCCCTGACCCCC

ATGCCTCAGGATACTCCTCAATAGCCATCGCTGTAGTATATCCAAAGACAACCATCATTCCCCCTAAATA

AATTAAAAAAACTATTAAACCCATATAACCTCCCCCAAAATTCAGAATAATAACACACCCGACCACACCG

CTAACAATCAATACTAAACCCCCATAAATAGGAGAAGGCTTAGAAGAAAACCCCACAAACCCCATTACTA

AACCCACACTCAACAGAAACAAAGCATACATCATTATTCTCGCACGGACTACAACCACGACCAATGATAT

GAAAAACCATCGTTGTATTTCAACTACAAGAACACCAATGACCCCAATACGCAAAATTAACCCCCTAATA

AAATTAATTAACCACTCATTCATCGACCTCCCCACCCCATCCAACATCTCCGCATGATGAAACTTCGGCT

CACTCCTTGGCGCCTGCCTGATCCTCCAAATCACCACAGGACTATTCCTAGCCATGCACTACTCACCAGA

CGCCTCAACCGCCTTTTCATCAATCGCCCACATCACTCGAGACGTAAATTATGGCTGAATCATCCGCTAC

CTTCACGCCAATGGCGCCTCAATATTCTTTATCTGCCTCTTCCTACACATCGGGCGAGGCCTATATTACG

GATCATTTCTCTACTCAGAAACCTGAAACATCGGCATTATCCTCCTGCTTGCAACTATAGCAACAGCCTT

CATAGGCTATGTCCTCCCGTGAGGCCAAATATCATTCTGAGGGGCCACAGTAATTACAAACTTACTATCC

GCCATCCCATACATTGGGACAGACCTAGTTCAATGAATCTGAGGAGGCTACTCAGTAGACAGTCCCACCC

TCACACGATTCTTTACCTTTCACTTCATCTTGCCCTTCATTATTGCAGCCCTAGCAGCACTCCACCTCCT

ATTCTTGCACGAAACGGGATCAAACAACCCCCTAGGAATCACCTCCCATTCCGATAAAATCACCTTCCAC

CCTTACTACACAATCAAAGACGCCCTCGGCTTACTTCTCTTCCTTCTCTCCTTAATGACATTAACACTAT

TCTCACCAGACCTCCTAGGCGACCCAGACAATTATACCCTAGCCAACCCCTTAAACACCCCTCCCCACAT

CAAGCCCGAATGATATTTCCTATTCGCCTACACAATTCTCCGATCCGTCCCTAACAAACTAGGAGGCGTC

CTTGCCCTATTACTATCCATCCTCATCCTAGCAATAATCCCCATCCTCCATATATCCAAACAACAAAGCA

TAATATTTCGCCCACTAAGCCAATCACTTTATTGACTCCTAGCCGCAGACCTCCTCGTTCTAACCTGAAT

CGGAGGACAACCAGTAAGCTACCCTTTTACCATCATTGGACAAGTAGCATCCGTACTATACTTCACAACA

ATCCTAATCCTAATACCAACTATCTCCCTAATTGAAAACAAAATACTCAAATGGGCCTGTCCTTGTAGTA

TAAACTAATACACCAGTCTTGTAAACCGGAGATGAAAACCTTTTTCCAAGGACAAATCAGAGAAAAAGTC

TTTAACTCCACCATTAGCACCCAAAGCTAAGATTCTAATTTAAACTATTCTCTGTTCTTTCATGGGGAAG

CAGATTTGGGTACCACCCAAGTATTGACTCACCCATCAACAACCGCTATGTATTTCGTACATTACTGCCA

GCCACCATGAATATTGTACGGTACCATAAATACTTGACCACCTGTAGTACATAAAAACCCAATCCACATC

AACCCCCCCCCCCCATGCTTACAAGCAAGTACAGCAACCAACCCTCAACTATCACACATCAACTGCAACT

CCAAAGCCACCTCTCACCCACTAGGATACCAACAAACCTACCCACCCTTAACAGTACATAGTACATAGAG

CCATTTACCGTACATAGCACATTACAGTCAAATCCCTTCTCGTCCCCATGGATGACCCCCCTCAGATAGG

GGTCCCTTGACCACCATCCTCCGTGAAATCAATATCCCGCACAAGAGTGCTACTCTCCTCGCTCCGGGCC

CATAACACTTGGGGGTAGCTAAAGTGAACTGTATCCGACATCTGGTTCCTACTTCAGGGCCATAAAGCCT

AAATAGCCCACACGTTCCCCTTAAATAAGACATCACGATG

>MT135513.1 Homo sapiens isolate NH29 mitochondrion, complete genome

GATCACAGGTCTATCACCCTATTAACCACTCACGGGAGCTCTCCATGCATTTGGTATTTTCGTCTGGGGG

GTGTGCACGCGATAGCATTGCGAGACGCTGGAGCCGGAGCACCCTATGTCGCAGTATCTGTCTTTGATTC

CTGCCCCATCCTATTATTTATCGCACCTACGTTCAATATTACAGGCGAACATACTTACTAAAGTGTGTTA

ATTAATTAATGCTTGTAGGACATAATAATAACAATTGAATGTCTGCACAGCCGCTTTCCACACAGACATC

ATAACAAAAAATTTCCACCAAACCCCCCCCTCCCCCCGCTTCTGGCCACAGCACTTAAACACATCTCTGC

CAAACCCCAAAAACAAAGAACCCTAACACCAGCCTAACCAGATTTCAAATTTTATCTTTTGGCGGTATGC

ACTTTTAACAGTCACCCCCCAACTAACACATTATTTTCCCCTCCCACTCCCATACTACTAATCTCATCAA

TACAACCCCCGCCCATCCTACCCAGCACACACACCGCTGCTAACCCCATACCCCGAACCAACCAAACCCC

AAAGACACCCCCCACAGTTTATGTAGCTTACCTCCTCAAAGCAATACACTGAAAATGTTTAGACGGGCTC

ACATCACCCCATAAACAAATAGGTTTGGTCCTAGCCTTTCTATTAGCTCTTAGTAAGATTACACATGCAA

GCATCCCCGTTCCAGTGAGTTCACCCTCTAAATCACCACGATCAAAAGGGACAAGCATCAAGCACGCAGC

AATGCAGCTCAAAACGCTTAGCCTAGCCACACCCCCACGGGAAACAGCAGTGATTAACCTTTAGCAATAA

ACGAAAGTTTAACTAAGCTATACTAACCCCAGGGTTGGTCAATTTCGTGCCAGCCACCGCGGTCACACGA

TTAACCCAAGTCAATAGAAGCCGGCGTAAAGAGTGTTTTAGATCACCCCCTCCCCAATAAAGCTAAAACT

CACCTGAGTTGTAAAAAACTCCAGTTGACACAAAATAGACTACGAAAGTGGCTTTAACATATCTGAACAC

ACAATAGCTAAGACCCAAACTGGGATTAGATACCCCACTATGCTTAGCCCTAAACCTCAACAGTTAAATC

AACAAAACTGCTCGCCAGAACACTACGAGCCACAGCTTAAAACTCAAAGGACCTGGCGGTGCTTCATATC

CCTCTAGAGGAGCCTGTTCTGTAATCGATAAACCCCGATCAACCTCACCACCTCTTGCTCAGCCTATATA

CCGCCATCTTCAGCAAACCCTGATGAAGGCTACAAAGTAAGCGCAAGTACCCACGTAAAGACGTTAGGTC

AAGGTGTAGCCCATGAGGTGGCAAGAAATGGGCTACATTTTCTACCCCAGAAAACTACGATAGCCCTTAT

GAAACTTAAGGGTCGAAGGTGGATTTAGCAGTAAACTGAGAGTAGAGTGCTTAGTTGAACAGGGCCCTGA

AGCGCGTACACACCGCCCGTCACCCTCCTCAAGTATACTTCAAAGGACATTTAACTAAAACCCCTACGCA

TTTATATAGAGGAGACAAGTCGTAACATGGTAAGTGTACTGGAAAGTGCACTTGGACGAACCAGAGTGTA

GCTTAACACAAAGCACCCAACTTACACTTAGGAGATTTCAACTTAACTTGACCGCTCTGAGCTAAACCTA

GCCCCAAACCCACTCCACCTTACTACCAGACAACCTTAGCCAAACCATTTACCCAAATAAAGTATAGGCG

ATAGAAATTGAAACCTGGCGCAATAGATATAGTACCGCAAGGGAAAGATGAAAAATTATAACCAAGCATA

ATATAGCAAGGACTAACCCCTATACCTTCTGCATAATGAATTAACTAGAAATAACTTTGCAAGGAGAGCC

AAAGCTAAGACCCCCGAAACCAGACGAGCTACCTAAGAACAGCTAAAAGAGCACACCCGTCTATGTAGCA

AAATAGTGGGAAGATTTATAGGTAGAGGCGACAAACCTACCGAGCCTGGTGATAGCTGGTTGTCCAAGAT

AGAATCTTAGTTCAACTTTAAATTTGCCCACAGAACCCTCTAAATCCCCTTGTAAATTTAACTGTTAGTC

CAAAGAGGAACAGCTCTTTGGACACTAGGAAAAAACCTTGTAGAGAGAGTAAAAAATTTAACACCCATAG

TAGGCCTAAAAGCAGCCACCAATTAAGAAAGCGTTCAAGCTCAACACCCACTACCTAAAAAATCCCAAAC

ATATAACTGAACTCCTCACACCCAATTGGACCAATCTATCACCCTATAGAAGAACTAATGTTAGTATAAG

TAACATGAAAACATTCTCCTCCGCATAAGCCTGCGTCAGATTAAAACACTGAACTGACAATTAACAGCCC

AATATCTACAATCAACCAACAAGTCATTATTACCCTCACTGTCAACCCAACACAGGCATGCTCATAAGGA

AAGGTTAAAAAAAGTAAAAGGAACTCGGCAAATCTTACCCCGCCTGTTTACCAAAAACATCACCTCTAGC

ATCACCAGTATTAGAGGCACCGCCTGCCCAGTGACACATGTTTAACGGCCGCGGTACCCTAACCGTGCAA

AGGTAGCATAATCACTTGTTCCTTAAATAGGGACCTGTATGAATGGCTCCACGAGGGTTCAGCTGTCTCT

TACTTTTAACCAGTGAAATTGACCTGCCCGTGAAGAGGCGGGCATGACACAGCAAGACGAGAAGACCCTA

TGGAGCTTTAATTTATTAATGCAAACAGTACCTAACAAACCCACAGGTCCTAAACTACCAAACCTGCATT

AAAAATTTCGGTTGGGGCGACCTCGGAGCAGAACCCAACCTCCGAGCAGTACATGCTAAGACTTCACCAG

TCAAAGCGAACTACTATACTCAATTGATCCAATAACTTGACCAACGGAACAAGTTACCCTAGGGATAACA

GCGCAATCCTATTCTAGAGTCCATATCAACAATAGGGTTTACGACCTCGATGTTGGATCAGGACATCCCG

ATGGTGCAGCCGCTATTAAAGGTTCGTTTGTTCAACGATTAAAGTCCTACGTGATCTGAGTTCAGACCGG

AGTAATCCAGGTCGGTTTCTATCTACNTTCAAATTCCTCCCTGTACGAAAGGACAAGAGAAATAAGGCCT

ACTTCACAAAGCGCCTTCCCCCGTAAATGATATCATCTCAACTTAGTATTATACCCACACCCACCCAAGA

ACAGGGTTTGTTAAGATGGCAGAGCCCGGTAATCGCATAAAACTTAAAACTTTACAGTCAGAGGTTCAAT

TCCTCTTCTTAACAACATACCCATGGCCAACCTCCTACTCCTCATTGTACCCATTCTAATCGCAATGGCA

TTCCTAATGCTTACCGAACGAAAAATTCTAGGCTATATACAACTACGCAAAGGCCCCAACGTTGTAGGCC

CCTACGGGCTACTACAACCCTTCGCTGACGCCATAAAACTCTTCACCAAAGAGCCCCTAAAACCCGCCAC

ATCTACCATCACCCTCTACATCACCGCCCCGACCTTAGCTCTCACCATCGCTCTTCTACTATGAACCCCC

CTCCCCATACCCAACCCCCTGGTCAACCTCAACCTAGGCCTCCTATTTATTCTAGCCACCTCTAGCCTAG

CCGTTTACTCAATCCTCTGATCAGGGTGAGCATCAAACTCAAACTACGCCCTGATCGGCGCACTGCGAGC

AGTAGCCCAAACAATCTCATATGAAGTCACCCTAGCCATCATTCTACTATCAACATTACTAATAAGTGGC

TCCTTTAACCTCTCCACCCTTATCACAACACAAGAACACCTCTGATTACTCCTGCCATCATGACCCTTGG

CCATAATATGATTTATCTCCACACTAGCAGAGACCAACCGAACCCCCTTCGACCTTGCCGAAGGGGAGTC

CGAACTAGTCTCAGGCTTCAACATCGAATACGCCGCAGGCCCCTTCGCCCTATTCTTCATAGCCGAATAC

ACAAACATTATTATAATAAACACCCTCACCACTACAATCTTCCTAGGAACAACATATGACGCACTCTCCC

CTGAACTCTACACAACATATTTTGTCACCAAGACCCTACTTCTAACCTCCCTGTTCTTATGAATTCGAAC

AGCATACCCCCGATTCCGCTACGACCAACTCATACACCTCCTATGAAAAAACTTCCTACCACTCACCCTA

GCATTACTTATATGATATGTCTCCATACCCATTACAATCTCCAGCATTCCCCCTCAAACCTAAGAAATAT

GTCTGATAAAAGAGTTACTTTGATAGAGTAAATAATAGGAGCTTAAACCCCCTTATTTCTAGGACTATGA

GAATCGAACCCATCCCTGAGAATCCAAAATTCTCCGTGCCACCTATCACACCCCATCCTAAAGTAAGGTC

AGCTAAATAAGCTATCGGGCCCATACCCCGAAAATGTTGGTTATACCCTTCCCGTACTAATTAATCCCCT

GGCCCAACCCGTCATCTACTCTACCATCTTTGCAGGCACACTCATCACAGCGCTAAGCTCGCACTGATTT

TTTACCTGAGTAGGCCTAGAAATAAACATGCTAGCTTTTATTCCAGTTCTAACCAAAAAAATAAACCCTC

GTTCCACAGAAGCTGCCATCAAGTATTTCCTCACGCAAGCAACCGCATCCATAATCCTTCTAATAGCTAT

CCTCTTCAACAATATACTCTCCGGACAATGAACCATAACCAATACTACCAATCAATACTCATCATTAATA

ATCATAATGGCTATAGCAATAAAACTAGGAATAGCCCCCTTTCACTTCTGAGTCCCAGAGGTTACCCAAG

GCACCCCTCTGACATCCGGCCTGCTTCTTCTCACATGACAAAAACTAGCCCCCATCTCAATCATATACCA

AATCTCTCCCTCACTAAACGTAAGCCTTCTCCTCACTCTCTCAATCTTATCCATCATAGCAGGCAGTTGA

GGTGGATTAAACCAAACCCAGCTACGCAAAATCTTAGCATACTCCTCAATTACCCACATAGGATGAATAA

TAGCAGTTCTACCGTACAACCCTAACATAACCATTCTTAATTTAACTATTTATATTATCCTAACTACTAC

CGCATTCCTACTACTCAACTTAAACTCCAGCACCACGACCCTACTACTATCTCGCACCTGAAACAAGCTA

ACATGACTAACACCCTTAATTCCATCCACCCTCCTCTCCCTAGGAGGCCTGCCCCCGCTAACCGGCTTTT

TGCCCAAATGGGCCATTATCGAAGAATTCACAAAAAACAATAGCCTCATCATCCCCACCATCATAGCCAC

CATCACCCTCCTTAACCTCTACTTCTACCTACGCCTAATCTACTCCACCTCAATCACACTACTCCCCATA

TCTAACAACGTAAAAATAAAATGACAGTTTGAACATACAAAACCCACCCCATTCCTCCCCACACTCATCG

CCCTCACCACGCTACTCCTACCTATCTCCCCTTTTATACTAATAATCTTATAGAAATTTAGGTTAAATAC

AGACCAAGAGCCTTCAAAGCCCTCAGTAAGTTGCAATACTTAATTTCTGTAACAGCTAAGGACTGCAAAA

CCCCACTCTGCATCAACTGAACGCAAATCAGCCACTTTAATTAAGCTAAGCCCTTACTAGACCAATGGGA

CTTAAACCCACAAACACTTAGTTAACAGCTAAGCACCCTAATCAACTGGCTTCAATCTACTTCTCCCGCC

GCCGGGAAAAAAGGCGGGAGAAGCCCCGGCAGGTTTGAAGCTGCTTCTTCGAATTTGCAATTCAATATGA

AAATCACCTCGGAGCTGGTAAAAAGAGGCCTAACCCCTGTCTTTAGATTTACAGTCCAATGCTTCACTCA

GCCATTTTACCTCACCCCCACTGATGTTCGCCGACCGTTGACTATTCTCTACAAACCACAAAGACATTGG

AACACTATACCTATTATTCGGCGCATGAGCTGGAGTCCTAGGCACAGCTCTAAGCCTCCTTATTCGAGCC

GAGCTGGGCCAGCCAGGCAACCTTCTAGGTAACGACCACATCTACAACGTTATCGTCACAGCCCATGCAT

TTGTAATAATCTTCTTCATAGTAATACCCATCATAATCGGAGGCTTTGGCAACTGACTAGTTCCCCTAAT

AATCGGTGCCCCCGATATGGCGTTTCCCCGCATAAACAACATAAGCTTCTGACTCTTACCTCCCTCTCTC

CTACTCCTGCTCGCATCTGCTATAGTGGAGGCCGGAGCAGGAACAGGTTGAACAGTCTACCCTCCCTTAG

CAGGGAACTACTCCCACCCTGGAGCCTCCGTAGACCTAACCATCTTCTCCTTACACCTAGCAGGTGTCTC

CTCTATCTTAGGGGCCATCAATTTCATCACAACAATTATCAATATAAAACCCCCTGCCATAACCCAATAC

CAAACGCCCCTCTTCGTCTGATCCGTCCTAATCACAGCAGTCCTACTTCTCCTATCTCTCCCAGTCCTAG

CTGCTGGCATCACTATACTACTAACAGACCGCAACCTCAACACCACCTTCTTCGACCCCGCCGGAGGAGG

AGACCCCATTCTATACCAACACCTATTCTGATTTTTCGGTCACCCTGAAGTTTATATTCTTATCCTACCA

GGCTTCGGAATAATCTCCCATATTGTAACTTACTACTCCGGAAAAAAAGAACCATTTGGATACATAGGCA

TGGTCTGAGCTATGATATCAATTGGCTTCCTAGGGTTTATCGTGTGAGCACACCATATATTTACAGTAGG

AATAGACGTAGACACACGAGCATATTTCACCTCCGCTACCATAATCATCGCTATCCCCACCGGCGTCAAA

GTATTTAGCTGACTCGCCACACTCCACGGAAGCAATATGAAATGATCTGCTGCAGTGCTCTGAGCCCTAG

GATTCATCTTTCTTTTCACCGTAGGTGGCCTGACTGGCATTGTATTAGCAAACTCATCACTAGACATCGT

ACTACACGACACGTACTACGTTGTAGCTCACTTCCACTATGTCCTATCAATAGGAGCTGTATTTGCCATC

ATAGGAGGCTTCATTCACTGATTTCCCCTATTCTCAGGCTACACCCTAGACCAAACCTACGCCAAAATCC

ATTTCACTATCATATTCATCGGCGTAAATCTAACTTTCTTCCCACAACACTTTCTCGGCCTATCCGGAAT

GCCCCGACGTTACTCGGACTACCCCGATGCATACACCACATGAAACATCCTATCATCTGTAGGCTCATTC

ATTTCTCTAACAGCAGTAATATTAATAATTTTCATGATTTGAGAAGCCTTCGCTTCGAAGCGAAAAGTCC

TAATAGTAGAAGAACCCTCCATAAACCTGGAGTGACTATATGGATGCCCCCCACCCTACCACACATTCGA

AGAACCCGTATACATAAAATCTAGACAAAAAAGGAAGGAATCGAACCCCCCAAAGCTGGTTTCAAGCCAA

CCCCATGGCCTCCATGACTTTTTCAAAAAGGTATTAGAAAAACCATTTCATAACTTTGTCAAAGTTAAAT

TATAGGCTAAATCCTATATATCTTAATGGCACATGCAGCGCAAGTAGGTCTACAAGACGCTACTTCCCCT

ATCATAGAAGAGCTTATCACCTTTCATGATCACGCCCTCATAATCATTTTCCTTATCTGCTTCCTAGTCC

TGTATGCCCTTTTCCTAACACTCACAACAAAACTAACTAATACTAACATCTCAGACGCTCAGGAAATAGA

AACCGTCTGAACTATCCTGCCCGCCATCATCCTAGTCCTCATCGCCCTCCCATCCCTACGCATCCTTTAC

ATAACAGACGAGGTCAACGATCCCTCCCTTACCATCAAATCAATTGGCCACCAATGGTACTGAACCTACG

AGTACACCGACTACGGCGGACTAATCTTCAACTCCTACATACTTCCCCCATTATTCCTAGAACCAGGCGA

CCTGCGACTCCTTGACGTTGACAATCGAGTAGTACTCCCGATTGAAGCCCCCATTCGTATAATAATTACA

TCACAAGACGTCTTGCACTCATGAGCTGTCCCCACATTAGGCTTAAAAACAGATGCAATTCCCGGACGTC

TAAACCAAACCACTTTCACCGCTACACGACCGGGGGTATACTACGGTCAATGCTCTGAAATCTGTGGAGC

AAACCACAGTTTCATGCCCATCGTCCTAGAATTAATTCCCCTAAAAATCTTTGAAATAGGGCCCGTATTT

ACCCTATAGCACCCCCTCTAGAGCCCACTGTAAAGCTAACTTAGCATTAACCTTTTAAGTTAAAGATTAA

GAGAACCAACACCTCTTTACAGTGAAATGCCCCAACTAAATACTACCGTATGGCCCACCATAATTACCCC

CATACTCCTTACACTATTCCTCATCACCCAACTAAAAATATTAAACACAAACTACCACCTACCTCCCTCA

CCAAAGCCCATAAAAATAAAAAATTATAACAAACCCTGAGAACCAAAATGAACGAAAATCTGTTCGCTTC

ATTCATTGCCCCCACAATCCTAGGCCTACCCGCCGCAGTACTGATCATTCTATTTCCCCCTCTATTGATC

CCCACCTCCAAGTATCTCATCAACAACCGACTAATCACCACCCAACAATGACTAATCAAACTAACCTCAA

AACAAATGATAACCATACACAACACTAAAGGACGAACCTGATCTCTTATACTAGTATCCTTAATCATTTT

TATTGCCACAACTAACCTCCTCGGACTCCTGCCTCACTCATTTACACCAACCACCCAACTATCTATAAAC

CTAGCCATGGCCATCCCCTTATGAGCGGGCGCAGTGATTATAGGCTTTCGCTCTAAGATTAAAAATGCCC

TAGCCCACTTCTTACCACAAGGCACACCTACACCCCTTATCCCCATACTAGTTATTATCGAAACCATCAG

CCTACTCATTCAACCAATAGCCCTGGCCGTACGCCTAACCGCTAACATTACTGCAGGCCACCTACTCATG

CACCTAATTGGAAGCGCCACCCTAGCAATATCAACCATTAACCTTCCCTCTACACTTATCATCTTCACAA

TTCTAATTCTACTAACTATCCTAGAAATCGCTGTCGCCTTAATCCAAGCCTACGTTTTCACACTTCTAGT

AAGCCTCTACCTGCACGACAACACATAATGACCCACCAATCACATGCCTATCATATAGTAAAACCCAGCC

CATGACCCCTAACAGGGGCCCTCTCAGCCCTCCTAATGACCTCCGGCCTAGCCATGTGATTTCACTTCCA

CTCCATAACGCTCCTCATACTAGGCCTACTAACCAACACACTAACCATATACCAATGATGGCGCGATGTA

ACACGAGAAAGCACATACCAAGGCCACCACACACCACCTGTCCAAAAAGGCCTTCGATACGGGATAATCC

TATTTATTACCTCAGAAGTTTTTTTCTTCGCAGGATTTTTCTGAGCCTTTTACCACTCCAGCCTAGCCCC

TACCCCCCAATTAGGAGGGCACTGGCCCCCAACAGGCATCACCCCGCTAAATCCCCTAGAAGTCCCACTC

CTAAACACATCCGTATTACTCGCATCAGGAGTATCAATCACCTGAGCTCACCATAGTCTAATAGAAAACA

ACCGAAACCAAATAATTCAAGCACTGCTTATTACAATTTTACTGGGTCTCTATTTTACCCTCCTACAAGC

CTCAGAGTACTTCGAGTCTCCCTTCACCATTTCCGACGGCATCTACGGCTCAACATTTTTTGTAGCCACA

GGCTTCCACGGACTTCACGTCATTATTGGCTCAACTTTCCTCACTATCTGCTTCATCCGCCAACTAATAT

TTCACTTTACATCCAAACATCACTTTGGCTTCGAAGCCGCCGCCTGATACTGGCATTTTGTAGATGTGGT

TTGACTATTTCTGTATGTCTCCATCTATTGATGAGGGTCTTACTCTTTTAGTATAAATAGTACCGTTAAC

TTCCAATTAACTAGTTTTGACAACATTCAAAAAAGAGTAATAAACTTCGCCTTAATTTTAATAATCAACA

CCCTCCTAGCCTTACTACTAATAATTATTACATTTTGACTACCACAACTCAACGGCTACATAGAAAAATC

CACCCCTTACGAGTGCGGCTTCGACCCTATATCCCCCGCCCGCGTCCCTTTCTCCATAAAATTCTTCTTA

GTAGCTATCACCTTCTTATTATTTGATCTAGAAATTGCCCTCCTTTTACCCCTACCATGAGCCCTACAAA

CAACTAACCTGCCACTAATAGTTATGTCATCCCTCTTATTAATCATCATCCTAGCCCTAAGTCTGGCCTA

TGAGTGACTACAAAAAGGATTAGACTGAACCGAATTGGTATATAGTTTAAACAAAACGAATGATTTCGAC

TCATTAAATTATGATAATCATATTTACCAAATGCCCCTCATTTACATAAATATTATACTAGCATTTACCA

TCTCACTTCTAGGAATACTAGTATATCGCTCACACCTCATATCCTCCCTACTATGCCTAGAAGGAATAAT

ACTATCGCTGTTCATTATAGCTACTCTCATAACCCTCAACACCCACTCCCTCTTAGCCAATATTGTGCCT

ATTGCCATACTAGTCTTTGCCGCCTGCGAAGCAGCGGTGGGCCTAGCCCTACTAGTCTCAATCTCCAACA

CATATGGCCTAGACTACGTACATAACCTAAACCTACTCCAATGCTAAAACTAATCGTCCCAACAATTATA

TTACTACCACTGACATGACTTTCCAAAAAACACATAATTTGAATCAACACAACCACCCACAGCCTAATTA

TTAGCATCATCCCTCTACTATTTTTTAACCAAATCAACAACAACCTATTTAGCTGTTCCCCAACCTTTTC

CTCCGACCCCCTAACAACCCCCCTCCTAATACTAACTACCTGACTCCTACCCCTCACAATCATGGCAAGC

CAACGCCACTTATCCAGTGAACCACTATCACGAAAAAAACTCTACCTCTCTATACTAATCTCCCTACAAA

TCTCCTTAATTATAACATTCACAGCCACAGAACTAATCATATTTTATATCTTCTTCGAAACCACACTTAT

CCCCACCTTGGCTATCATCACCCGATGAGGCAACCAGCCAGAACGCCTGAACGCAGGCACATACTTCCTA

TTCTACACCCTAGTAGGCTCCCTTCCCCTACTCATCGCACTAATTTACACTCACAACACCCTAGGCTCAC

TAAACATTCTACTACTCACTCTCACTGCCCAAGAACTATCAAACTCCTGAGCCAACAACTTAATATGACT

AGCTTACACAATAGCTTTTATAGTAAAGATACCTCTTTACGGACTCCACTTATGACTCCCTAAAGCCCAT

GTCGAAGCCCCCATCGCTGGGTCAATAGTACTTGCCGCAGTACTCTTAAAACTAGGCGGCTATGGTATAA

TACGCCTCACACTCATTCTCAACCCCCTGACAAAACACATAGCCTACCCCTTCCTTGTACTATCCCTATG

AGGCATAATTATAACAAGCTCCATCTGCCTACGACAAACAGACCTAAAATCGCTCATTGCATACTCTTCA

ATCAGCCACATAGCCCTCGTAGTAACAGCCATTCTCATCCAAACCCCCTGAAGCTTCACCGGCGCAGTCA

TTCTCATAATCGCCCACGGACTTACATCCTCATTACTATTCTGCCTAGCAAACTCAAACTACGAACGCAC

TCACAGTCGCATCATAATCCTCTCTCAAGGACTTCAAACTCTACTCCCACTAATAGCTTTTTGATGACTT

CTAGCAAGCCTCGCTAACCTCGCCTTACCCCCCACTATTAACCTACTGGGAGAACTCTCTGTGCTAGTAA

CCACGTTCTCCTGATCAAATATCACTCTCCTACTTACAGGACTCAACATACTAGTCACAGCCCTATACTC

CCTCTACATATTTACCACAACACAATGGGGCTCACTCACCCACCACATTAACAACATAAAACCCTCATTC

ACACGAGAAAACACCCTCATGTTCATACACCTATCCCCCATTCTCCTCCTATCCCTCAACCCCGACATCA

TTACCGGGTTTTCCTCTTGTAAATATAGTTTAACCAAAACATCAGATTGTGAATCTGACAACAGAGGCTT

ACGACCCCTTATTTACCGAGAAAGCTCACAAGAACTGCTAACTCATGCCTCCATGTCTAACAACATGGCT

TTCTCAACTTTTAAAGGATAACAGCTATCCATTGGTCTTAGGCCCCAAAAATTTTGGTGCAACTCCAAAT

AAAAGTAATAACCATGCACACTACTATAACCACCCTAACCCTGACTTCCCTAATTCCCCCCATCCTTACC

ACCCTCGTTAACCCTAACAAAAAAAACTCATACCCCCATTATGTAAAATCCATTGTCGCATCCACCTTTA

TTATCAGTCTCTTCCCCACAACAATATTCATGTGCCTAGACCAAGAAGTTATTATCTCGAACTGACACTG

AGCCACAACCCAAACAACCCAGCTCTCCCTAAGCTTCAAACTAGACTACTTCTCCATAATATTCATCCCT

GTAGCATTGTTCGTTACATGGTCCATCATAGAATTCTCACTGTGATATATAAACTCAGACCCAAACATTA

ATCAGTTCTTCAAATATCTACTCATCTTCCTAATTACCATACTAATCTTAGTTACCGCTAACAACCTATT

CCAACTGTTCATCGGCTGAGAGGGCGTAGGAATTATATCCTTCTTGCTCATCAGTTGATGATACGCCCGA

GCAGATGCCAACACAGCAGCCATTCAAGCAATCCTATACAACCGTATCGGCGATATCGGTTTCATCCTCG

CCTTAGCATGATTTATCCTACACTCCAACTCATGAGACCCACAACAAATAGCCCTTCTAAACGCTAATCC

AAGCCTCACCCCACTACTAGGCCTCCTCCTAGCAGCAGCAGGCAAATCAGCCCAATTAGGTCTCCACCCC

TGACTCCCCTCAGCCATAGAAGGCCCCACCCCAGTCTCAGCCCTACTCCACTCAAGCACTATAGTTGTAG

CAGGAATCTTCTTACTCATCCGCTTCCACCCCCTAGCAGAAAATAGCCCACTAATCCAAACTCTAACACT

ATGCTTAGGCGCTATCACCACTCTGTTCGCAGCAGTCTGCGCCCTTACACAAAATGACATCAAAAAAATC

GTAGCCTTCTCCACTTCAAGTCAACTAGGACTCATAATAGTTACAATCGGCATCAACCAACCACACCTAG

CATTCCTGCACATCTGTACCCACGCCTTCTTCAAAGCCATACTATTTATGTGCTCCGGGTCCATCATCCA

CAACCTTAACAATGAACAAGATATTCGAAAAATAGGAGGACTACTCAAAACCATACCTCTCACTTCAACC

TCCCTCACCATTGGCAGCCTAGCATTAGCAGGAATACCTTTCCTCACAGGTTTCTACTCCAAAGACCACA

TCATCGAAACCGCAAACATATCATACACAAACGCCTGAGCCCTATCTATTACTCTCATCGCTACCTCCCT

GACAAGCGCCTATAGCACTCGAATAATTCTTCTCACCCTAACAGGTCAACCTCGCTTCCCCACCCTTACT

AACATTAACGAAAATAACCCCACCCTACTAAACCCCATTAAACGCCTGGCAGCCGGAAGCCTATTCGCAG

GATTTCTCATTACTAACAACATTTCCCCCGCATCCCCCTTCCAAACAACAATCCCCCTCTACCTAAAACT

CACAGCCCTCGCTGTCACTTTCCTAGGACTTCTAACAGCCCTAGACCTCAACTACCTAACCAACAAACTT

AAAATAAAATCCCCACTATGCACATTTTATTTCTCCAACATACTCGGATTCTACCCTAGCATCACACACC

GCACAATCCCCTATCTAGGCCTTCTTACGAGCCAAAACCTGCCCCTACTCCTCCTAGACCTAACCTGACT

AGAAAAGCTATTGCCTAAAACAATTTCACAGCACCAAATCTCCACCTCCATCATCACCTCAACCCAAAAA

GGCATAATTAAACTTTACTTCCTCTCTTTCTTCTTCCCACTCATCCTAACCCTACTCCTAATCACATAAC

CTATTCCCCCGAGCAATCTCAATTACAATATATACACCAACAAACAATGTTCAACCAGTAACTACTACTA

ATCAACGCCCATAATCATACAAAGCCCCCGCACCAATAGGATCCTCCCGAATCAACCCTGACCCCTCTCC

TTCATAAATTATTCAGCTTCCTACACTATTAAAGTTTACCACAACCACCACCCCATCATACTCTTTCACC

CACAGCACCAATCCTACCTCCATCGCTAACCCCACTAAAACACTCACCAAGACCTCAACCCCTGACCCCC

ATGCCTCAGGATACTCCTCAATAGCCATCGCTGTAGTATATCCAAAGACAACCATCATTCCCCCTAAATA

AATTAAAAAAACTATTAAACCCATATAACCTCCCCCAAAATTCAGAATAATAACACACCCGACCACACCG

CTAACAATCAATACTAAACCCCCATAAATAGGAGAAGGCTTAGAAGAAAACCCCACAAACCCCATTACTA

AACCCACACTCAACAGAAACAAAGCATACATCATTATTCTCGCACGGACTACAACCACGACCAATGATAT

GAAAAACCATCGTTGTATTTCAACTACAAGAACACCAATGACCCCAATACGCAAAATTAACCCCCTAATA

AAATTAATTAACCACTCATTCATCGACCTCCCCACCCCATCCAACATCTCCGCATGATGAAACTTCGGCT

CACTCCTTGGCGCCTGCCTGATCCTCCAAATCACCACAGGACTATTCCTAGCCATGCACTACTCACCAGA

CGCCTCAACCGCCTTTTCATCAATCGCCCACATCACTCGAGACGTAAATTATGGCTGAATCATCCGCTAC

CTTCACGCCAATGGCGCCTCAATATTCTTTATCTGCCTCTTCCTACACATCGGGCGAGGCCTATATTACG

GATCATTTCTCTACTCAGAAACCTGAAACATCGGCATTATCCTCCTGCTTGCAACTATAGCAACAGCCTT

CATAGGCTATGTCCTCCCGTGAGGCCAAATATCATTCTGAGGGGCCACAGTAATTACAAACTTACTATCC

GCCATCCCATACATTGGGACAGACCTAGTTCAATGAATCTGAGGAGGCTACTCAGTAGACAGTCCCACCC

TCACACGATTCTTTACCTTTCACTTCATCTTGCCCTTCATTATTGCAGCCCTAGCAGCACTCCACCTCCT

ATTCTTGCACGAAACGGGATCAAACAACCCCCTAGGAATCACCTCCCATTCCGATAAAATCACCTTCCAC

CCTTACTACACAATCAAAGACGCCCTCGGCTTACTTCTCTTCCTTCTCTCCTTAATGACATTAACACTAT

TCTCACCAGACCTCCTAGGCGACCCAGACAATTATACCCTAGCCAACCCCTTAAACACCCCTCCCCACAT

CAAGCCCGAATGATATTTCCTATTCGCCTACACAATTCTCCGATCCGTCCCTAACAAACTAGGAGGCGTC

CTTGCCCTATTACTATCCATCCTCATCCTAGCAATAATCCCCATCCTCCATATATCCAAACAACAAAGCA

TAATATTTCGCCCACTAAGCCAATCACTTTATTGACTCCTAGCCGCAGACCTCCTCGTTCTAACCTGAAT

CGGAGGACAACCAGTAAGCTACCCTTTTACCATCATTGGACAAGTAGCATCCGTACTATACTTCACAACA

ATCCTAATCCTAATACCAACTATCTCCCTAATTGAAAACAAAATACTCAAATGGGCCTGTCCTTGTAGTA

TAAACTAATACACCAGTCTTGTAAACCGGAGATGAAAACCTTTTTCCAAGGACAAATCAGAGAAAAAGTC

TTTAACTCCACCATTAGCACCCAAAGCTAAGATTCTAATTTAAACTATTCTCTGTTCTTTCATGGGGAAG

CAGATTTGGGTACCACCCAAGTATTGACTCACCCATCAACAACCGCTATGTATTTCGTACATTACTGCCA

GCCACCATGAATATTGCACGGTACCATAAATACTTGACCACCTGTAGTACATAAAAACCCAATCCACATC

AACCCCCCCCCCCCATGCTTACAAGCAAGTACAGCAACCAACCCTCAACTATCACACATCAACTGCAGCT

CCAAAGCCACCTCTCACCCACTAGGATACCAACAAACCTACCCACCCTTAACAGTACATAGTACATAAAG

CCATTTACCGTACATAGCACATTACAGTCAAATCCCTTCTCGTCCCCATGGATGACCCCCCTCAGATAGG

GGTCCCTTGACCACCATCCTCCGTGAAATCAATATCCCGCACAAGAGTGCTACTCTCCTCGCTCCGGGCC

CATAACACTTGGGGGTAGCTAAAGTGAACTGTATCCGACATCTGGTTCCTACTTCAGGGCCATAAAGCCT

AAATAGCCCACACGTTCCCCTTAAATAAGACATCACGATG

>MT135514.1 Homo sapiens isolate NH35 mitochondrion, complete genome

GATCACAGGTCTATCACCCTATTAACCACTCACGGGAGCTCTCCATGCATTTGGTATTTTCGTCTGGGGG

GTGTGCACGCGATAGCATTGCGAGACGCTGGAGCCGGAGCACCCTATGTCGCAGTATCTGTCTTTGATTC

CTGCCCCATCCTATTATTTATCGCACCTACGTTCAATATTACAGGCGAACATACTTACTAAAGTGTGTTA

ATTAATTAATGCTTGTAGGACATAATAATAACAATTGAATGTCTGCACAGCCGCTTTCCACACAGACATC

ATAACAAAAAATTTCCACCAAACCCCCCCCCTCCCCCCGCTTCTGGCCACAGCACTTAAACACATCTCTG

CCAAACCCCAAAAACAAAGAACCCTAACACCAGCCTAACCAGATTTCAAATTTTATCTTTTGGCGGTATG

CACTTTTAACAGTCACCCCCCAACTAACACATTATTTTCCCCTCCCACTCCCATACTACTAATCTCATCA

ATACAACCCCCGCCCATCCTACCCAGCACACACACCGCTGCTAACCCCATACCCCGAACCAACCAAACCC

CAAAGACACCCCCCACAGTTTATGTAGCTTACCTCCTCAAAGCAATACACTGAAAATGTTTAGACGGGCT

CACATCACCCCATAAACAAATAGGTTTGGTCCTAGCCTTTCTATTAGCTCTTAGTAAGATTACACATGCA

AGCATCCCCGTTCCAGTGAGTTCACCCTCTAAATCACCACGATCAAAAGGGACAAGCATCAAGCACGCAG

CAATGCAGCTCAAAACGCTTAGCCTAGCCACACCCCCACGGGAAACAGCAGTGATTAACCTTTAGCAATA

AACGAAAGTTTAACTAAGCTATACTAACCCCAGGGTTGGTCAATTTCGTGCCAGCCACCGCGGTCACACG

ATTAACCCAAGTCAATAGAAGCCGGCGTAAAGAGTGTTTTAGATCACCCCCTCCCCAATAAAGCTAAAAC

TCACCTGAGTTGTAAAAAACTCCAGTTGACACAAAATAGACTACGAAAGTGGCTTTAACATATCTGAACA

CACAATAGCTAAGACCCAAACTGGGATTAGATACCCCACTATGCTTAGCCCTAAACCTCAACAGTTAAAT

CAACAAAACTGCTCGCCAGAACACTACGAGCCACAGCTTAAAACTCAAAGGACCTGGCGGTGCTTTATAT

CCCTCTAGAGGAGCCTGTTCTGTAATCGATAAACCCCGATCAACCTCACCACCTCTTGCTCAGCCTATAT

ACCGCCATCTTCAGCAAACCCTGATGAAGGCTACAAAGTAAGCGCAAGTACCCACGTAAAGACGTTAGGT

CAAGGTGTAGCCCATGAGGTGGCAAGAAATGGGCTACATTTTCTACCCCAGAAAACTACGATAGCCCTTA

TGAAACTTAAGGGTCGAAGGTGGATTTAGCAGTAAACTGAGAGTAGAGTGCTTAGTTGAACAGGGCCCTG

AAGCGCGTACACACCGCCCGTCACCCTCCTCAAGTATACTTCAAAGGACATTTAACTAAAACCCCTACGC

ATTTATATAGAGGAGACAAGTCGTAACATGGTAAGTGTACTGGAAAGTGCACTTGGACGAACCAGAGTGT

AGCTTAACACAAAGCACCCAACTTACACTTAGGAGATTTCAACTTAACTTGACCGCTCTGAGCTAAACCT

AGCCCCAAACCCACTCCACCTTACTACCAGACAACCTTAGCCAAACCATTTACCCAAATAAAGTATAGGC

GATAGAAATTGAAACCTGGCGCAATAGATATAGTACCGCAAGGGAAAGATGAAAAATTATAACCAAGCAT

AATATAGCAAGGACTAACCCCTATACCTTCTGCATAATGAATTAACTAGAAATAACTTTGCAAGGAGAGC

CAAAGCTAAGACCCCCGAAACCAGACGAGCTACCTAAGAACAGCTAAAAGAGCACACCCGTCTATGTAGC

AAAATAGTGGGAAGATTTATAGGTAGAGGCGACAAACCTACCGAGCCTGGTGATAGCTGGTTGTCCAAGA

TAGAATCTTAGTTCAACTTTAAATTTGCCCACAGAACCCTCTAAATCCCCTTGTAAATTTAACTGTTAGT

CCAAAGAGGAACAGCTCTTTGGACACTAGGAAAAAACCTTGTAGAGAGAGTAAAAAATTTAACACCCATA

GTAGGCCTAAAAGCAGCCACCAATTAAGAAAGCGTTCAAGCTCAACACCCACTACCTAAAAAATCCCAAA

CATATAACTGAACTCCTCACACCCAATTGGACCAATCTATCACCCTATAGAAGAACTAATGTTAGTATAA

GTAACATGAAAACATTCTCCTCCGCATAAGCCTGCGTCAGATTAAAACACTGAACTGACAATTAACAGCC

CAATATCTACAATCAACCAACAAGTCATTATTACCCTCACTGTCAACCCAACACAGGCATGCTCATAAGG

AAAGGTTAAAAAAAGTAAAAGGAACTCGGCAAATCTTACCCCGCCTGTTTACCAAAAACATCACCTCTAG

CATCACCAGTATTAGAGGCACCGCCTGCCCAGTGACACATGTTTAACGGCCGCGGTACCCTAACCGTGCA

AAGGTAGCATAATCACTTGTTCCTTAAATAGGGACCTGTATGAATGGCTCCACGAGGGTTCAGCTGTCTC

TTACTTTTAACCAGTGAAATTGACCTGCCCGTGAAGAGGCGGGCATGACACAGCAAGACGAGAAGACCCT

ATGGAGCTTTAATTTATTAATGCAAACAGTACCTAACAAACCCACAGGTCCTAAACTACCAAACCTGCAT

TAAAAATTTCGGTTGGGGCGACCTCGGAGCAGAACCCAACCTCCGAGCAGTACATGCTAAGACTTCACCA

GTCAAAGCGAACTACTATACTCAATTGATCCAATAACTTGACCAACGGAACAAGTTACCCTAGGGATAAC

AGCGCAATCCTATTCTAGAGTCCATATCAACAATAGGGTTTACGACCTCGATGTTGGATCAGGACATCCC

GATGGTGCAGCCGCTATTAAAGGTTCGTTTGTTCAACGATTAAAGTCCTACGTGATCTGAGTTCAGACCG

GAGTAATCCAGGTCGGTTTCTATCTACNTTCAAATTCCTCCCTGTACGAAAGGACAAGAGAAATAAGGCC

TACTTCACAAAGCGCCTTCCCCCGTAAATGATATCATCTCAACTTAGTATTATACCCACACCCACCCAAG

AACAGGGTTTGTTAAGATGGCAGAGCCCGGTAATCGCATAAAACTTAAAACTTTACAGTCAGAGGTTCAA

TTCCTCTTCTTAACAACATACCCATGGCCAACCTCCTACTCCTCATTGTACCCATTCTAATCGCAATGGC

ATTCCTAATGCTTACCGAACGAAAAATTCTAGGCTATATACAACTACGCAAAGGCCCCAACGTTGTAGGC

CCCTACGGGCTACTACAACCCTTCGCTGACGCCATAAAACTCTTCACCAAAGAGCCCCTAAAACCCGCCA

CATCTACCATCACCCTCTACATCACCGCCCCGACCTTAGCTCTCACCATCGCTCTTCTACTATGAACCCC

CCTCCCCATACCCAACCCCCTGGTCAACCTCAACCTAGGCCTCCTATTTATTCTAGCCACCTCTAGCCTA

GCCGTTTACTCAATCCTCTGATCAGGGTGAGCATCAAACTCAAACTACGCCCTGATCGGCGCACTGCGAG

CAGTAGCCCAAACAATCTCATATGAAGTCACCCTAGCCATCATTCTACTATCAACATTACTAATAAGTGG

CTCCTTTAACCTCTCCACCCTTATCACAACACAAGAACACCTCTGATTACTCCTGCCATCATGACCCTTG

GCCATAATATGATTTATCTCCACACTAGCAGAGACCAACCGAACCCCCTTCGACCTTGCCGAAGGGGAGT

CCGAACTAGTCTCAGGCTTCAACATCGAATACGCCGCAGGCCCCTTCGCCCTATTCTTCATAGCCGAATA

CACAAACATTATTATAATAAACACCCTCACCACTACAATCTTCCTAGGAACAACATATGACGCACTCTCC

CCTGAACTCTACACAACATATTTTGTCACCAAGACCCTACTTCTAACCTCCCTGTTCTTATGAATTCGAA

CAGCATACCCCCGATTCCGCTACGACCAACTCATACACCTCCTATGAAAAAACTTCCTACCACTCACCCT

AGCATTACTTATATGATATGTCTCCATACCCATTACAATCTCCAGCATTCCCCCTCAAACCTAAGAAATA

TGTCTGATAAAAGAGTTACTTTGATAGAGTAAATAATAGGAGCTTAAACCCCCTTATTTCTAGGACTATG

AGAATCGAACCCATCCCTGAGAATCCAAAATTCTCCGTGCCACCTATCACACCCCATCCTAAAGTAAGGT

CAGCTAAATAAGCTATCGGGCCCATACCCCGAAAATGTTGGTTATACCCTTCCCGTACTAATTAATCCCC

TGGCCCAACCCGTCATCTACTCTACCATCTTTGCAGGCACACTCATCACAGCGCTAAGCTCGCACTGATT

TTTTACCTGAGTAGGCCTAGAAATAAACATGCTAGCTTTTATTCCAGTTCTAACCAAAAAAATAAACCCT

CGTTCCACAGAAGCTGCCATCAAGTATTTCCTCACGCAAGCAACCGCATCCATAATCCTTCTAATAGCTA

TCCTCTTCAACAATATACTCTCCGGACAATGAACCATAACCAATACTACCAATCAATACTCATCATTAAT

AATCATAATAGCTATAGCAATAAAACTAGGAATAGCCCCCTTTCACTTCTGAGTCCCAGAGGTTACCCAA

GGCACCCCTCTGACATCCGGCCTGCTTCTTCTCACATGACAAAAACTAGCCCCCATCTCAATCATATACC

AAATCTCTCCCTCACTAAACGTAAGCCTTCTCCTCACTCTCTCAATCTTATCCATCATAGCAGGCAGTTG

AGGTGGATTAAACCAAACCCAGCTACGCAAAATCTTAGCATACTCCTCAATTACCCACATAGGATGAATA

ATAGCAGTTCTACCGTACAACCCTAACATAACCATTCTTAATTTAACTATTTATATTATCCTAACTACTA

CCGCATTCCTACTACTCAACTTAAACTCCAGCACCACGACCCTACTACTATCTCGCACCTGAAACAAGCT

AACATGACTAACACCCTTAATTCCATCCACCCTCCTCTCCCTAGGAGGCCTGCCCCCGCTAACCGGCTTT

TTGCCCAAATGGGCCATTATCGAAGAATTCACAAAAAACAATAGCCTCATCATCCCCACCATCATAGCCA

CCATCACCCTCCTTAACCTCTACTTCTACCTACGCCTAATCTACTCCACCTCAATCACACTACTCCCCAT

ATCTAACAACGTAAAAATAAAATGACAGTTTGAACATACAAAACCCACCCCATTCCTCCCCACACTCATC

GCCCTCACCACGCTACTCCTACCTATCTCCCCTTTTATACTAATAATCTTATAGAAATTTAGGTTAAATA

CAGACCAAGAGCCTTCAAAGCCCTCAGTAAGTTGCAATACTTAATTTCTGTAACAGCTAAGGACTGCAAA

ACCCCACTCTGCATCAACTGAACGCAAATCAGCCACTTTAATTAAGCTAAGCCCTTACTAGACCAATGGG

ACTTAAACCCACAAACACTTAGTTAACAGCTAAGCACCCTAATCAACTGGCTTCAATCTACTTCTCCCGC

CGCCGGGAAAAAAGGCGGGAGAAGCCCCGGCAGGTTTGAAGCTGCTTCTTCGAATTTGCAATTCAATATG

AAAATCACCTCGGAGCTGGTAAAAAGAGGCCTAACCCCTGTCTTTAGATTTACAGTCCAATGCTTCACTC

AGCCATTTTACCTCACCCCCACTGATGTTCGCCGACCGTTGACTATTCTCTACAAACCACAAAGACATTG

GAACACTATACCTATTATTCGGCGCATGAGCTGGAGTCCTAGGCACAGCTCTAAGCCTCCTTATTCGAGC

CGAGCTGGGCCAGCCAGGCAACCTTCTAGGTAACGACCACATCTACAACGTTATCGTCACAGCCCATGCA

TTTGTAATAATCTTCTTCATAGTAATACCCATCATAATCGGAGGCTTTGGCAACTGACTAGTTCCCCTAA

TAATCGGTGCCCCCGATATGGCGTTTCCCCGCATAAACAACATAAGCTTCTGACTCTTACCTCCCTCTCT

CCTACTCCTGCTCGCATCTGCTATAGTGGAGGCCGGAGCAGGAACAGGTTGAACAGTCTACCCTCCCTTA

GCAGGGAACTACTCCCACCCTGGAGCCTCCGTAGACCTAACCATCTTCTCCTTACACCTAGCAGGTGTCT

CCTCTATCTTAGGGGCCATCAATTTCATCACAACAATTATCAATATAAAACCCCCTGCCATAACCCAATA

CCAAACGCCCCTCTTCGTCTGATCCGTCCTAATCACAGCAGTCCTACTTCTCCTATCTCTCCCAGTCCTA

GCTGCTGGCATCACTATACTACTAACAGACCGCAACCTCAACACCACCTTCTTCGACCCCGCCGGAGGAG

GAGACCCCATTCTATACCAACACCTATTCTGATTTTTCGGTCACCCTGAAGTTTATATTCTTATCCTACC

AGGCTTCGGAATAATCTCCCATATTGTAACTTACTACTCCGGAAAAAAAGAACCATTTGGATACATAGGC

ATGGTCTGAGCTATGATATCAATTGGCTTCCTAGGGTTTATCGTGTGAGCACACCATATATTTACAGTAG

GAATAGACGTAGACACACGAGCATATTTCACCTCCGCTACCATAATCATCGCTATCCCCACCGGCGTCAA

AGTATTTAGCTGACTCGCCACACTCCACGGAAGCAATATGAAATGATCTGCTGCAGTGCTCTGAGCCCTA

GGATTCATCTTTCTTTTCACCGTAGGTGGCCTGACTGGCATTGTATTAGCAAACTCATCACTAGACATCG

TACTACACGACACGTACTACGTTGTAGCTCACTTCCACTATGTCCTATCAATAGGAGCTGTATTTGCCAT

CATAGGAGGCTTCATTCACTGATTTCCCCTATTCTCAGGCTACACCCTAGACCAAACCTACGCCAAAATC

CATTTCACTATCATATTCATCGGCGTAAATCTAACTTTCTTCCCACAACACTTTCTCGGCCTATCCGGAA

TGCCCCGACGTTACTCGGACTACCCCGATGCATACACCACATGAAACATCCTATCATCTGTAGGCTCATT

CATTTCTCTAACAGCAGTAATATTAATAATTTTCATGATTTGAGAAGCCTTCGCTTCGAAGCGAAAAGTC

CTAATAGTAGAAGAACCCTCCATAAACCTGGAGTGACTATATGGATGCCCCCCACCCTACCACACATTCG

AAGAACCCGTATACATAAAATCTAGACAAAAAAGGAAGGAATCGAACCCCCCAAAGCTGGTTTCAAGCCA

ACCCCATGGCCTCCATGACTTTTTCAAAAAGGTATTAGAAAAACCATTTCATAACTTTGTCAAAGTTAAA

TTATAGGCTAAATCCTATATATCTTAATGGCACATGCAGCGCAAGTAGGTCTACAAGACGCTACTTCCCC

TATCATAGAAGAGCTTATCACCTTTCATGATCACGCCCTCATAATCATTTTCCTTATCTGCTTCCTAGTC

CTGTATGCCCTTTTCCTAACACTCACAACAAAACTAACTAATACTAACATCTCAGACGCTCAGGAAATAG

AAACCGTCTGAACTATCCTGCCCGCCATCATCCTAGTCCTCATCGCCCTCCCATCCCTACGCATCCTTTA

CATAACAGACGAGGTCAACGATCCCTCCCTTACCATCAAATCAATTGGCCACCAATGGTACTGAACCTAC

GAGTACACCGACTACGGCGGACTAATCTTCAACTCCTACATACTTCCCCCATTATTCCTAGAACCAGGCG

ACCTGCGACTCCTTGACGTTGACAATCGAGTAGTACTCCCGATTGAAGCCCCCATTCGTATAATAATTAC

ATCACAAGACGTCTTGCACTCATGAGCTGTCCCCACATTAGGCTTAAAAACAGATGCAATTCCCGGACGT

CTAAACCAAACCACTTTCACCGCTACACGACCGGGGGTATACTACGGTCAATGCTCTGAAATCTGTGGAG

CAAACCACAGTTTCATGCCCATCGTCCTAGAATTAATTCCCCTAAAAATCTTTGAAATAGGGCCCGTATT

TACCCTATAGCACCCCCTCTAGAGCCCACTGTAAAGCTAACTTAGCATTAACCTTTTAAGTTAAAGATTA

AGAGAACCAACACCTCTTTACAGTGAAATGCCCCAACTAAATACTACCGTATGGCCCACCATAATTACCC

CCATACTCCTTACACTATTCCTCATCACCCAACTAAAAATATTAAACACAAACTACCACCTACCTCCCTC

ACCAAAGCCCATAAAAATAAAAAATTATAACAAACCCTGAGAACCAAAATGAACGAAAATCTGTTCGCTT

CATTCATTGCCCCCACAATCCTAGGCCTACCCGCCGCAGTACTGATCATTCTATTTCCCCCTCTATTGAT

CCCCACCTCCAAATATCTCATCAACAACCGACTAATCACCACCCAACAATGACTAATCAAACTAACCTCA

AAACAAATGATAACCATACACAACACTAAAGGACGAACCTGATCTCTTATACTAGTATCCTTAATCATTT

TTATTGCCACAACTAACCTCCTCGGACTCCTGCCTCACTCATTTACACCAACCACCCAACTATCTATAAA

CCTAGCCATGGCCATCCCCTTATGAGCGGGCGCAGTGATTATAGGCTTTCGCTCTAAGATTAAAAATGCC

CTAGCCCACTTCTTACCACAAGGCACACCTACACCCCTTATCCCCATACTAGTTATTATCGAAACCATCA

GCCTACTCATTCAACCAATAGCCCTGGCCGTACGCCTAACCGCTAACATTACTGCAGGCCACCTACTCAT

GCACCTAATTGGAAGCGCCACCCTAGCAATATCAACCATTAACCTTCCCTCTACACTTATCATCTTCACA

ATTCTAATTCTACTAACTATCCTAGAAATCGCTGTCGCCTTAATCCAAGCCTACGTTTTCACACTTCTAG

TAAGCCTCTACCTGCACGACAACACATAATGACCCACCAATCACATGCCTATCATATAGTAAAACCCAGC

CCATGACCCCTAACAGGGGCCCTCTCAGCCCTCCTAATGACCTCCGGCCTAGCCATGTGATTTCACTTCC

ACTCCATAACGCTCCTCATACTAGGCCTACTAACCAACACACTAACCATATACCAATGATGGCGCGATGT

AACACGAGAAAGCACATACCAAGGCCACCACACACCACCTGTCCAAAAAGGCCTTCGATACGGGATAATC

CTATTTATTACCTCAGAAGTTTTTTTCTTCGCAGGATTTTTCTGAGCCTTTTACCACTCCAGCCTAGCCC

CTACCCCCCAATTAGGAGGGCACTGGCCCCCAACAGGCATCACCCCGCTAAATCCCCTAGAAGTCCCACT

CCTAAACACATCCGTATTACTCGCATCAGGAGTATCAATCACCTGAGCTCACCATAGTCTAATAGAAAAC

AACCGAAACCAAATAATTCAAGCACTGCTTATTACAATTTTACTGGGTCTCTATTTTACCCTCCTACAAG

CCTCAGAGTACTTCGAGTCTCCCTTCACCATTTCCGACGGCATCTACGGCTCAACATTTTTTGTAGCCAC

AGGCTTCCACGGACTTCACGTCATTATTGGCTCAACTTTCCTCACTATCTGCTTCATCCGCCAACTAATA

TTTCACTTTACATCCAAACATCACTTTGGCTTCGAAGCCGCCGCCTGATACTGGCATTTTGTAGATGTGG

TTTGACTATTTCTGTATGTCTCCATCTATTGATGAGGGTCTTACTCTTTTAGTATAAATAGTACCGTTAA

CTTCCAATTAACTAGTTTTGACAACATTCAAAAAAGAGTAATAAACTTCGCCTTAATTTTAATAATCAAC

ACCCTCCTAGCCTTACTACTAATAATTATTACATTTTGACTACCACAACTCAACGGCTACATAGAAAAAT

CCACCCCTTACGAGTGCGGCTTCGACCCTATATCCCCCGCCCGCGTCCCTTTCTCCATAAAATTCTTCTT

AGTAGCTATCACCTTCTTATTATTTGATCTAGAAATTGCCCTCCTTTTACCCCTACCATGAGCCCTACAA

ACAACTAACCTGCCACTAATAGTTATGTCATCCCTCTTATTAATCATCATCCTAGCCCTAAGTCTGGCCT

ATGAGTGACTACAAAAAGGATTAGACTGAACCGAATTGGTATATAGTTTAAACAAAACGAATGATTTCGA

CTCATTAAATTATGATAATCATATTTACCAAATGCCCCTCATTTACATAAATATTATACTAGCATTTACC

ATCTCACTTCTAGGAATACTAGTATATCGCTCACACCTCATATCCTCCCTACTATGCCTAGAAGGAATAA

TACTATCGCTGTTCATTATAGCTACTCTCATAACCCTCAACACCCACTCCCTCTTAGCCAATATTGTGCC

TATTGCCATACTAGTCTTTGCCGCCTGCGAAGCAGCGGTGGGCCTAGCCCTACTAGTCTCAATCTCCAAC

ACATATGGCCTAGACTACGTACATAACCTAAACCTACTCCAATGCTAAAACTAATCGTCCCAACAATTAT

ATTACTACCACTGACATGACTTTCCAAAAAACACATAATTTGAATCAACACAACCACCCACAGCCTAATT

ATTAGCATCATCCCTCTACTATTTTTTAACCAAATCAACAACAACCTATTTAGCTGTTCCCCAACCTTTT

CCTCCGACCCCCTAACAACCCCCCTCCTAATACTAACTACCTGACTCCTACCCCTCACAATCATGGCAAG

CCAACGCCACTTATCCAGTGAACCACTATCACGAAAAAAACTCTACCTCTCTATACTAATCTCCCTACAA

ATCTCCTTAATTATAACATTCACAGCCACAGAACTAATCATATTTTATATCTTCTTCGAAACCACACTTA

TCCCCACCTTGGCTATCATCACCCGATGAGGCAACCAGCCAGAACGCCTGAACGCAGGCACATACTTCCT

ATTCTACACCCTAGTAGGCTCCCTTCCCCTACTCATCGCACTAATTTACACTCACAACACCCTAGGCTCA

CTAAACATTCTACTACTCACTCTCACTGCCCAAGAACTATCAAACTCCTGAGCCAACAACTTAATATGAC

TAGCTTACACAATAGCTTTTATAGTAAAGATACCTCTTTACGGACTCCACTTATGACTCCCTAAAGCCCA

TGTCGAAGCCCCCATCGCTGGGTCAATAGTACTTGCCGCAGTACTCTTAAAACTAGGCGGCTATGGTATA

ATACGCCTCACACTCATTCTCAACCCCCTGACAAAACACATAGCCTACCCCTTCCTTGTACTATCCCTAT

GAGGCATAATTATAACAAGCTCCATCTGCCTACGACAAACAGACCTAAAATCGCTCATTGCATACTCTTC

AATCAGCCACATAGCCCTCGTAGTAACAGCCATTCTCATCCAAACCCCCTGAAGCTTCACCGGCGCAGTC

ATTCTCATAATCGCCCACGGACTTACATCCTCATTACTATTCTGCCTAGCAAACTCAAACTACGAACGCA

CTCACAGTCGCATCATAATCCTCTCTCAAGGACTTCAAACTCTACTCCCACTAATAGCTTTTTGATGACT

TCTAGCAAGCCTCGCTAACCTCGCCTTACCCCCCACTATTAACCTACTGGGAGAACTCTCTGTGCTAGTA

ACCACGTTCTCCTGATCAAATATCACTCTCCTACTTACAGGACTCAACATACTAGTCACAGCCCTATACT

CCCTCTACATATTTACCACAACACAATGGGGCTCACTCACCCACCACATTAACAACATAAAACCCTCATT

CACACGAGAAAACACCCTCATGTTCATACACCTATCCCCCATTCTCCTCCTATCCCTCAACCCCGACATC

ATTACCGGGTTTTCCTCTTGTAAATATAGTTTAACCAAAACATCAGATTGTGAATCTGACAACAGAGGCT

TACGACCCCTTATTTACCGAGAAAGCTCACAAGAACTGCTAACTCATGCCTCCATGTCTAACAACATGGC

TTTCTCAACTTTTAAAGGATAACAGCTATCCATTGGTCTTAGGCCCCAAAAATTTTGGTGCAACTCCAAA

TAAAAGTAATAACCATGCACACTACTATAACCACCCTAACCCTGACTTCCCTAATTCCCCCCATCCTTAC

CACCCTCGTTAACCCTAACAAAAAAAACTCATACCCCCATTATGTAAAATCCATTGTCGCATCCACCTTT

ATTATCAGTCTCTTCCCCACAACAATATTCATGTGCCTAGACCAAGAAGTTATTATCTCGAACTGACACT

GAGCCACAACCCAAACAACCCAGCTCTCCCTAAGCTTCAAACTAGACTACTTCTCCATAATATTCATCCC

TGTAGCATTGTTCGTTACATGGTCCATCATAGAATTCTCACTGTGATATATAAACTCAGACCCAAACATT

AATCAGTTCTTCAAATATCTACTCATCTTCCTAATTACCATACTAATCTTAGTTACCGCTAACAACCTAT

TCCAACTGTTCATCGGCTGAGAGGGCGTAGGAATTATATCCTTCTTGCTCATCAGTTGATGATACGCCCG

AGCAGATGCCAACACAGCAGCCATTCAAGCAATCCTATACAACCGTATCGGCGATATCGGTTTCATCCTC

GCCTTAGCATGATTTATCCTACACTCCAACTCATGAGACCCACAACAAATAGCCCTTCTAAACGCTAATC

CAAGCCTCACCCCACTACTAGGCCTCCTCCTAGCAGCAGCAGGCAAATCAGCCCAATTAGGTCTCCACCC

CTGACTCCCCTCAGCCATAGAAGGCCCCACCCCAGTCTCAGCCCTACTCCACTCAAGCACTATAGTTGTA

GCAGGAATCTTCTTACTCATCCGCTTCCACCCCCTAGCAGAAAATAGCCCACTAATCCAAACTCTAACAC

TATGCTTAGGCGCTATCACCACTCTGTTCGCAGCAGTCTGCGCCCTTACACAAAATGACATCAAAAAAAT

CGTAGCCTTCTCCACTTCAAGTCAACTAGGACTCATAATAGTTACAATCGGCATCAACCAACCACACCTA

GCATTCCTGCACATCTGTACCCACGCCTTCTTCAAAGCCATACTATTTATGTGCTCCGGGTCCATCATCC

ACAACCTTAACAATGAACAAGATATTCGAAAAATAGGAGGACTACTCAAAACCATACCTCTCACTTCAAC

CTCCCTCACCATTGGCAGCCTAGCATTAGCAGGAATACCTTTCCTCACAGGTTTCTACTCCAAAGACCAC

ATCATCGAAACCGCAAACATATCATACACAAACGCCTGAGCCCTATCTATTACTCTCATCGCTACCTCCC

TGACAAGCGCCTATAGCACTCGAATAATTCTTCTCACCCTAACAGGTCAACCTCGCTTCCCCACCCTTAC

TAACATTAACGAAAATAACCCCACCCTACTAAACCCCATTAAACGCCTGGCAGCCGGAAGCCTATTCGCA

GGATTTCTCATTACTAACAACATTTCCCCCGCATCCCCCTTCCAAACAACAATCCCCCTCTACCTAAAAC

TCACAGCCCTCGCTGTCACTTTCCTAGGACTTCTAACAGCCCTAGACCTCAACTACCTAACCAACAAACT

TAAAATAAAATCCCCACTATGCACATTTTATTTCTCCAACATACTCGGATTCTACCCTAGCATCACACAC

CGCACAATCCCCTATCTAGGCCTTCTTACGAGCCAAAACCTGCCCCTACTCCTCCTAGACCTAACCTGAC

TAGAAAAGCTATTGCCTAAAACAATTTCACAGCACCAAATCTCCACCTCCATCATCACCTCAACCCAAAA

AGGCATAATTAAACTTTACTTCCTCTCTTTCTTCTTCCCACTCATCCTAACCCTACTCCTAATCACATAA

CCTATTCCCCCGAGCAATCTCAATTACAATATATACACCAACAAACAATGTTCAACCAGTAACTACTACT

AATCAACGCCCATAATCATACAAAGCCCCCGCACCAATAGGATCCTCCCGAATCAACCCTGACCCCTCTC

CTTCATAAATTATTCAGCTTCCTACACTATTAAAGTTTACCACAACCACCACCCCATCATACTCTTTCAC

CCACAGCACCAATCCTACCTCCATCGCTAACCCCACTAAAACACTCACCAAGACCTCAACCCCTGACCCC

CATGCCTCAGGATACTCCTCAATAGCCATCGCTGTAGTATATCCAAAGACAACCATCATTCCCCCTAAAT

AAATTAAAAAAACTATTAAACCCATATAACCTCCCCCAAAATTCAGAATAATAACACACCCGACCACACC

GCTAACAATCAATACTAAACCCCCATAAATAGGAGAAGGCTTAGAAGAAAACCCCACAAACCCCATTACT

AAACCCACACTCAACAGAAACAAAGCATACATCATTATTCTCGCACGGACTACAACCACGACCAATGATA

TGAAAAACCATCGTTGTATTTCAACTACAAGAACACCAATGACCCCAATACGCAAAATTAACCCCCTAAT

AAAATTAATTAACCACTCATTCATCGACCTCCCCACCCCATCCAACATCTCCGCATGATGAAACTTCGGC

TCACTCCTTGGCGCCTGCCTGATCCTCCAAATCACCACAGGACTATTCCTAGCCATGCACTACTCACCAG

ACGCCTCAACCGCCTTTTCATCAATCGCCCACATCACTCGAGACGTAAATTATGGCTGAATCATCCGCTA

CCTTCACGCCAATGGCGCCTCAATATTCTTTATCTGCCTCTTCCTACACATCGGGCGAGGCCTATATTAC

GGATCATTTCTCTACTCAGAAACCTGAAACATCGGCATTATCCTCCTGCTTGCAACTATAGCAACAGCCT

TCATAGGCTATGTCCTCCCGTGAGGCCAAATATCATTCTGAGGGGCCACAGTAATTACAAACTTACTATC

CGCCATCCCATACATTGGGACAGACCTAGTTCAATGAATCTGAGGAGGCTACTCAGTAGACAGTCCCACC

CTCACACGATTCTTTACCTTTCACTTCATCTTGCCCTTCATTATTGCAGCCCTAGCAGCACTCCACCTCC

TATTCTTGCACGAAACGGGATCAAACAACCCCCTAGGAATCACCTCCCATTCCGATAAAATCACCTTCCA

CCCTTACTACACAATCAAAGACGCCCTCGGCTTACTTCTCTTCCTTCTCTCCTTAATGACATTAACACTA

TTCTCACCAGACCTCCTAGGCGACCCAGACAATTATACCCTAGCCAACCCCTTAAACACCCCTCCCCACA

TCAAGCCCGAATGATATTTCCTATTCGCCTACACAATTCTCCGATCCGTCCCTAACAAACTAGGAGGCGT

CCTTGCCCTATTACTATCCATCCTCATCCTAGCAATAATCCCCATCCTCCATATATCCAAACAACAAAGC

ATAATATTTCGCCCACTAAGCCAATCACTTTATTGACTCCTAGCCGCAGACCTCCTCGTTCTAACCTGAA

TCGGAGGACAACCAGTAAGCTACCCTTTTACCATCATTGGACAAGTAGCATCCGTACTATACTTCACAAC

AATCCTAATCCTAATACCAACTATCTCCCTAATTGAAAACAAAATACTCAAATGGGCCTGTCCTTGTAGT

ATAAACTAATACACCAGTCTTGTAAACCGGAGATGAAAACCTTTTTCCAAGGACAAATCAGAGAAAAAGT

CTTTAACTCCACCATTAGCACCCAAAGCTAAGATTCTAATTTAAACTATTCTCTGTTCTTTCATGGGGAA

GCAGATTTGGGTACCACCCAAGTATTGACTCACCCATCAACAACCGCTATGTATTTCGTACATTACTGCC

AGCCACCATGAATATTGTACGGTACCATAAATACTTGACCACCTGTAGTACATAAAAACCCAATCCACAT

CAACCCCCCCCCCCCATGCTTACAAGCAAGTACAGCAACCAACCCTCAACTATCACACATCAACTGCAGC

TCCAAAGCCACCTCTCACCCACTAGGATACCAACAAACCTACCCACCCTTAACAGTACATAGTACATAAA

GCCATTTACCGTACATAGCACATTACAGTCAAATCCCTTCTCGTCCCCATGGATGACCCCCCTCAGATAG

GGGTCCCTTGACCACCATCCTCCGTGAAATCAATATCCCGCACAAGAGTGCTACTCTCCTCGCTCCGGGC

CCATAACACTTGGGGGTAGCTAAAGTGAACTGTATCCGACATCTGGTTCCTACTTCAGGGCCATAAAGCC

TAAATAGCCCACACGTTCCCCTTAAATAAGACATCACGATG

>MT135515.1 Homo sapiens isolate TA2 mitochondrion, complete genome

GATCACAGGTCTATCACCCTATTAACCACTCACGGGAGCTCTCCATGCATTTGGTATTTTCGTCTGGGGG

GTGTGCACGCGATAGCATTGCGAGACGCTGGAGCCGGAGCACCCTATGTCGCAGTATCTGTCTTTGATTC

CTGCCCCATCCTATTATTTATCGCACCTACGTTCAATATTACAGGCGAACATACTTACTAAAGTGTGTTA

ATTAATTAATGCTTGTAGGACATAATAATAACAATTGAATGTCTGCACAGCCGCTTTCCACACAGACATC

ATAACAAAAAATTTCCACCAAACCCCCCCCCTCCCCCCGCTTCTGGCCACAGCACTTAAACACATCTCTG

CCAAACCCCAAAAACAAAGAACCCTAACACCAGCCTAACCAGATTTCAAATTTTATCTTTTGGCGGTATG

CACTTTTAACAGTCACCCCCCAACTAACACATTATTTTCCCCTCCCACTCCCATACTACTAATCTCATCA

ATACAACCCCCGCCCATCCTACCCAGCACACACACCGCTGCTAACCCCATACCCCGAACCAACCAAACCC

CAAAGACACCCCCCACAGTTTATGTAGCTTACCTCCTCAAAGCAATACACTGAAAATGTTTAGACGGGCT

CACATCACCCCATAAACAAATAGGTTTGGTCCTAGCCTTTCTATTAGCTCTTAGTAAGATTACACATGCA

AGCATCCCCGTTCCAGTGAGTTCACCCTCTAAATCACCACGATCAAAAGGGACAAGCATCAAGCACGCAG

CAATGCAGCTCAAAACGCTTAGCCTAGCCACACCCCCACGGGAAACAGCAGTGATTAACCTTTAGCAATA

AACGAAAGTTTAACTAAGCTATACTAACCCCAGGGTTGGTCAATTTCGTGCCAGCCACCGCGGTCACACG

ATTAACCCAAGTCAATAGAAGCCGGCGTAAAGAGTGTTTTAGATCACCCCCTCCCCAATAAAGCTAAAAC

TCACCTGAGTTGTAAAAAACTCCAGTTGACACAAAATAGACTACGAAAGTGGCTTTAACATATCTGAACA

CACAATAGCTAAGACCCAAACTGGGATTAGATACCCCACTATGCTTAGCCCTAAACCTCAACAGTTAAAT

CAACAAAACTGCTCGCCAGAACACTACGAGCCACAGCTTAAAACTCAAAGGACCTGGCGGTGCTTTATAT

CCCTCTAGAGGAGCCTGTTCTGTAATCGATAAACCCCGATCAACCTCACCACCTCTTGCTCAGCCTATAT

ACCGCCATCTTCAGCAAACCCTGATGAAGGCTACAAAGTAAGCGCAAGTACCCACGTAAAGACGTTAGGT

CAAGGTGTAGCCCATGAGGTGGCAAGAAATGGGCTACATTTTCTACCCCAGAAAACTACGATAGCCCTTA

TGAAACTTAAGGGTCGAAGGTGGATTTAGCAGTAAACTGAGAGTAGAGTGCTTAGTTGAACAGGGCCCTG

AAGCGCGTACACACCGCCCGTCACCCTCCTCAAGTATACTTCAAAGGACATTTAACTAAAACCCCTACGC

ATTTATATAGAGGAGACAAGTCGTAACATGGTAAGTGTACTGGAAAGTGCACTTGGACGAACCAGAGTGT

AGCTTAACACAAAGCACCCAACTTACACTTAGGAGATTTCAACTTAACTTGACCGCTCTGAGCTAAACCT

AGCCCCAAACCCACTCCACCTTACTACCAGACAACCTTAGCCAAACCATTTACCCAAATAAAGTATAGGC

GATAGAAATTGAAACCTGGCGCAATAGATATAGTACCGCAAGGGAAAGATGAAAAATTATAACCAAGCAT

AATATAGCAAGGACTAACCCCTATACCTTCTGCATAATGAATTAACTAGAAATAACTTTGCAAGGAGAGC

CAAAGCTAAGACCCCCGAAACCAGACGAGCTACCTAAGAACAGCTAAAAGAGCACACCCGTCTATGTAGC

AAAATAGTGGGAAGATTTATAGGTAGAGGCGACAAACCTACCGAGCCTGGTGATAGCTGGTTGTCCAAGA

TAGAATCTTAGTTCAACTTTAAATTTGCCCACAGAACCCTCTAAATCCCCTTGTAAATTTAACTGTTAGT

CCAAAGAGGAACAGCTCTTTGGACACTAGGAAAAAACCTTGTAGAGAGAGTAAAAAATTTAACACCCATA

GTAGGCCTAAAAGCAGCCACCAATTAAGAAAGCGTTCAAGCTCAACACCCACTACCTAAAAAATCCCAAA

CATATAACTGAACTCCTCACACCCAATTGGACCAATCTATCACCCTATAGAAGAACTAATGTTAGTATAA

GTAACATGAAAACATTCTCCTCCGCATAAGCCTGCGTCAGATTAAAACACTGAACTGACAATTAACAGCC

CAATATCTACAATCAACCAACAAGTCATTATTACCCTCACTGTCAACCCAACACAGGCATGCTCATAAGG

AAAGGTTAAAAAAAGTAAAAGGAACTCGGCAAATCTTACCCCGCCTGTTTACCAAAAACATCACCTCTAG

CATCACCAGTATTAGAGGCACCGCCTGCCCAGTGACACATGTTTAACGGCCGCGGTACCCTAACCGTGCA

AAGGTAGCATAATCACTTGTTCCTTAAATAGGGACCTGTATGAATGGCTCCACGAGGGTTCAGCTGTCTC

TTACTTTTAACCAGTGAAATTGACCTGCCCGTGAAGAGGCGGGCATGACACAGCAAGACGAGAAGACCCT

ATGGAGCTTTAATTTATTAATGCAAACAGTACCTAACAAACCCACAGGTCCTAAACTACCAAACCTGCAT

TAAAAATTTCGGTTGGGGCGACCTCGGAGCAGAACCCAACCTCCGAGCAGTACATGCTAAGACTTCACCA

GTCAAAGCGAACTACTATACTCAATTGATCCAATAACTTGACCAACGGAACAAGTTACCCTAGGGATAAC

AGCGCAATCCTATTCTAGAGTCCATATCAACAATAGGGTTTACGACCTCGATGTTGGATCAGGACATCCC

GATGGTGCAGCCGCTATTAAAGGTTCGTTTGTTCAACGATTAAAGTCCTACGTGATCTGAGTTCAGACCG

GAGTAATCCAGGTCGGTTTCTATCTACNTTCAAATTCCTCCCTGTACGAAAGGACAAGAGAAATAAGGCC

TACTTCACAAAGCGCCTTCCCCCGTAAATGATATCATCTCAACTTAGTATTATACCCACACCCACCCAAG

AACAGGGTTTGTTAAGATGGCAGAGCCCGGTAATCGCATAAAACTTAAAACTTTACAGTCAGAGGTTCAA

TTCCTCTTCTTAACAACATACCCATGGCCAACCTCCTACTCCTCATTGTACCCATTCTAATCGCAATGGC

ATTCCTAATGCTTACCGAACGAAAAATTCTAGGCTATATACAACTACGCAAAGGCCCCAACGTTGTAGGC

CCCTACGGGCTACTACAACCCTTCGCTGACGCCATAAAACTCTTCACCAAAGAGCCCCTAAAACCCGCCA

CATCTACCATCACCCTCTACATCACCGCCCCGACCTTAGCTCTCACCATCGCTCTTCTACTATGAACCCC

CCTCCCCATACCCAACCCCCTGGTCAACCTCAACCTAGGCCTCCTATTTATTCTAGCCACCTCTAGCCTA

GCCGTTTACTCAATCCTCTGATCAGGGTGAGCATCAAACTCAAACTACGCCCTGATCGGCGCACTGCGAG

CAGTAGCCCAAACAATCTCATATGAAGTCACCCTAGCCATCATTCTACTATCAACATTACTAATAAGTGG

CTCCTTTAACCTCTCCACCCTTATCACAACACAAGAACACCTCTGATTACTCCTGCCATCATGACCCTTG

GCCATAATATGATTTATCTCCACACTAGCAGAGACCAACCGAACCCCCTTCGACCTTGCCGAAGGGGAGT

CCGAACTAGTCTCAGGCTTCAACATCGAATACGCCGCAGGCCCCTTCGCCCTATTCTTCATAGCCGAATA

CACAAACATTATTATAATAAACACCCTCACCACTACAATCTTCCTAGGAACAACATATGACGCACTCTCC

CCTGAACTCTACACAACATATTTTGTCACCAAGACCCTACTTCTAACCTCCCTGTTCTTATGAATTCGAA

CAGCATACCCCCGATTCCGCTACGACCAACTCATACACCTCCTATGAAAAAACTTCCTACCACTCACCCT

AGCATTACTTATATGATATGTCTCCATACCCATTACAATCTCCAGCATTCCCCCTCAAACCTAAGAAATA

TGTCTGATAAAAGAGTTACTTTGATAGAGTAAATAATAGGAGCTTAAACCCCCTTATTTCTAGGACTATG

AGAATCGAACCCATCCCTGAGAATCCAAAATTCTCCGTGCCACCTATCACACCCCATCCTAAAGTAAGGT

CAGCTAAATAAGCTATCGGGCCCATACCCCGAAAATGTTGGTTATACCCTTCCCGTACTAATTAATCCCC

TGGCCCAACCCGTCATCTACTCTACCATCTTTGCAGGCACACTCATCACAGCGCTAAGCTCGCACTGATT

TTTTACCTGAGTAGGCCTAGAAATAAACATGCTAGCTTTTATTCCAGTTCTAACCAAAAAAATAAACCCT

CGTTCCACAGAAGCTGCCATCAAGTATTTCCTCACGCAAGCAACCGCATCCATAATCCTTCTAATAGCTA

TCCTCTTCAACAATATACTCTCCGGACAATGAACCATAACCAATACTACCAATCAATACTCATCATTAAT

AATCATAATAGCTATAGCAATAAAACTAGGAATAGCCCCCTTTCACTTCTGAGTCCCAGAGGTTACCCAA

GGCACCCCTCTGACATCCGGCCTGCTTCTTCTCACATGACAAAAACTAGCCCCCATCTCAATCATATACC

AAATCTCTCCCTCACTAAACGTAAGCCTTCTCCTCACTCTCTCAATCTTATCCATCATAGCAGGCAGTTG

AGGTGGATTAAACCAAACCCAGCTACGCAAAATCTTAGCATACTCCTCAATTACCCACATAGGATGAATA

ATAGCAGTTCTACCGTACAACCCTAACATAACCATTCTTAATTTAACTATTTATATTATCCTAACTACTA

CCGCATTCCTACTACTCAACTTAAACTCCAGCACCACGACCCTACTACTATCTCGCACCTGAAACAAGCT

AACATGACTAACACCCTTAATTCCATCCACCCTCCTCTCCCTAGGAGGCCTGCCCCCGCTAACCGGCTTT

TTGCCCAAATGGGCCATTATCGAAGAATTCACAAAAAACAATAGCCTCATCATCCCCACCATCATAGCCA

CCATCACCCTCCTTAACCTCTACTTCTACCTACGCCTAATCTACTCCACCTCAATCACACTACTCCCCAT

ATCTAACAACGTAAAAATAAAATGACAGTTTGAACATACAAAACCCACCCCATTCCTCCCCACACTCATC

GCCCTCACCACGCTACTCCTACCTATCTCCCCTTTTATACTAATAATCTTATAGAAATTTAGGTTAAATA

CAGACCAAGAGCCTTCAAAGCCCTCAGTAAGTTGCAATACTTAATTTCTGTAACAGCTAAGGACTGCAAA

ACCCCACTCTGCATCAACTGAACGCAAATCAGCCACTTTAATTAAGCTAAGCCCTTACTAGACCAATGGG

ACTTAAACCCACAAACACTTAGTTAACAGCTAAGCACCCTAATCAACTGGCTTCAATCTACTTCTCCCGC

CGCCGGGAAAAAAGGCGGGAGAAGCCCCGGCAGGTTTGAAGCTGCTTCTTCGAATTTGCAATTCAATATG

AAAATCACCTCGGAGCTGGTAAAAAGAGGCCTAACCCCTGTCTTTAGATTTACAGTCCAATGCTTCACTC

AGCCATTTTACCTCACCCCCACTGATGTTCGCCGACCGTTGACTATTCTCTACAAACCACAAAGACATTG

GAACACTATACCTATTATTCGGCGCATGAGCTGGAGTCCTAGGCACAGCTCTAAGCCTCCTTATTCGAGC

CGAGCTGGGCCAGCCAGGCAACCTTCTAGGTAACGACCACATCTACAACGTTATCGTCACAGCCCATGCA

TTTGTAATAATCTTCTTCATAGTAATACCCATCATAATCGGAGGCTTTGGCAACTGACTAGTTCCCCTAA

TAATCGGTGCCCCCGATATGGCGTTTCCCCGCATAAACAACATAAGCTTCTGACTCTTACCTCCCTCTCT

CCTACTCCTGCTCGCATCTGCTATAGTGGAGGCCGGAGCAGGAACAGGTTGAACAGTCTACCCTCCCTTA

GCAGGGAACTACTCCCACCCTGGAGCCTCCGTAGACCTAACCATCTTCTCCTTACACCTAGCAGGTGTCT

CCTCTATCTTAGGGGCCATCAATTTCATCACAACAATTATCAATATAAAACCCCCTGCCATAACCCAATA

CCAAACGCCCCTCTTCGTCTGATCCGTCCTAATCACAGCAGTCCTACTTCTCCTATCTCTCCCAGTCCTA

GCTGCTGGCATCACTATACTACTAACAGACCGCAACCTCAACACCACCTTCTTCGACCCCGCCGGAGGAG

GAGACCCCATTCTATACCAACACCTATTCTGATTTTTCGGTCACCCTGAAGTTTATATTCTTATCCTACC

AGGCTTCGGAATAATCTCCCATATTGTAACTTACTACTCCGGAAAAAAAGAACCATTTGGATACATAGGC

ATGGTCTGAGCTATGATATCAATTGGCTTCCTAGGGTTTATCGTGTGAGCACACCATATATTTACAGTAG

GAATAGACGTAGACACACGAGCATATTTCACCTCCGCTACCATAATCATCGCTATCCCCACCGGCGTCAA

AGTATTTAGCTGACTCGCCACACTCCACGGAAGCAATATGAAATGATCTGCTGCAGTGCTCTGAGCCCTA

GGATTCATCTTTCTTTTCACCGTAGGTGGCCTGACTGGCATTGTATTAGCAAACTCATCACTAGACATCG

TACTACACGACACGTACTACGTTGTAGCTCACTTCCACTATGTCCTATCAATAGGAGCTGTATTTGCCAT

CATAGGAGGCTTCATTCACTGATTTCCCCTATTCTCAGGCTACACCCTAGACCAAACCTACGCCAAAATC

CATTTCACTATCATATTCATCGGCGTAAATCTAACTTTCTTCCCACAACACTTTCTCGGCCTATCCGGAA

TGCCCCGACGTTACTCGGACTACCCCGATGCATACACCACATGAAACATCCTATCATCTGTAGGCTCATT

CATTTCTCTAACAGCAGTAATATTAATAATTTTCATGATTTGAGAAGCCTTCGCTTCGAAGCGAAAAGTC

CTAATAGTAGAAGAACCCTCCATAAACCTGGAGTGACTATATGGATGCCCCCCACCCTACCACACATTCG

AAGAACCCGTATACATAAAATCTAGACAAAAAAGGAAGGAATCGAACCCCCCAAAGCTGGTTTCAAGCCA

ACCCCATGGCCTCCATGACTTTTTCAAAAAGGTATTAGAAAAACCATTTCATAACTTTGTCAAAGTTAAA

TTATAGGCTAAATCCTATATATCTTAATGGCACATGCAGCGCAAGTAGGTCTACAAGACGCTACTTCCCC

TATCATAGAAGAGCTTATCACCTTTCATGATCACGCCCTCATAATCATTTTCCTTATCTGCTTCCTAGTC

CTGTATGCCCTTTTCCTAACACTCACAACAAAACTAACTAATACTAACATCTCAGACGCTCAGGAAATAG

AAACCGTCTGAACTATCCTGCCCGCCATCATCCTAGTCCTCATCGCCCTCCCATCCCTACGCATCCTTTA

CATAACAGACGAGGTCAACGATCCCTCCCTTACCATCAAATCAATTGGCCACCAATGGTACTGAACCTAC

GAGTACACCGACTACGGCGGACTAATCTTCAACTCCTACATACTTCCCCCATTATTCCTAGAACCAGGCG

ACCTGCGACTCCTTGACGTTGACAATCGAGTAGTACTCCCGATTGAAGCCCCCATTCGTATAATAATTAC

ATCACAAGACGTCTTGCACTCATGAGCTGTCCCCACATTAGGCTTAAAAACAGATGCAATTCCCGGACGT

CTAAACCAAACCACTTTCACCGCTACACGACCGGGGGTATACTACGGTCAATGCTCTGAAATCTGTGGAG

CAAACCACAGTTTCATGCCCATCGTCCTAGAATTAATTCCCCTAAAAATCTTTGAAATAGGGCCCGTATT

TACCCTATAGCACCCCCTCTAGAGCCCACTGTAAAGCTAACTTAGCATTAACCTTTTAAGTTAAAGATTA

AGAGAACCAACACCTCTTTACAGTGAAATGCCCCAACTAAATACTACCGTATGGCCCACCATAATTACCC

CCATACTCCTTACACTATTCCTCATCACCCAACTAAAAATATTAAACACAAACTACCACCTACCTCCCTC

ACCAAAGCCCATAAAAATAAAAAATTATAACAAACCCTGAGAACCAAAATGAACGAAAATCTGTTCGCTT

CATTCATTGCCCCCACAATCCTAGGCCTACCCGCCGCAGTACTGATCATTCTATTTCCCCCTCTATTGAT

CCCCACCTCCAAATATCTCATCAACAACCGACTAATCACCACCCAACAATGACTAATCAAACTAACCTCA

AAACAAATGATAACCATACACAACACTAAAGGACGAACCTGATCTCTTATACTAGTATCCTTAATCATTT

TTATTGCCACAACTAACCTCCTCGGACTCCTGCCTCACTCATTTACACCAACCACCCAACTATCTATAAA

CCTAGCCATGGCCATCCCCTTATGAGCGGGCGCAGTGATTATAGGCTTTCGCTCTAAGATTAAAAATGCC

CTAGCCCACTTCTTACCACAAGGCACACCTACACCCCTTATCCCCATACTAGTTATTATCGAAACCATCA

GCCTACTCATTCAACCAATAGCCCTGGCCGTACGCCTAACCGCTAACATTACTGCAGGCCACCTACTCAT

GCACCTAATTGGAAGCGCCACCCTAGCAATATCAACCATTAACCTTCCCTCTACACTTATCATCTTCACA

ATTCTAATTCTACTAACTATCCTAGAAATCGCTGTCGCCTTAATCCAAGCCTACGTTTTCACACTTCTAG

TAAGCCTCTACCTGCACGACAACACATAATGACCCACCAATCACATGCCTATCATATAGTAAAACCCAGC

CCATGACCCCTAACAGGGGCCCTCTCAGCCCTCCTAATGACCTCCGGCCTAGCCATGTGATTTCACTTCC

ACTCCATAACGCTCCTCATACTAGGCCTACTAACCAACACACTAACCATATACCAATGATGGCGCGATGT

AACACGAGAAAGCACATACCAAGGCCACCACACACCACCTGTCCAAAAAGGCCTTCGATACGGGATAATC

CTATTTATTACCTCAGAAGTTTTTTTCTTCGCAGGATTTTTCTGAGCCTTTTACCACTCCAGCCTAGCCC

CTACCCCCCAATTAGGAGGGCACTGGCCCCCAACAGGCATCACCCCGCTAAATCCCCTAGAAGTCCCACT

CCTAAACACATCCGTATTACTCGCATCAGGAGTATCAATCACCTGAGCTCACCATAGTCTAATAGAAAAC

AACCGAAACCAAATAATTCAAGCACTGCTTATTACAATTTTACTGGGTCTCTATTTTACCCTCCTACAAG

CCTCAGAGTACTTCGAGTCTCCCTTCACCATTTCCGACGGCATCTACGGCTCAACATTTTTTGTAGCCAC

AGGCTTCCACGGACTTCACGTCATTATTGGCTCAACTTTCCTCACTATCTGCTTCATCCGCCAACTAATA

TTTCACTTTACATCCAAACATCACTTTGGCTTCGAAGCCGCCGCCTGATACTGGCATTTTGTAGATGTGG

TTTGACTATTTCTGTATGTCTCCATCTATTGATGAGGGTCTTACTCTTTTAGTATAAATAGTACCGTTAA

CTTCCAATTAACTAGTTTTGACAACATTCAAAAAAGAGTAATAAACTTCGCCTTAATTTTAATAATCAAC

ACCCTCCTAGCCTTACTACTAATAATTATTACATTTTGACTACCACAACTCAACGGCTACATAGAAAAAT

CCACCCCTTACGAGTGCGGCTTCGACCCTATATCCCCCGCCCGCGTCCCTTTCTCCATAAAATTCTTCTT

AGTAGCTATCACCTTCTTATTATTTGATCTAGAAATTGCCCTCCTTTTACCCCTACCATGAGCCCTACAA

ACAACTAACCTGCCACTAATAGTTATGTCATCCCTCTTATTAATCATCATCCTAGCCCTAAGTCTGGCCT

ATGAGTGACTACAAAAAGGATTAGACTGAACCGAATTGGTATATAGTTTAAACAAAACGAATGATTTCGA

CTCATTAAATTATGATAATCATATTTACCAAATGCCCCTCATTTACATAAATATTATACTAGCATTTACC

ATCTCACTTCTAGGAATACTAGTATATCGCTCACACCTCATATCCTCCCTACTATGCCTAGAAGGAATAA

TACTATCGCTGTTCATTATAGCTACTCTCATAACCCTCAACACCCACTCCCTCTTAGCCAATATTGTGCC

TATTGCCATACTAGTCTTTGCCGCCTGCGAAGCAGCGGTGGGCCTAGCCCTACTAGTCTCAATCTCCAAC

ACATATGGCCTAGACTACGTACATAACCTAAACCTACTCCAATGCTAAAACTAATCGTCCCAACAATTAT

ATTACTACCACTGACATGACTTTCCAAAAAACACATAATTTGAATCAACACAACCACCCACAGCCTAATT

ATTAGCATCATCCCTCTACTATTTTTTAACCAAATCAACAACAACCTATTTAGCTGTTCCCCAACCTTTT

CCTCCGACCCCCTAACAACCCCCCTCCTAATACTAACTACCTGACTCCTACCCCTCACAATCATGGCAAG

CCAACGCCACTTATCCAGTGAACCACTATCACGAAAAAAACTCTACCTCTCTATACTAATCTCCCTACAA

ATCTCCTTAATTATAACATTCACAGCCACAGAACTAATCATATTTTATATCTTCTTCGAAACCACACTTA

TCCCCACCTTGGCTATCATCACCCGATGAGGCAACCAGCCAGAACGCCTGAACGCAGGCACATACTTCCT

ATTCTACACCCTAGTAGGCTCCCTTCCCCTACTCATCGCACTAATTTACACTCACAACACCCTAGGCTCA

CTAAACATTCTACTACTCACTCTCACTGCCCAAGAACTATCAAACTCCTGAGCCAACAACTTAATATGAC

TAGCTTACACAATAGCTTTTATAGTAAAGATACCTCTTTACGGACTCCACTTATGACTCCCTAAAGCCCA

TGTCGAAGCCCCCATCGCTGGGTCAATAGTACTTGCCGCAGTACTCTTAAAACTAGGCGGCTATGGTATA

ATACGCCTCACACTCATTCTCAACCCCCTGACAAAACACATAGCCTACCCCTTCCTTGTACTATCCCTAT

GAGGCATAATTATAACAAGCTCCATCTGCCTACGACAAACAGACCTAAAATCGCTCATTGCATACTCTTC

AATCAGCCACATAGCCCTCGTAGTAACAGCCATTCTCATCCAAACCCCCTGAAGCTTCACCGGCGCAGTC

ATTCTCATAATCGCCCACGGACTTACATCCTCATTACTATTCTGCCTAGCAAACTCAAACTACGAACGCA

CTCACAGTCGCATCATAATCCTCTCTCAAGGACTTCAAACTCTACTCCCACTAATAGCTTTTTGATGACT

TCTAGCAAGCCTCGCTAACCTCGCCTTACCCCCCACTATTAACCTACTGGGAGAACTCTCTGTGCTAGTA

ACCACGTTCTCCTGATCAAATATCACTCTCCTACTTACAGGACTCAACATACTAGTCACAGCCCTATACT

CCCTCTACATATTTACCACAACACAATGGGGCTCACTCACCCACCACATTAACAACATAAAACCCTCATT

CACACGAGAAAACACCCTCATGTTCATACACCTATCCCCCATTCTCCTCCTATCCCTCAACCCCGACATC

ATTACCGGGTTTTCCTCTTGTAAATATAGTTTAACCAAAACATCAGATTGTGAATCTGACAACAGAGGCT

TACGACCCCTTATTTACCGAGAAAGCTCACAAGAACTGCTAACTCATGCCTCCATGTCTAACAACATGGC

TTTCTCAACTTTTAAAGGATAACAGCTATCCATTGGTCTTAGGCCCCAAAAATTTTGGTGCAACTCCAAA

TAAAAGTAATAACCATGCACACTACTATAACCACCCTAACCCTGACTTCCCTAATTCCCCCCATCCTTAC

CACCCTCGTTAACCCTAACAAAAAAAACTCATACCCCCATTATGTAAAATCCATTGTCGCATCCACCTTT

ATTATCAGTCTCTTCCCCACAACAATATTCATGTGCCTAGACCAAGAAGTTATTATCTCGAACTGACACT

GAGCCACAACCCAAACAACCCAGCTCTCCCTAAGCTTCAAACTAGACTACTTCTCCATAATATTCATCCC

TGTAGCATTGTTCGTTACATGGTCCATCATAGAATTCTCACTGTGATATATAAACTCAGACCCAAACATT

AATCAGTTCTTCAAATATCTACTCATCTTCCTAATTACCATACTAATCTTAGTTACCGCTAACAACCTAT

TCCAACTGTTCATCGGCTGAGAGGGCGTAGGAATTATATCCTTCTTGCTCATCAGTTGATGATACGCCCG

AGCAGATGCCAACACAGCAGCCATTCAAGCAATCCTATACAACCGTATCGGCGATATCGGTTTCATCCTC

GCCTTAGCATGATTTATCCTACACTCCAACTCATGAGACCCACAACAAATAGCCCTTCTAAACGCTAATC

CAAGCCTCACCCCACTACTAGGCCTCCTCCTAGCAGCAGCAGGCAAATCAGCCCAATTAGGTCTCCACCC

CTGACTCCCCTCAGCCATAGAAGGCCCCACCCCAGTCTCAGCCCTACTCCACTCAAGCACTATAGTTGTA

GCAGGAATCTTCTTACTCATCCGCTTCCACCCCCTAGCAGAAAATAGCCCACTAATCCAAACTCTAACAC

TATGCTTAGGCGCTATCACCACTCTGTTCGCAGCAGTCTGCGCCCTTACACAAAATGACATCAAAAAAAT

CGTAGCCTTCTCCACTTCAAGTCAACTAGGACTCATAATAGTTACAATCGGCATCAACCAACCACACCTA

GCATTCCTGCACATCTGTACCCACGCCTTCTTCAAAGCCATACTATTTATGTGCTCCGGGTCCATCATCC

ACAACCTTAACAATGAACAAGATATTCGAAAAATAGGAGGACTACTCAAAACCATACCTCTCACTTCAAC

CTCCCTCACCATTGGCAGCCTAGCATTAGCAGGAATACCTTTCCTCACAGGTTTCTACTCCAAAGACCAC

ATCATCGAAACCGCAAACATATCATACACAAACGCCTGAGCCCTATCTATTACTCTCATCGCTACCTCCC

TGACAAGCGCCTATAGCACTCGAATAATTCTTCTCACCCTAACAGGTCAACCTCGCTTCCCCACCCTTAC

TAACATTAACGAAAATAACCCCACCCTACTAAACCCCATTAAACGCCTGGCAGCCGGAAGCCTATTCGCA

GGATTTCTCATTACTAACAACATTTCCCCCGCATCCCCCTTCCAAACAACAATCCCCCTCTACCTAAAAC

TCACAGCCCTCGCTGTCACTTTCCTAGGACTTCTAACAGCCCTAGACCTCAACTACCTAACCAACAAACT

TAAAATAAAATCCCCACTATGCACATTTTATTTCTCCAACATACTCGGATTCTACCCTAGCATCACACAC

CGCACAATCCCCTATCTAGGCCTTCTTACGAGCCAAAACCTGCCCCTACTCCTCCTAGACCTAACCTGAC

TAGAAAAGCTATTGCCTAAAACAATTTCACAGCACCAAATCTCCACCTCCATCATCACCTCAACCCAAAA

AGGCATAATTAAACTTTACTTCCTCTCTTTCTTCTTCCCACTCATCCTAACCCTACTCCTAATCACATAA

CCTATTCCCCCGAGCAATCTCAATTACAATATATACACCAACAAACAATGTTCAACCAGTAACTACTACT

AATCAACGCCCATAATCATACAAAGCCCCCGCACCAATAGGATCCTCCCGAATCAACCCTGACCCCTCTC

CTTCATAAATTATTCAGCTTCCTACACTATTAAAGTTTACCACAACCACCACCCCATCATACTCTTTCAC

CCACAGCACCAATCCTACCTCCATCGCTAACCCCACTAAAACACTCACCAAGACCTCAACCCCTGACCCC

CATGCCTCAGGATACTCCTCAATAGCCATCGCTGTAGTATATCCAAAGACAACCATCATTCCCCCTAAAT

AAATTAAAAAAACTATTAAACCCATATAACCTCCCCCAAAATTCAGAATAATAACACACCCGACCACACC

GCTAACAATCAATACTAAACCCCCATAAATAGGAGAAGGCTTAGAAGAAAACCCCACAAACCCCATTACT

AAACCCACACTCAACAGAAACAAAGCATACATCATTATTCTCGCACGGACTACAACCACGACCAATGATA

TGAAAAACCATCGTTGTATTTCAACTACAAGAACACCAATGACCCCAATACGCAAAATTAACCCCCTAAT

AAAATTAATTAACCACTCATTCATCGACCTCCCCACCCCATCCAACATCTCCGCATGATGAAACTTCGGC

TCACTCCTTGGCGCCTGCCTGATCCTCCAAATCACCACAGGACTATTCCTAGCCATGCACTACTCACCAG

ACGCCTCAACCGCCTTTTCATCAATCGCCCACATCACTCGAGACGTAAATTATGGCTGAATCATCCGCTA

CCTTCACGCCAATGGCGCCTCAATATTCTTTATCTGCCTCTTCCTACACATCGGGCGAGGCCTATATTAC

GGATCATTTCTCTACTCAGAAACCTGAAACATCGGCATTATCCTCCTGCTTGCAACTATAGCAACAGCCT

TCATAGGCTATGTCCTCCCGTGAGGCCAAATATCATTCTGAGGGGCCACAGTAATTACAAACTTACTATC

CGCCATCCCATACATTGGGACAGACCTAGTTCAATGAATCTGAGGAGGCTACTCAGTAGACAGTCCCACC

CTCACACGATTCTTTACCTTTCACTTCATCTTGCCCTTCATTATTGCAGCCCTAGCAGCACTCCACCTCC

TATTCTTGCACGAAACGGGATCAAACAACCCCCTAGGAATCACCTCCCATTCCGATAAAATCACCTTCCA

CCCTTACTACACAATCAAAGACGCCCTCGGCTTACTTCTCTTCCTTCTCTCCTTAATGACATTAACACTA

TTCTCACCAGACCTCCTAGGCGACCCAGACAATTATACCCTAGCCAACCCCTTAAACACCCCTCCCCACA

TCAAGCCCGAATGATATTTCCTATTCGCCTACACAATTCTCCGATCCGTCCCTAACAAACTAGGAGGCGT

CCTTGCCCTATTACTATCCATCCTCATCCTAGCAATAATCCCCATCCTCCATATATCCAAACAACAAAGC

ATAATATTTCGCCCACTAAGCCAATCACTTTATTGACTCCTAGCCGCAGACCTCCTCGTTCTAACCTGAA

TCGGAGGACAACCAGTAAGCTACCCTTTTACCATCATTGGACAAGTAGCATCCGTACTATACTTCACAAC

AATCCTAATCCTAATACCAACTATCTCCCTAATTGAAAACAAAATACTCAAATGGGCCTGTCCTTGTAGT

ATAAACTAATACACCAGTCTTGTAAACCGGAGATGAAAACCTTTTTCCAAGGACAAATCAGAGAAAAAGT

CTTTAACTCCACCATTAGCACCCAAAGCTAAGATTCTAATTTAAACTATTCTCTGTTCTTTCATGGGGAA

GCAGATTTGGGTACCACCCAAGTATTGACTCACCCATCAACAACCGCTATGTATTTCGTACATTACTGCC

AGCCACCATGAATATTGTACGGTACCATAAATACTTGACCACCTGTAGTACATAAAAACCCAATCCACAT

CAACCCCCCCCCCCCATGCTTACAAGCAAGTACAGCAACCAACCCTCAACTATCACACATCAACTGCAGC

TCCAAAGCCACCTCTCACCCACTAGGATACCAACAAACCTACCCACCCTTAACAGTACATAGTACATAAA

GCCATTTACCGTACATAGCACATTACAGTCAAATCCCTTCTCGTCCCCATGGATGACCCCCCTCAGATAG

GGGTCCCTTGACCACCATCCTCCGTGAAATCAATATCCCGCACAAGAGTGCTACTCTCCTCGCTCCGGGC

CCATAACACTTGGGGGTAGCTAAAGTGAACTGTATCCGACATCTGGTTCCTACTTCAGGGCCATAAAGCC

TAAATAGCCCACACGTTCCCCTTAAATAAGACATCACGATG
